# Adaptive erasure of spurious sequences in sensory cortical circuits

**DOI:** 10.1016/j.neuron.2022.03.006

**Published:** 2022-06-01

**Authors:** Alberto Bernacchia, József Fiser, Guillaume Hennequin, Máté Lengyel

**Affiliations:** 1 Computational and Biological Learning Lab, Department of Engineering, University of Cambridge, Cambridge CB2 1PZ, United Kingdom; 2 MediaTek Research, Building 2010, Cambourne Business Park, Cambourne, CB23 6DW, United Kingdom; 3 Center for Cognitive Computation, Department of Cognitive Science, Central European University, Budapest 1051, Hungary

**Keywords:** cortical circuits, neural dynamics, Hebbian plasticity, excitation/inhibition, Dale's law, sequential activity, statistical adaptation

## Abstract

Sequential activity reflecting previously experienced temporal sequences is considered a hallmark of learning across cortical areas. However, it is unknown how cortical circuits avoid the converse problem: producing spurious sequences that are not reflecting sequences in their inputs. We develop methods to quantify and study sequentiality in neural responses. We show that recurrent circuit responses generally include spurious sequences, which are specifically prevented in circuits that obey two widely known features of cortical microcircuit organization: Dale’s law and Hebbian connectivity. In particular, spike-timing-dependent plasticity in excitation-inhibition networks leads to an adaptive erasure of spurious sequences. We tested our theory in multielectrode recordings from the visual cortex of awake ferrets. Although responses to natural stimuli were largely non-sequential, responses to artificial stimuli initially included spurious sequences, which diminished over extended exposure. These results reveal an unexpected role for Hebbian experience-dependent plasticity and Dale’s law in sensory cortical circuits.

## Highlights

• Recurrent circuits generate spurious sequences without sequential inputs
• A principled measure of total sequentiality in population responses is developed
• Theory predicts that Hebbian plasticity should abolish spurious sequences
• Spurious sequences in the visual cortex diminish with experience

## Teaser

Bernacchia et al. developed a theory of sequential activity in recurrent circuits that predicted the prevalence of “spurious” sequences in cortical activity that are unrelated to sequences in the inputs and their experience-dependent erasure by Hebbian plasticity. They confirmed these predictions in visual cortical recordings.

## Introduction

Sequential neural activity is believed to underlie a variety of functions (Abeles, 1991), such as learning (Hebb, 1949; Bi and Poo, 2001), perception (Wehr and Laurent, 1996), memory (Goldman, 2009; Ólafsdóttir et al., 2018), planning (Pfeiffer and Foster, 2013; Mattar and Daw, 2018), and motor control (Churchland et al., 2012). Indeed, it has been observed in a number of cortical areas (Hoffman and McNaughton, 2002; Mazzucato et al., 2015), including the hippocampus during both navigation and off-line periods (Skaggs and McNaughton, 1996; Diba and Buzsáki, 2007; Gupta et al., 2010; Carr et al., 2011; Lee et al., 2012), the cortical nuclei of songbirds during vocalization (Dave and Margoliash, 2000; Fee et al., 2004; Okubo et al., 2015), and the visual cortex (Ji and Wilson, 2007; Xu et al., 2012; Ekman et al., 2017). The learning of such sequential activity from experience has been proposed to be supported by specific circuit and synaptic plasticity mechanisms, such as spike-timing-dependent plasticity (STDP) (Fiete et al., 2010; Lengyel et al., 2005; Clopath et al., 2010; Gütig and Sompolinsky, 2006; Branco et al., 2010). However, the converse problem has so far been neglected: do spurious sequences, not reflecting sequences that ever appear in the input, also appear in cortical activity? If so, are there mechanisms in place that can erase them? Although such spontaneously generated activity sequences may in some cases be desirable in brain areas involved in motor control (Churchland et al., 2012; Okubo et al., 2015) or in areas undergoing development (Ackman et al., 2012), they will generally be disruptive to the normal operation of sensory areas by interfering with the temporal ordering of responses to external stimuli.

Inspired by the general phenomenon that unusual, often carefully crafted, inputs can result in spurious or atypical neural responses (Rossi et al., 1996; Grosof et al., 1993; Walker et al., 2019; Bashivan et al., 2019) and even lead to overt illusions (Brewster, 1844; Kanizsa, 1955), we hypothesized that sensory cortical circuits not adapted to the statistics of their inputs will be more prone to producing spurious sequences. Conversely, we wondered whether statistical adaptation would lead to the erasure of such spurious sequential activity.

In order to study the appearance and erasure of spurious sequences in sensory cortical circuits, we used a combination of theory, computational modeling of neural circuit dynamics, and analyses of neural recordings of the ferret visual cortex. We developed a measure of sequentiality for multivariate time series and derived theoretical conditions for a recurrent neural network to produce activity that is non-sequential by this measure. Surprisingly, we found that activity sequences tend to arise generically in recurrent networks, even when external inputs are non-sequential. Indeed, eliminating spurious sequences requires that a specific, non-trivial relationship be established between a network’s recurrent connectivity and the statistics of its input. We found that this relationship can be established and maintained via the joint effect of two widely known features of cortical microcircuit connectivity: Hebbian plasticity (connections between co-active cells are stronger) and Dale’s law (all efferent connections of a cell are either excitatory or inhibitory). We show that the Hebbian connectivity criterion can be achieved by STDP, which thus serves the erasure of spurious sequences rather than just the learning of new sequences. These theoretical results lead to two key predictions. First, neural responses to natural stimuli—to which connectivity has adapted—should not show spurious sequences. In contrast, artificial stimuli without *any* sequential structure should produce sequential responses that, due to Hebbian plasticity, should abate with experience. We confirmed these predictions directly in electrophysiological recordings from the primary visual cortex of awake ferrets. Taken together, our results establish the avoidance of spurious sequences as a key component of statistical adaptation in sensory circuits (Berkes et al., 2011) and elucidate the mechanisms by which the cortex solves this challenging computational task.

## Results

### Sequentiality in neural circuits

We began by considering a standard model of a recurrent neural network that describes how the instantaneous firing rate of each neuron changes over time (STAR Methods). Specifically, the momentary firing rate of each neuron *i* is a nonlinear, non-negative function $f\left(v_{i}\right)$ of an internal activation variable $v_{i}$, which models sub-threshold membrane potential fluctuations and evolves according to

(Equation 1) $$\tau \frac{dv_{i}}{dt}=-v_{i}\left(t\right)+\sum_{j=1}^{N}W_{ij}\;f\left[v_{j}\left(t\right)\right]+\xi_{i}\left(t\right)$$

where *τ* is the membrane time constant and $W_{ij}$ is the strength of the recurrent connection from neuron *j* to neuron *i*. In addition to recurrent synaptic interactions with other neurons in the network, each neuron *i* also receives a time-varying external input, $\xi_{i}\left(t\right)$. Our goal was to understand how statistical covariations in the inputs of pairs of neurons determine statistical covariations between their responses and, in particular, the presence or absence of any temporal ordering between those responses.

**Figure 1:**
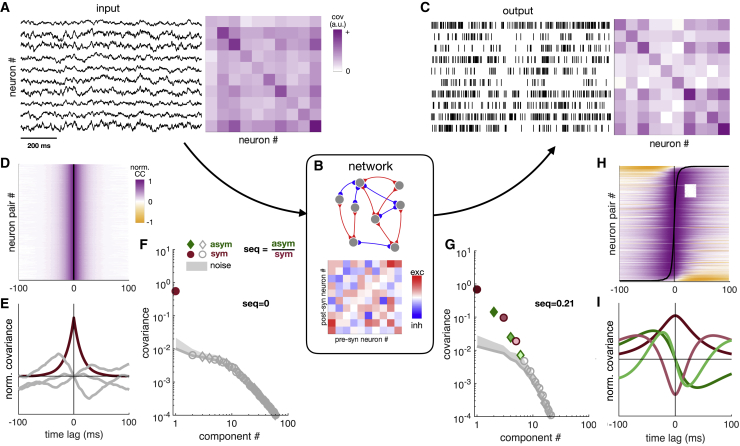
Sequentiality in symmetric neural circuits (A) Time series (left) and instantaneous covariance (cov) matrix of the inputs ($\Sigma =C^{\text{in}}\left(0\right)$, right) of 10 representative neurons (out of 50). (B) Schematic of the recurrent neural network model (top) and synaptic connection strengths of the same 10 neurons as in (A) (bottom). (C) Spiking output of the same 10 neurons as in A (left) and their covariances (right). (Exact spike times are notional, only firing rates are considered.) (D) Time-lagged CC functions of the inputs of all neuron pairs (each normalized to have a maximum magnitude of 1). For illustration, neuron pairs are ordered according to the location of the peak of their CCs (all at 0 in this case), and black line in the middle connects these peaks across cell pairs (all subsequent measures illustrated in E–G and I are independent of this ordering or the detection of peaks in CC functions). (E) The first four temporal components of the CC structure of the input to the network (red: symmetric, sym; green: anti-symmetric, asym; gray: noise). (F) Sequentiality spectrum: total covariance associated with each component of the input, in decreasing rank order (colors as in E). Gray line and shaded area show mean + 7 SD of covariances expected due to limited sample-size effects (STAR Methods). Sequentiality (seq) is computed as the ratio between the total covariance (root-sum-of-squares) carried by significant (non-noise) asymmetric versus symmetric components (Equation 3). (G–I) Same as (D)–(F) for the output of the network. See also Figure S1 illustrating the behavior of seq for artificial signals with precisely controlled properties and Figure S2 for similar results as shown here with other types of networks.

In general, natural stimuli may include sequences (e.g., due to self-motion-induced visual flow), potentially leading to sequential patterns of neural responses that could be difficult to identify as input driven rather than spurious. To be able to unequivocally identify any sequential activity appearing in the output of the network as spurious, we first focused on the simple case in which the input to the network had no sequences (Figure 1A). Specifically, we precluded any temporal ordering between network inputs by enforcing temporal symmetry in the input covariance: $C_{ij}^{\text{in}}\left(\tau \right)=C_{ij}^{\text{in}}\left(-\tau \right)$, where $C_{ij}^{\text{in}}\left(\tau \right)=\text{cov}\left[\xi_{i}\left(t\right),\xi_{j}\left(t+\tau \right)\right]$ is the covariance between the inputs of neuron *i* and *j* at a time lag *τ*. As we show below, networks that successfully avoid spurious sequences in this non-sequential input regime can also more faithfully represent temporally ordered information in the regime in which inputs are sequential.

As an extreme case, we first considered a network in which connections between neurons were random but entirely symmetric, such that $W_{ij}=W_{ji}$ and the network, therefore, did not embed any directed chains (Goldman, 2009; Figure 1B). Recurrent interactions between the neurons of the network through these connections produced complex, time-varying responses (Figure 1C). The high dimensionality of these responses required us to develop a principled method to quantify how much (if any) sequential activity was present in them (STAR Methods; Note S1).

Our method is based on simultaneously analyzing the time-lagged cross-covariances (CCs) of the responses of all cell pairs in a population, $C_{ij}\left(\tau \right)=\text{cov}\left[f\left(v_{i}\left(t\right)\right),f\left(v_{j}\left(t+\tau \right)\right)\right]$. Although, by construction, all CCs in the input were time symmetric (Figure 1D), the CCs in the output showed marked asymmetries, resulting in a clear temporal ordering across cell pairs (Figure 1H). Our method decomposes this population of CCs into a set of separable spatio-temporal components, using a computationally efficient and principled generalized singular value decomposition-based approach:

(Equation 2) $$C_{ij}\left(\tau \right)=\sum_{k=1}^{N^{2}}\sigma_{k}\;A_{ij}^{\left(k\right)}\;f^{\left(k\right)}\left(\tau \right)$$

where the $\sigma_{k}$ are positive constants scaling the overall covariance accounted for by each component, $A^{\left(k\right)}$ are (normalized and pairwise orthogonal) spatial components, and $f^{\left(k\right)}\left(\tau \right)$ are (normalized and pairwise orthogonal) temporal components. Importantly, we were able to show that each of the temporal components, $f^{\left(k\right)}\left(\tau \right)$, is guaranteed to be either exactly symmetric or anti-symmetric (Figures 1E and 1I; Note S1). Following standard approaches (Machens et al., 2010), we then separated out the genuinely present “signal” components from those “noise” components that arise from measurement errors and finite recording durations, based on their overall covariance, $\sigma_{k}$ (Figures 1F and 1G; STAR Methods).

Although in the example we are considering here, the time courses of signal components in the input were all time symmetric (Figure 1E), the output contained several signal components that were temporally anti-symmetric (Figure 1I). To quantitatively compare the sequentiality of input and output, we defined the overall level of sequentiality of each time series (“seq”) as the total contribution (root-sum-of-squares) of all its anti-symmetric (“asym”) signal components (normalized by the contribution of its symmetric signal components, “sym”; Figures 1F and 1G):

(Equation 3) $$\text{seq}=\sqrt{\frac{\sum_{k\in \text{asym}}\sigma_{k}^{2}}{\sum_{k\in \text{sym}}\sigma_{k}^{2}}}$$

This measure of sequentiality is zero when all covariances are time symmetric (up to noise), and its maximum is one, which can be achieved by a population that persistently generates a single sequence—as in so-called synfire chains (Abeles, 1991; Figures S1A–S1C). (Note that this measure is largely independent of the presence of oscillations as such, as it can be high even in networks that do not oscillate and can be low even in oscillatory networks when neurons do not have consistent phase relationships; Figure S1D.)

As expected, in the example we studied here, sequentiality was zero for the input by our measure (Figure 1F). However, despite the network being symmetric, the output displayed substantial sequentiality (seq = 0.21; Figure 1G). These results, showing the generation of (spurious) sequential activity in response to non-sequential inputs, generalized to networks with random non-symmetric or random anti-symmetric connections, as well as to random “Dale” networks composed of split populations of excitatory and inhibitory neurons (Figure S2).

### A theory of spurious sequences

**Figure 2:**
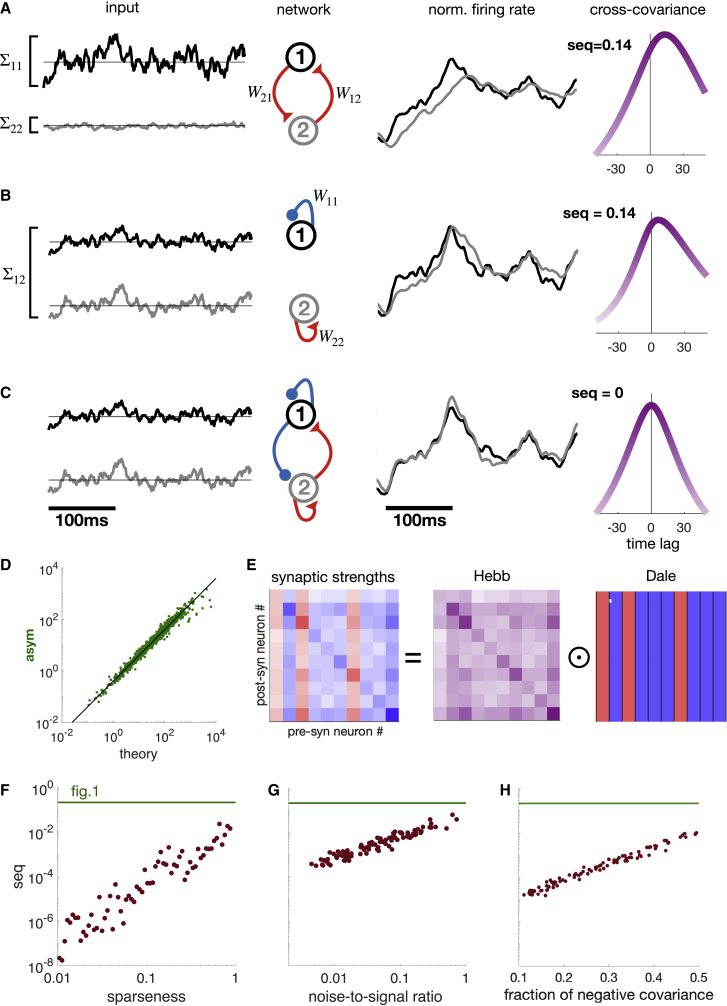
A theory of spurious sequences (A) A two-unit network with symmetric excitatory connections ($W_{12}=W_{21}>0$, middle left), receiving inputs of unequal magnitude (variances $\Sigma_{11}>\Sigma_{22}$, far left), produces outputs (shown as normalized firing rates, middle right) such that unit 2 (gray) trails unit 1 (black), as also shown by their CC function and the resulting sequentiality (far right). (B) Same as (A), for a network of two units with only self-connections of opposite signs ($W_{11}<0<W_{22}$, middle left), receiving strongly correlated inputs (covariance $\Sigma_{12}\gg 0$, far left). (C) Same as (B), after adding specific asymmetric connections between the two units of the network (middle left), making its output temporally symmetric (middle and far right). (D) Theoretical prediction of Equation 4 matches well the actual total anti-symmetry of the activity of 2-node symmetric networks. 1,000 randomly generated networks are shown (green dots). Black diagonal is the identity (note that both axes are logarithmic). (E) Theoretical condition for avoiding spurious sequences: a network of any size produces no sequentiality if its synaptic strengths are the product of a term that is proportional to neural correlations (Hebb) and a term that has the same sign for all efferent synapses of each neuron (Dale, ⊙ denotes element-wise product of matrices). (F–H) Sequentiality (seq) for more realistic networks whose connectivity increasingly deviates from the theoretical “Hebb-and-Dale” optimum (cf. E). In all cases, 100 random 50-neuron networks were first generated according to the theory and their connectivity was then perturbed (red dots). Green line shows sequentiality of the network shown in Figure 1 for reference, note the order-of-magnitude difference (logarithmic y axis). (F) Sparseness: a fraction of the connections were removed, starting from the weakest and resulting in sparser connectivity. (G) Imprecision of Hebbian plasticity (noise-to-signal ratio): noise was added to the original Hebbian component (signal) of synaptic strengths. (H) Negative correlations: a fraction of input correlations were negative, and the corresponding connections were removed to preserve Dale’s law. See also Figure S3 comparing the responses of Hebb-and-Dale networks with those of various random networks to sequential inputs, and their respective behaviors at a range of intrinsic network time constants for non-sequential inputs.

Given the ubiquity of spurious sequences, as indicated by our simulations, we wondered what mechanisms explained their appearance even under conditions that seemed to maximally work against them (i.e., non-sequential input and symmetric connections). For this, we began by considering a minimal, symmetrically connected circuit motif with only two units, representing individual neurons or small populations. We found that sequentiality arose mainly from two independent properties of the model. First, when these two units received inputs that were unequal in their magnitudes, such that unit 1 received much larger inputs than unit 2, unit 2 became mainly entrained (with some lag) by unit 1 rather than by its own external input (Figure 2A). As a result, the activity of unit 2 trailed that of unit 1. Second, even with balanced input magnitudes, systematic lags between units could also develop due to unequal self-connection strengths (Figure 2B). Self-connections alter the effective time constant with which neural populations integrate their inputs, such that larger self-connections result in slower input integration (Seung, 1996). Thus, in our example, unit 2 with excitatory self-connections trailed unit 1 with inhibitory self-connections. We were able to show (Figure 2D; Note S2) that the presence and magnitude of these two basic motifs together accurately predict the appearance of spurious sequences (i.e., the total amount of temporal anti-symmetry, quantified by the numerator of our sequentiality measure, Equation 3) in a simple mathematical form:

(Equation 4) $$\sqrt{\sum_{k\in \text{asym}}\sigma_{k}^{2}}\;\;\tilde{\propto }\;\;\left|\underset{\text{motif}\;\text{A}}{\underset{︸}{\left({\text{Σ}}_{22}-{\text{Σ}}_{11}\right)\;W_{12}}}+\underset{\text{motif}\;\text{B}}{\underset{︸}{\left(W_{11}-W_{22}\right)\;{\text{Σ}}_{12}}}\right|$$

where $\Sigma_{ij}=C_{ij}^{\text{in}}\left(0\right)$ is the instantaneous covariance of the inputs of units *i* and *j*. The two motifs described above respectively correspond to the first and second terms of this sum.

Importantly, further analysis also identified specific asymmetric connections which, when included in the network, allowed the effects of the two basic motifs to cancel, resulting in zero sequentiality (Figure 2C; STAR Methods). We noted that the primary effect of these additional asymmetric connections was to match the connectivity of the network to the input correlations between neurons, such that the network came to comply with two fundamental principles of the organization of cortical circuits: (1) Hebb’s postulate by which connection strengths between neurons are expected to be proportional to their correlations (Hebb, 1949) and (2) Dale’s rule, which results in each neuron either exciting or inhibiting all of its postsynaptic targets (Eccles, 1986).

For larger networks (with more than two neurons), the interplay between network connectivity, input statistics, and our sequentiality measure is more complex (STAR Methods; Note S2). Nevertheless, the motifs we identified for two-neuron networks are still relevant. For example, seq = 0 for any network with symmetric connectivity, homogeneous input variances (i.e., absence of motif 1), and homogeneous self-connections (i.e., absence of motif 2). Most importantly, our mathematical theory of sequentiality in non-linear recurrent neural circuits of arbitrary size revealed that the principles of Hebb and Dale continue to predict the erasure of spurious sequences. Specifically, we were able to prove that for any non-sequential input, sequentiality in the output of a network is zero if each connection strength in the network is the product of (1) the covariance between the inputs to the pre- and postsynaptic neurons (“Hebb”) and (2) a positive or negative constant that depends only on the presynaptic neuron (“Dale”; Figure 2E). In Note S2, we show that the first factor can alternatively be replaced by the output (instead of input) covariance. Thus, as long as correlations are non-negative, this “Hebb-and-Dale” construction results in connections that satisfy Dale’s law.

We verified in numerical simulations that non-sequentiality of the outputs is preserved even when connectivity is sparse (Figure 2F), when connection strengths are only approximately proportional to input covariances (Figure 2G) or when some fraction of input covariances is negative but synapses corresponding to these covariances are pruned to preserve Dale’s principle (Figure 2H; seq < 0.01 up to 90% sparseness, 20% noise-to-signal ratio, or 50% negative input covariances). These results show that Dale’s law in tandem with Hebb’s rule is sufficient for eliminating spurious sequences even in these more realistic scenarios, with only minor, if any, modifications. Interestingly, even when modifications are necessary, these simply result in eliminating some of the synaptic connections, thus improving the biological plausibility of the network, which may otherwise need to be fully connected.

Although our focus was primarily on the erasure of spurious sequences in response to purely non-sequential inputs, we also confirmed that Hebb-and-Dale connectivity remains useful even in the more general case, when the input to a network can contain sequences. Specifically, we found that more sequential inputs resulted in increasingly sequential outputs in this case (Figure S3A) and that output sequences represented input sequences substantially better than in networks with random connectivity (matched for basic properties of the resulting network dynamics; Figure S3B). In addition, for non-sequential inputs, we could also show that Hebb-and-Dale networks maintain zero sequentiality over a broad range of intrinsic network time constants. This was particularly notable in networks with connections strong enough as to induce long (but finite) timescales in their dynamics (Ganguli et al., 2008), as networks with random connectivity produced strongly sequential inputs in that case (Figure S3C).

### Hebbian plasticity erases spurious sequences

**Figure 3:**
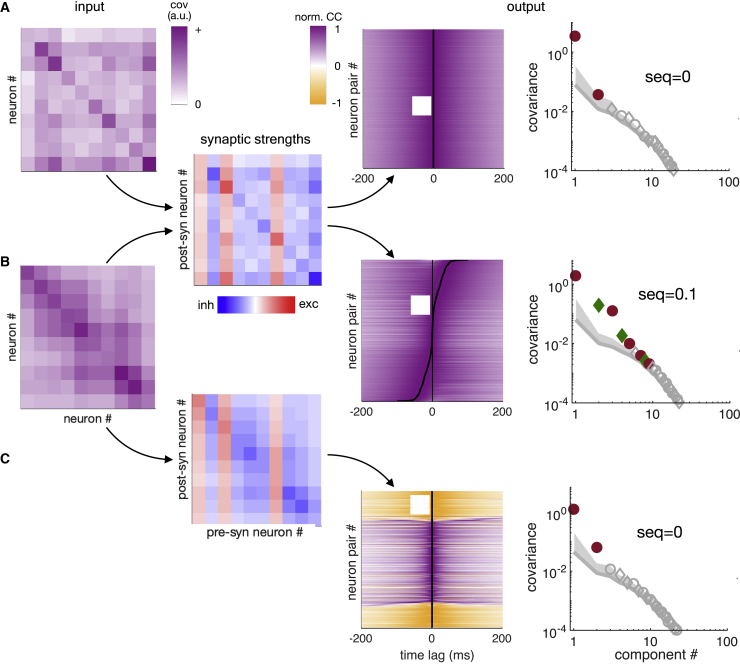
Adaptive erasure of spurious sequences in a model network (A) A neural network with Hebb-and-Dale synaptic connections (middle, cf. Figure 2E) receiving input to which it has undergone Hebbian adaptation (left, input covariance shown as in Figure 1A) produces non-sequential output (right, CCs and sequentiality spectrum shown as in Figures 1G and 1H). (B) The same network as in (A) receiving input with a different input covariance structure (left) produces sequential output (right). (C) The network receiving the same input as in (B) but after its synaptic connections adapted to the input covariance structure (middle) once again produces non-sequential output (right).

Based on our theoretical results, we predicted that excitatory-inhibitory cortical circuits (which already satisfy the theory’s Dale requirement) will exhibit or eliminate spurious sequences depending on whether their connection strengths have undergone Hebbian adaptation to their inputs. To test these predictions, we first simulated a network whose synaptic connections were of Hebb-and-Dale form as required by the theory (Figure 3A, left and middle). As predicted, its output was time symmetric (seq = 0; Figure 3A, right). We then simulated the effect of a sudden change of input statistics by feeding the network with an input that had the same non-sequential temporal statistics but a different set of pairwise correlations between neurons, all the while keeping synaptic connections in the network unchanged (Figure 3B, left). As a result, the network exhibited spurious sequential activity (seq = 0.1; Figure 3B, right). Finally, we simulated the effects of Hebbian plasticity in the network under these changed input conditions by setting the connection strengths to be proportional to the new input correlations (Figure 3C, middle). The adapted network again showed no spurious output sequences (seq = 0; Figure 3C, right).

**Figure 4:**
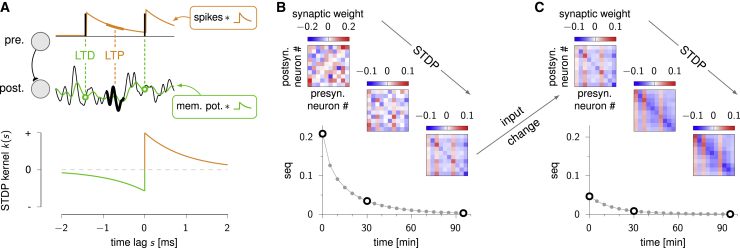
STDP adaptively eliminates spurious sequences (A) Top: schematic with a pair of synaptically connected cells (left) and with a sample of their activity and quantities determining synaptic modification according to a standard model of STDP (Clopath et al., 2010; right). Each presynaptic spike (top, black ticks) contributes an exponentially decaying waveform (orange inset) to the synaptic eligibility trace (top, orange curve). The postsynaptic membrane potential (bottom, black trace) is passed through a low pass filter (green inset) to produce a running average (bottom, green trace). The synapse is strengthened continually (orange, LTP) in proportion to the presynaptic eligibility trace and the (unfiltered) postsynaptic membrane potential and weakened at the time of presynaptic spikes (green, LTD) in proportion to the filtered postsynaptic membrane potential. Bottom: effective STDP kernel, k(s), describing the change in synaptic strength as a function of the lag between pre- and postsynaptic activity, *s*. Orange half is the presynaptic eligibility waveform, green half is the time- and sign-reversed postsynaptic filter waveform. Rule shown is for synapses in which the presynaptic cell is excitatory, the same rule with the opposite sign applies to synapses with inhibitory presynaptic neurons. Beside the additive term shown here, synaptic modifications also included a (multiplicative) weight decay (not shown), ensuring that synaptic strengths remained bounded (STAR Methods). (B) Sequentiality index during the course of STDP, starting from the connectivity matrix shown in Figure 1B and under the input statistics of Figure 1A. Insets show snapshots of the synaptic connectivity matrix at the times shown by the three large black circles. Note the similarity of the final weight matrix to the direct Hebb-and-Dale solution of Figure 3A. (C) Continuation of (B), after a sudden change in input statistics to that of Figure 3B. Note the similarity of the final weight matrix to the direct Hebb-and-Dale solution of Figure 3C. For efficiency, we did not simulate individual spikes in (B) and (C). Instead, we used a rate-based description of neural responses and an analytical approach to compute both the corresponding average changes in synaptic connections (Kempter et al., 1999; Dayan and Abbott, 2001) and the resulting sequentiality index (STAR Methods; Note S3). Stability of STDP learning dynamics is illustrated in Figure S4.

What form of plasticity might give rise to the Hebb-and-Dale synaptic connectivities that are necessary for avoiding spurious sequences? We reasoned that STDP, a prototypical form of Hebbian plasticity (Bi and Poo, 2001; Gerstner et al., 1996; Morrison et al., 2008), may be a natural candidate. Intriguingly, we were able to show that the connectivities produced by STDP do not have the specific Hebb-and-Dale form we have studied so far. In particular, despite STDP changing synaptic connectivities depending on neuronal output covariances, the “Hebb” part of the resulting connectivities is not simply proportional to neuronal input or output covariances (Figure 2C; Note S3). Nevertheless, we were also able to show that, under broad conditions, STDP gives rise to more general forms of Hebb-and-Dale connectivities that still guarantee the absence of spurious sequences. This is true irrespective of the precise shape of the function describing the dependence of potentiation and depression on the relative timing of presynaptic spikes and postsynaptic activity (Figure 4A), so long as synaptic strengths remain bounded and STDP acts on excitatory and inhibitory synapses alike (with opposite signs; Vogels et al., 2013; Hennequin et al., 2017; see also Figure S4). To illustrate this, we revisited the example of Figure 3, now using STDP to explicitly model the time course with which synaptic connectivity and thus the sequentiality of network responses changes over time (Figures 4B and 4C). Specifically, we assumed that the network’s inputs had the same statistics as those used in Figure 3A, but we initialized the connectivity of the network to the non-Hebb-and-non-Dale configuration shown in Figure 1B. This resulted in initially sequential activity (seq ≃ 0.2), which gradually decayed to zero with the ongoing action of STDP (Figure 4B). In fact, synaptic connections converged to a very similar configuration as in the direct Hebb-and-Dale solution we used above (Figure 3A). Note that in this example, the initial connectivity did not respect Dale’s law but the final connectivity did. Thus, STDP in the model not only tuned the strengths of synapses to make them Hebbian with respect to the stimulus statistics but occasionally also flipped their signs such that the network came to comply with Dale’s law. Finally, following a sudden change in input statistics and thus a sudden jump in sequentiality, as in Figure 3B, ongoing STDP gradually adjusted the synaptic strengths such that spurious sequences were once more eliminated (Figure 4C). Indeed, synaptic connections reached a configuration similar to the direct Hebb-and-Dale solution of Figure 3C. In this case, the Dale structure of connectivity was preserved throughout adaptation, while connection strengths gradually became Hebbian with respect to the new stimulus statistics.

### Adaptive erasure of spurious sequences in the ferret visual cortex

**Figure 5:**
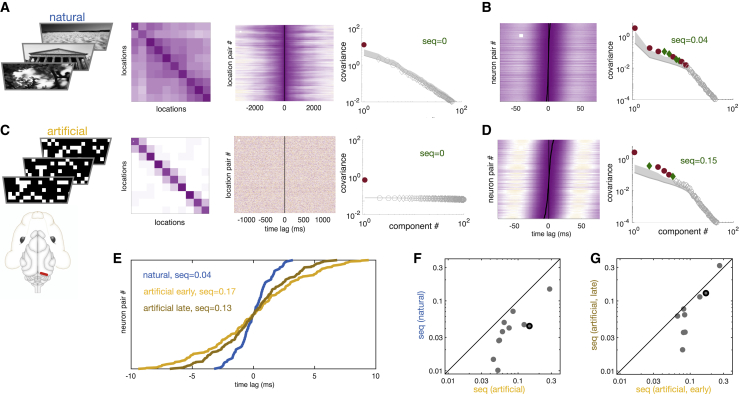
Adaptive erasure of spurious sequences in the ferret visual cortex (A) Natural movie stimuli: three example frames (far left), the input covariance (middle left), CCs (middle right), and sequentiality spectrum (far right) for 10 representative pixels (cf. Figures 1A, 1D, and 1F). Stimuli shown are for illustration only; stimuli actually used are not shown for copyright reasons (STAR Methods). Analysis shown is for actual stimuli. (B) Neural responses to natural video stimuli in a representative animal: CCs (left) and sequentiality spectrum (right) for the 16 channels recorded (cf. Figures 1H and 1G). Sequentiality is low (seq = 0.04). (C and D) Same as (A and B) for artificial (block noise) stimuli. Sequentiality of neural responses is higher (seq = 0.15). Red strip in ferret head schematic illustrates approximate electrode location. (E) Adaptation to continued exposure to artificial stimuli. Peaks of CCs for the same animal (cf. black line in B and D): sequentiality is higher in the first half of the experimental session (yellow, seq = 0.17) and decreases in the second half of the experimental session (brown, seq = 0.13). CC peaks for natural stimuli are shown for reference (blue, seq = 0.04). (F) Sequentiality of neural responses to artificial (y axis) versus natural stimuli (x axis) across animals (dots). Sequentiality is lower for natural stimuli for all animals (p = 0.0001, n = 11). (G) Sequentiality of neural responses during the late (y axis) versus the early half of exposure (x axis) to the artificial stimuli across animals (dots). Sequentiality is lower later in all animals but one (p = 0.037, n = 9 after excluding two animals whose sequentiality fell to zero due to limited sample size when restricting analysis to half of the data). See Figure S5 for the same analysis for responses to natural stimuli. Dots with black contour in (F) and (G) indicate the animal whose data are shown in (B), (D), and (E).

Finally, we analyzed neural recordings in the primary visual cortex (V1) of awake ferrets to test the experience-dependent erasure of spurious sequences that our theory and simulations predicted (Figure 5). For this, we used neural activity in animals between postnatal days 44 and 151, i.e., after the maturation of orientation tuning and long-range horizontal connections (Sengpiel and Kind, 2002). As stimuli, we used natural-scene movies (Fiser et al., 2004; Berkes et al., 2011), which showed symmetric temporal correlations and thus no sequentiality by our measure (seq = 0, Figure 5A). Using such non-sequential stimuli was consistent with our theoretical analyses and ensured that any sequentiality in neural responses could be unequivocally identified as spurious. We expected the V1 of these animals to have already been adapted to the statistics of these stimuli. In line with our predictions (cf. Figure 3A), V1 activity showed only very weak sequentiality in this case (Figures 5B and 5F, seq = 0.04 for the animal shown). We then used random block noise stimuli, which were strictly temporally symmetric by construction (seq = 0; Figure 5C) and to which we expected V1 not to have adapted (cf. Figure 3B). Again, in line with predictions, V1 responses became substantially and significantly more sequential than responses to natural stimuli, despite the stimuli being perfectly non-sequential in this case (seq = 0.15, Figure 5D versus Figure 5B; Figure 5F, 11 animals showing non-zero sequentiality, p = 0.0001, paired t test). Moreover, when comparing the first and last 12.5 min of stimulation with these artificial stimuli, we also found a small but significant decrease in sequentiality over continual stimulation (Figure 5E, yellow versus brown; Figure 5G, p = 0.037, paired t test), in line with our predictions about the effects of ongoing adaptation to novel inputs (cf. Figure 3C). As a control, there was no such difference between early and late stimulation with natural stimuli (Figure S5, p = 0.29, paired t test), excluding the possibility that the temporal change we found for artificial stimuli was simply due to general fatigue-, arousal-, or electrode displacement-based (or similar, stimulus-independent slow-timescale) effects.

## Discussion

For neural circuits to reliably process sequential sensory information, they need to both store experience-dependent sequences and avoid generating spurious sequences. While most previous work focused on how experience-driven sequences are stored and recalled (Abbott and Blum, 1996; Lengyel et al., 2005; Fiete et al., 2010; Brea et al., 2011), our results highlight the potential prevalence of spurious sequential activity in neural circuits and provide theoretic tools for its study. Hebbian plasticity has been well established as a key component in storing sequential information (Abbott and Blum, 1996; Lengyel et al., 2005; Fiete et al., 2010; Brea et al., 2011). Our analyses show, for the first time, that Hebbian mechanisms can also eliminate spurious sequences when acting in conjunction with Dale’s law. These results suggest that the two main organizing principles of cortical circuits, Hebbian plasticity and Dale’s law, together play an important role in sequential information processing and, more broadly, in the statistical adaptation of sensory cortical responses (Berkes et al., 2011).

### Measuring neural sequentiality

In order to quantify sequentiality in the responses of a neural population rigorously, in a way that is directly relevant to testing the predictions of our theory, we developed a measure of sequentiality. There have been multiple previous proposals for characterizing sequences in neural population responses. Some of these methods focus on identifying individual sequences within a distribution of responses, which were recorded over multiple trials or over an extended time period (Nádasdy et al., 1999; Ikegaya et al., 2004; Mackevicius et al., 2019). Another set of methods measures the sequentiality of responses in individual trials, rather than across a distribution, by assessing the presence of particular pre-defined spatio-temporally regular motifs (Orhan and Ma, 2019; Zhou et al., 2020). Whether the motifs or sequences are pre-defined or identified in a data-driven way, both these sets of methods provide an overall measure of sequentiality, if at all, only once individual motifs or sequences have been reliably determined. In contrast, our method sidesteps determining particular motifs or sequences and provides a direct measure of sequentiality. The advantage of our method is that it does not require strong prior assumptions about the nature of motifs in neural responses while also avoiding the sensitivity of sequence identification to noise (Mackevicius et al., 2019). It achieves this by automatically separating sequential, non-sequential, and noise components of the high-dimensional time series of the inputs and outputs of a neural circuit.

An obvious disadvantage of our method is that when responses are sequential, it alone does not identify the actual sequences they contain—though such extensions are possible (Rutten et al., 2020a, 2020b). Furthermore, while other methods effectively assess sequentiality via complex nonlinear features, our method only uses second-order information. Although this restriction enabled us to develop a complete analytical theory of sequentiality in stochastic recurrent neural networks, there are cases where activity sequences cannot be detected in spatio-temporal covariances alone. In particular, if a circuit generated a sequence in the forward and reverse directions in approximately equal measure, its responses would erroneously appear to be non-sequential when quantified by our method. Nevertheless, it is unclear how severe a limitation this is in practice. For example, when analyzing hippocampal place cell responses while an animal traverses the same linear track back-and-forth, one might expect a balanced distribution of forward and reverse neural sequences corresponding to the two running directions. However, in such cases, outward and inward journeys activate two largely non-overlapping ensembles of place cells instead of the same ensemble (McNaughton et al., 1983). Moreover, during periods of inactivity, although the replay of either of these sequences in the hippocampus is known to occur in both the forward and reverse direction, forward replay is significantly more frequent than reverse replay (Diba and Buzsáki, 2007; Davidson et al., 2009), thus again avoiding the collapse of second-order sequentiality, which could otherwise mislead our method.

### Sequential versus non-sequential inputs

In our analyses, we primarily focused on the cases where inputs to a sensory cortical population are non-sequential. There is good reason to believe that this scenario may not be unrealistic, and natural stimuli will generally lead to temporally symmetric input covariances. First, whereas different neurons in an upstream area may have different onset latencies, realistic levels of divergence and convergence of connections between the two areas can effectively average out these differences. Second, onset latencies are usually established using artificial stimuli with hard onsets (Euler and Masland, 2000; Nowak et al., 1995), which rarely occur in natural stimuli. Conversely, the smoother transitions in natural stimuli will typically diminish differences between the onset latencies of different neurons. Third, different delays between the inputs of different cells in naturalistic conditions can be expected to be dominated by the (temporal) statistics of natural stimuli (which may sequentially excite the receptive fields of appropriately ordered neurons), rather than different physiological delays. Indeed, with sequentially presented stimuli, onset latencies were found to be history dependent (Bair et al., 2002). Critically, the statistics of these input sequences can easily average out in natural stimuli. Our analysis showing the lack of consistent sequentiality in naturalistic video stimuli at the level of pixels (Figure 5A) and V1 activity in response to these stimuli (Figure 5B) provides support for this effect. Nevertheless, we also studied the case of non-sequential inputs and found Hebb-and-Dale networks to show distinct functional advantages even in that domain (Figure S3A).

### Negative neural correlations and Dale’s law

Our theory predicts that in “Hebb-and-Dale” connectivities that successfully eliminate spurious sequences, Dale’s law only arises provided the covariances in the Hebbian component are all non-negative. These covariances correspond to “signal” correlations in studies of neural coding (Averbeck et al., 2006) and may be negative in sensory areas (e.g., due to ON-OFF opponency; Mastronarde, 1989). As such, they appear to compromise the role of Dale’s law in the suppression of spurious sequentiality. However, in our formalism, the experimentally found negative signal correlations relate to output, not input, covariances. Although balanced network dynamics tend to yield equal proportions of positive and negative output correlations (Renart et al., 2010), the correlations between the total external inputs received by different neurons are likely to be large and positive on average due to the shared and excitatory nature of these inputs. Importantly, according to our theory, recurrent connections adhering to Dale’s law guarantee the absence of spurious sequences when input covariances are positive.

### STDP and Dale’s law

Our analysis of the effect of STDP on synaptic connectivity suggests an unexpected potential connection between STDP and Dale’s law. STDP is commonly thought of as an activity-dependent plasticity mechanism that ensures that the efficacies of synapses obey Hebbian principles (more co-active neurons are more strongly connected; Bi and Poo, 2001). Our results confirmed this role for STDP. However, we showed that when inputs to a network are non-sequential, STDP can also ensure that the “signs” of connections in a network respect Dale’s law, i.e., that all efferent synapses of a cell are either excitatory or inhibitory. Dale’s law is usually considered to be developmentally predetermined via the primary neurotransmitter released by a given cell type (Kandel et al., 2013). Nevertheless, there is evidence that it can also be altered by plasticity. For example, neurotransmitter expression has been shown to be under the control of activity-dependent mechanisms (Spitzer, 2012). Moreover, the postsynaptic action of even the same neurotransmitter—the ultimate determinant of the “sign” of a synapse—might depend on the receptors expressed postsynaptically and local ionic driving forces. Changes in each of these factors have been shown to be able to switch between excitation and inhibition in synapses using the same neurotransmitter (Liu and Wilson, 2013; Raimondo et al., 2012). It remains to be demonstrated whether some combination of these (and perhaps some yet-unknown) mechanisms are sufficient to control the sign of individual synapses in an activity-dependent manner and in accordance with Dale’s law, in the way STDP in our model can.

### Experimental tests

Based on our theoretical predictions, we found experimental support for the adaptive erasure of spurious sequences in ferret V1. This data set was ideal for testing our theory, as it satisfied three important requirements. First, recordings need to be performed during normal operation (as opposed to, e.g., development or off-line periods) in a sensory area (as opposed to motor areas) such that the erasure of spurious sequences can be expected to be a priority for the underlying circuit. Second, recordings need to be sufficiently long to allow the reliable estimation of CCs, which in general are noisy such that acceptable signal-to-noise ratios require averaging over many trials (or, equivalently, long recordings). In fact, what matters primarily is the overall number of action potentials recorded per neuron, with more quiet neurons typically calling for longer recordings. We estimate that our approach requires, on average, several tens of thousands of spikes in each individual unit. Third, the temporal resolution of our ferret data set was fine enough that we could reliably estimate the sequentiality index. In particular, it is important that the sampling frequency be large enough to enable the detection of systematic lags between neurons, even when their CCs peak at short lags. In the ferret data used in this study, multi-unit recordings have a resolution of 2 ms, and the asymmetries in the cross-correlations occur on a timescale of about 10 ms. Detecting such asymmetries would be harder in, e.g., calcium imaging data, which suffer from poorer temporal resolution.

A straightforward prediction of our theory is that rearing animals under artificial stimulus statistics (e.g., block noise) should revert our main experimental result and lead to less sequentiality for that specific class of artificial stimuli and more sequentiality when tested with natural stimuli (i.e., dots above rather than below the diagonal in Figure 5F). Alternatively, in normally reared animals, more extensive stimulation with artificial stimuli should not only lead to less sequentiality in the late period of the artificial condition (i.e., dots further below the diagonal than in Figure 5G) but eventually also to an increase in sequentiality during (at least the early period of) the natural stimulus condition (blue line in Figure 5E becoming less steep). In fact, simply introducing another artificial stimulus statistics following adaptation to the first one should also lead to an (at least transient) increase in sequentiality. This would also eliminate a potential shortcoming in our current approach, which uses only one class of artificial stimuli. Namely, we found a decrease of sequentiality for the artificial stimuli over time and, as a control, that no such decrease occurs for natural stimuli. However, we cannot exclude the possibility that this difference is due to more fundamental differences in the ways natural and artificial stimuli are processed upstream of V1, which are beyond the differences between their covariance structure and which we did not control. Comparing the processing of two different but “equally” artificial stimulus sets instead would provide a stronger control.

Finally, our theory also predicts that inhibitory synaptic strengths should be proportional to neural correlations (Figure 2E). This prediction has recently been confirmed in the mouse visual cortex (Ko et al., 2011; Cossell et al., 2015; Znamenskiy et al., 2018) and corroborated by Hebbian synaptic plasticity rules found in GABAergic synapses (Vogels et al., 2013; Hennequin et al., 2017). Together with earlier results on experience-dependent sequential activity, our work lays the foundations for a unified understanding of how circuits can use neural sequences to faithfully represent temporal ordering of information in the environment and to drive sequential behavior.

## STAR★Methods

### Key resources table

**Table:** 

REAGENT or RESOURCE	SOURCE	IDENTIFIER
**Deposited data**		

Raw and analyzed data	This paper	https://doi.org/10.5281/zenodo.6124683

**Experimental models: Organisms/strains**		

13 male sable ferrets (*Mustela putorius furo*) at different stages of visual development ranging from P44 to P151.	The Fiser Laboratory	Wild type

**Software and algorithms**		

MATLAB	Mathworks	R2018b (9.5.0.944444)
Code for computing sequentiality	This paper	https://doi.org/10.5281/zenodo.6124314

### Resource availability

#### Lead contact

Further information and requests for resources should be directed to and will be fulfilled by the lead contact, Alberto Bernacchia (ab2349@cam.ac.uk).

#### Materials availability

This study did not generate new unique reagents.

### Experimental model and subject details

For the experiments reported in Figures 5 and S5, 13 male sable ferrets (*Mustela putorius furo*) were used at different stages of visual development ranging from P44 to P151. All experimental procedures and handling of animals were approved by the University of Rochester Committee on Animal Research and performed in compliance with National Institutes of Health guidelines.

### Method details

#### Animal preparation and data acquisition

The details of the procedures have been described in detail previously (Fiser et al., 2004; Chiu and Weliky, 2001; Berkes et al., 2011). Briefly, a linear array of 16 electrodes, spaced by 200 micrometers, was implanted in layer 2-3 of the primary visual cortex (V1) under isoflurane anesthesia. The electrodes typically provided clear multi-unit (and occasionally single-unit) signal on each channel. The signal was pre-processed by band-pass filtering (600-6000 Hz) and digitized at 10 kHz. Spike discrimination was performed offline by manually setting a separate voltage threshold for each electrode. Stable recordings were maintained for 8-12 hours.

#### Visual stimulation

Shortly after recovery from surgery, neural activity in response to different stimulus ensembles was recorded in awake animals. Animals rested on a padded platform with their head fixed to a rigid metal post and were free to make natural eye movements. Stimuli were displayed on a 4×3 feet back-projection screen at a distance of 30 cm from the head, covering 130×100 degrees of visual angle. The screen resolution was 1024×768 pixels, with a refresh rate of 75 Hz.

Animals were presented with two stimulus conditions to which responses were analyzed here:

- Movie evoked (natural) activity: Stimuli consisted of a movie (the trailer for the film The Matrix), presented at a resolution of 720×480 pixels and a frame rate of 24 Hz. This stimulus ensemble is meant to capture the distribution of the statistics of natural stimuli at the level of the simple visual elements encoded by V1 neurons.
- Noise evoked (artificial) activity: Random noise was generated as a grid of black and white squares, each occupying 5×5 degrees of visual angle. A new pattern was generated at random at each screen refresh, with white squares appearing independently with probability 1/4.

Recordings were also performed in two other stimulus conditions, using drifting gratings and complete darkness (spontaneous activity), respectively. We reported analyses of these conditions elsewhere (Berkes et al., 2011), but they were not analyzed here as our theory of sequentiality did not apply to them: drifting gratings themselves were already sequential by construction, making it impossible to identify sequentiality in responses as spurious, and we had no control over the input reaching visual cortex in the spontaneous activity condition.

Recordings with different stimulus ensembles were performed in interleaved trials of 100 sec, 15 trials for each ensemble, for a total of 25 minutes of recording in each condition. Figure 5F presents an analysis using all 25 mins of each of movie-evoked (natural) and noise-evoked (artificial) recordings. Figures 5G and S5 present analyses using the first and second halves of these ‘concatenated’ 25 minutes of each condition.

#### Neural circuit model

Our model is composed of a set of *N* variables $v_{i}$, i=1,…,N, each variable describing the activation of a given neuron, e.g. the membrane potential. The membrane potential evolves in time according to a firing rate model, i.e. a set of *N* coupled first-order differential equations (see also Equation 1, repeated here for convenience):

(Equation 5) $$\tau \frac{dv_{i}}{dt}=-v_{i}\left(t\right)+\sum_{j=1}^{N}W_{ij}\;f\left[v_{j}\left(t\right)\right]+\xi_{i}\left(t\right)$$

where *W* is the synaptic matrix, f[⋅] is a function expressing the transformation of membrane potentials into momentary firing rates, $\xi_{i}$ is the external input and *τ* is the membrane time constant. Although network dynamics were purely rate-based (Equation 5), for illustrative purposes only, we also generated spikes based on neural firing rates in Figure 1C. Specifically, for each neuron *i*, action potentials were generated according to an inhomogeneous Poisson process with rate $f\left[v_{i}\left(t\right)\right]$. We also used a mean-field approach to simulate the average effects of STDP on synaptic weights (Figure 4), which again was only based on firing rates (see below, A model of spike timing-dependent plasticity). Similarly, all analyses of simulated data (e.g. covariances, sequentiality) were based on the firing rates, not the spikes. Thus, the spikes we generated did not enter into any further simulations or computations. Nevertheless, all our analyses using firing rates are equivalent to analyzing spiking activity over sufficiently long recordings in which the effects of (Poisson) spiking average out.

We assume that the external input $\xi_{i}\left(t\right)$ (the last term in Equation 5) is a stationary Gaussian stochastic process, with a mean *μ* (that is the same for all cells) and a “separable” covariance function that factorizes across space and time:

(Equation 6) $$C_{ij}^{\text{in}}\left(s\right)=\left\langle \delta \xi_{i}\left(t+s\right)\;\delta \xi_{j}\left(t\right)\right\rangle =\Sigma_{ij}^{\text{in}}g\left(s\right)$$

where angular brackets ⟨⋅⟩ denote averaging over different realizations of the stochastic process $\xi_{i}\left(t\right)$, and δ⋅ denotes deviations from mean. Equation 6 defines the matrix of input spatial covariances $\Sigma^{\text{in}}$, a positive definite matrix that does not depend on *s*, as well as the temporal covariance function g(s), a scalar and time-symmetric function obeying g(−s)=g(s). In all simulations we use

(Equation 7) $$g\left(s\right)=e^{-\left|s\right|/\tau_{\text{in}}}.$$

We generate Gaussian noise with such exponentially decaying correlations (Ornstein-Uhlenbeck process) by filtering white noise with a first-order low-pass filter with characteristic timescale $\tau_{\text{in}}$. The values of the synaptic matrix *W* and spatial covariance matrix $\Sigma^{\text{in}}$ vary across simulations, and are described in Parameter values along with the values of all other parameters. We simulate the temporal evolution of $v_{i}$ in Equation 5 using the Euler method with step size dt, initial conditions $v_{i}=0$, and a total number of time steps *T*.

#### Parameter values

In all simulations we set dt=1 ms, τ=20 ms, $\tau_{\text{in}}=15$ ms, $T=10^{5}$, and μ=0.1. In Figures 1 and 3, we use rectified linear units (ReLU) as response functions for all neurons: f[v]=max(0,v) (also clipped at one), whose slope is adjusted in order to have an average population output of 0.1. In Figure 2 we use a linear response function, f[v]=v. In Figures 1, 2F–2H, and 3, we set N=50, while N=2 in Figures 2A–2D.

In Figures 1 and 3A, the input covariance is equal to $\Sigma_{ij}^{\text{in}}=0.5\;s_{i}\;s_{j}$ for i≠j, and $\Sigma_{ii}^{\text{in}}=s_{i}^{2}$, where $s_{i}$ values are drawn independently from an exponential distribution with mean equal to one. This corresponds to an input with correlations fixed to 0.5 and random variances $s_{i}^{2}$ across neurons. In Figures 3B and 3C, the input covariance is equal to $\Sigma_{ij}^{\text{in}}=s_{i}\;s_{j}\;\text{exp}\left(-\left|i-j\right|/10\right)$. This corresponds to correlations that decay in space within a scale of 10 neurons, and again random variances $s_{i}^{2}$ across neurons. In Figure 2 the input covariance is equal to, respectively, $\Sigma^{\text{in}}=\left[25\;0;0\;1\right]$ in panel A, and $\Sigma^{\text{in}}=\left[1\;0.9;0.9\;1\right]$ in panels B and C. In Figure 2D we draw $\Sigma^{\text{in}}$ from a Wishart distribution with mean equal to the 2×2 identity matrix and 2 degrees of freedom. In Figures 2F and 2G we set $\Sigma^{\text{in}}$ as in Figure 1. In Figure 2H, we set it to the sum of (i) a uniform matrix with all elements set to the same *c* drawn uniformly between 0 and 0.2 (which determined the resulting fraction of negative covariances), and (ii) a random covariance matrix $\propto U\text{Λ}U^{T}$ where *U* is a random unitary matrix and Λ is a diagonal matrix of exponentially decaying eigenvalues ${\text{Λ}}_{ii}=\text{exp}\left(-i/10\right)$.

In Figure 1 we draw $W_{ij}$ independently from a Gaussian distribution with zero mean and variance $\alpha^{2}/N$, and then we set $W\to \left(W+W^{T}\right)/\left(2\sqrt{2}\right)$ in order to obtain a symmetric matrix with maximum eigenvalue ≃α=0.9. In Figure 2 we set, respectively, W=[00.5;0.50] in panel A, W=[−0.50;00.5] in B, W=[−0.50.4;−0.40.5] in C. In Figure 2D, the matrix *W* is drawn as for Figure 1. In Figures 2E and 3, we set $W_{ij}=\Sigma_{ij}^{\text{in}}D$, where $D_{j}$ are randomly drawn +1 or −1 with equal probability, and we rescale the matrix in order to have a maximum eigenvalue α=0.9. In Figure 2F, W is set as in Figure 2E, but the $p\;N^{2}$ connections corresponding to the smallest values of $\left|W_{ij}\right|$ are set to zero, where *p* is the sparseness. In Figure 2G, W is set as in Figure 2E, but the Hebb part of the matrix is drawn from an inverse Wishart distribution with mean equal to $\Sigma^{\text{in}}$ and a varying number of degrees of freedoms, simulating a varying degree of noise. In Figure S2A, we draw $W_{ij}$ independently from a Gaussian distribution with zero mean and variance $\alpha^{2}/N$, in order to obtain a random matrix with maximum real part of the eigenvalue ≃α=0.9. In Figure S2B, we follow the same procedure as in Figure S2A, and then we set $W\to \left(W-W^{T}\right)/2\sqrt{2}$ in order to obtain an anti-symmetric matrix. In Figure S2C, we follow the same procedure as in Figure S2A, and then we set $W_{ij}\to \left|W_{ij}\right|\cdot \text{sign}\left[\sum_{i}W_{ij}\right]$ in order to obtain a Dale matrix.

In STDP experiments (Figure 4), we do not simulate the network dynamics (Equation 5) explicitly, and instead compute sequentiality from the weight matrix (and other parameters) analytically using the expressions in Equations S74, S77, and S78 (assuming linear network dynamics). For tracking changes in synaptic weights, we simulate the mean field dynamics of Equation S82, with the kernel of Equation S80. We use the following values of parameters for the kernel: $\tau_{+}=\tau_{-}=15$ ms, $a_{+}=0.4375/\tau_{+}$, $a_{-}=0.25/\tau_{-}$. The timescale for the STDP dynamics of Equation S82 is set to $\tau_{s}=10$ minutes. The integral in Equation S82 is computed assuming linear dynamics in the colored input case, as derived in Equation S90. In Figure 4B, the input covariance and initial synaptic matrix are the same as of Figure 1, while in Figure 4C the input covariance is the same as in Figure 3B, and the initial synaptic matrix is equal to the end result of Figure 4C. The Dale matrix *D* is fixed in all simulations as explained above.

### Quantification and statistical analysis

#### Cross-covariance

The cross-covariance is calculated for either the input or output signal, $X_{j,t}$, relevant for a neural population. In Figures 1, 2, and 3 these are the inputs received by individual simulated neurons, $\xi_{j}\left(t\right)$, and their activities, $v_{j}\left(t\right)$ (Equation 1), respectively. In Figure 5, these are the pixels of the stimulus and the spiking responses of the experimentally recorded units, with action potentials smoothed using an exponential kernel of 10 ms width. (Methods for the acquisition of neural recordings are described in experimental model and subject details). In Figures 1, 3, and 5, the cross-covariance is computed using the standard formula

(Equation 8) $$C_{jk}\left(s\right)=\frac{1}{T}\;\sum_{t}\left(X_{j,t+s}-\mu_{j}\right)\;\left(X_{k,t}-\mu_{k}\right)$$

(Equation 9) $$\mu_{j}=\frac{1}{T}\;\sum_{t}X_{j,t}$$

where $X_{j,t}$ is the value of signal *j* at time *t*, $\mu_{j}$ is the mean of $X_{j,t}$ over time, and *s* is the time lag. The cross-covariance is calculated for all $N^{2}$ pairs of signals, and for a set of *S* uniformly spaced time lags ranging from s=−200 ms to s=+200 ms in 2 ms bins. The index *t* runs over all allowed time steps, typically the length of the time series minus the number of time lags. Each dataset (either a numerical simulation or a recording session) is divided into M=10 epochs; cross-covariances are computed in each epoch and then averaged across epochs. This procedure reduced the effects of non-stationarities and enabled estimation of the noise floor in the singular values of the matrix *C* (see noise estimation).

In Figure 2, cross-covariances are computed using theoretical predictions of the linearized system, Equations S64–S68. We have always found a very good agreement between theoretical predictions and numerical simulations.

#### Sequentiality

Sequentiality is defined as the ratio of the Frobenius norm of $C^{\text{asy}}$ and that of $C^{\text{sym}}$, where $C^{\text{asy}}$ and $C^{\text{sym}}$ are the anti-symmetric and symmetric parts of the cross-covariance matrix *C*, respectively:

(Equation 10) $$\text{seq}=\frac{{\left|\left|C^{\text{asy}}\right|\right|}_{F}}{{\left\|C^{\text{sym}}\right\|}_{F}}=\sqrt{\frac{\sum_{s,j,k}{\left[C_{jk}\left(s\right)-C_{jk}\left(-s\right)\right]}^{2}}{\sum_{s,j,k}{\left[C_{jk}\left(s\right)+C_{jk}\left(-s\right)\right]}^{2}}}$$

Sequentiality is non-negative, because it is the ratio of non-negative numbers. Moreover, it is clear from Equation 10 that sequentiality is equal to zero if, and only if, the cross-covariance is symmetric for all neuron pairs, i.e. $C_{jk}\left(s\right)=C_{jk}\left(-s\right)$. This corresponds to a time series in which the second-order statistics is exactly time-reversible. Finally, we prove in Math Note S1 that sequentiality never exceeds one (Theorem 1). It is possible to show that seq = 1 in the case of a perfect sequence, defined by

(Equation 11) $$X_{j,t}=f_{\left(t-j\right)\text{ mod }N}$$

where $f_{t}$ is an arbitrary function of time *t*, which is displaced by one time step from one neuron to the next with cyclic boundary conditions (Figure S1).

Since data is noisy, we designed a method for estimating sequentiality, by separating the symmetric, anti-symmetric and noise terms of the cross covariance. We first define the matrix Γ, equal to $C_{ij}\left(s\right)$ where each column contains the cross-covariances of all possible pairs of neurons for a specific time lag (e.g. Figures 1F and 1G):

(Equation 12) $${\text{Γ}}_{\left(i+jN\right),s}=C_{ij}\left(s\right)$$

Thus, Γ is a matrix with $N^{2}$ rows and *S* columns, where each row corresponds to a given pair of neurons and each column corresponds to a given time lag. We prove in Theorem 2 that all right singular vectors of Γ (which have the interpretation of temporal components) are either symmetric or anti-symmetric in the lag *s*. Moreover, from the definition of the Frobenius norm, we have that

(Equation 13) $$\text{seq}=\sqrt{\frac{\sum_{m\in \text{asy}}\sigma_{m}^{2}}{\sum_{m\in \text{sym}}\sigma_{m}^{2}}}$$

where the index *m* runs over either the anti-symmetric (asy) or the symmetric (sym) singular vectors of Γ, associated with their corresponding singular values $\sigma_{m}$. Noise can be eliminated by dropping all singular values smaller than a given threshold, obtained by estimating the singular values of the noise (see below).

#### Noise estimation

We consider the problem of estimating the error made when measuring the singular values of a matrix (Horn and Johnson, 2012) estimated from noisy data. Let Γ denote our stochastic matrix estimate, composed of a signal part ${\text{Γ}}_{\text{s}}$ and a noise part ${\text{Γ}}_{\text{n}}$

(Equation 14) $$\text{Γ}={\text{Γ}}_{\text{s}}+{\text{Γ}}_{\text{n}}$$

We assume that ${\text{Γ}}_{\text{s}}$ is fixed (ground truth), and the noise term ${\text{Γ}}_{\text{n}}$ is normally distributed with zero mean and an arbitrary covariance matrix Σ, expressing covariances in terms of the vectorized matrix $\text{vec}\left({\text{Γ}}_{\text{n}}\right)$. The distribution of singular values of ${\text{Γ}}_{\text{n}}$ has been calculated analytically under some assumptions, e.g. when the covariance Σ has separable contributions for the rows and columns of ${\text{Γ}}_{\text{n}}$ (see case (ii) in SenGupta and Mitra, 1999). For arbitrary covariance matrices, this distribution can be estimated from the data by resampling. We assume that the estimate Γ is obtained from *M* independent and identically distributed samples indexed by *i*, namely

(Equation 15) $$\text{Γ}=\frac{1}{M}\sum_{i=1}^{M}{\text{Γ}}^{\left(i\right)}$$

For example, the samples ${\text{Γ}}^{\left(i\right)}$ can be obtained by dividing the data into *M* equal epochs. These samples can then be used to estimate the noise. In particular, we denote by ${\text{Γ}}_{\text{n}}^{\left(i\right)}$ the estimation error associated with sample *i*:

(Equation 16) $${\text{Γ}}^{\left(i\right)}={\text{Γ}}_{\text{s}}+{\text{Γ}}_{\text{n}}^{\left(i\right)}$$

The error in our overall estimate is simply the mean of all these error terms:

(Equation 17) $${\text{Γ}}_{\text{n}}=\frac{1}{M}\sum_{i=1}^{M}{\text{Γ}}_{\text{n}}^{\left(i\right)}$$

Assuming that each ${\text{Γ}}_{\text{n}}^{\left(i\right)}$ is also normally, independently and identically distributed with zero mean, then its covariance is equal to $\sqrt{M}\;\text{Σ}$. Therefore, the distribution of the singular values of ${\text{Γ}}_{\text{n}}$ is equal to the distribution of the singular values of ${\text{Γ}}_{\text{n}}^{\left(i\right)}/\sqrt{M}$:

(Equation 18) $$\sigma \left({\text{Γ}}_{\text{n}}\right)\;∼\;\sigma \left({\text{Γ}}_{\text{n}}^{\left(i\right)}/\sqrt{M}\right)=\sigma \left({\text{Γ}}_{\text{n}}^{\left(i\right)}\right)/\sqrt{M}$$

Noise samples are not directly available in data, but can be obtained by taking the difference of pairs of epochs (such that the common signal part cancels out), and rescaling by the appropriate factor:

(Equation 19) $$\text{Δ}{\text{Γ}}_{ij}={\text{Γ}}^{\left(i\right)}-{\text{Γ}}^{\left(j\right)}={\text{Γ}}_{\text{n}}^{\left(i\right)}-{\text{Γ}}_{\text{n}}^{\left(j\right)}\;\;\sigma \left({\text{Γ}}_{n}\right)\;∼\;\sigma \left(\text{Δ}{\text{Γ}}_{ij}\right)/\sqrt{2M}$$

Note that pairwise differences may be correlated, for example when using a single epoch of data to compute multiple pairs, but all possible N(N−1)/2 differences are expected to be distributed equally, and can all be used to estimate the mean and covariance of $\sigma \left({\text{Γ}}_{\text{n}}\right)$. In Figures 1, 3, and 5 we use this method to compute the mean *μ* and standard deviation *σ* of the spectrum of singular values of the noise, and subsequently discard all singular values of Γ that fall below μ+7σ.

#### Data analysis and exclusion

As our measure of sequentiality is based on a ratio taking values between zero and one, we reported its logarithm in all analyses based on experimental data (Figures 5 and S5), and for statistical testing using (two-tailed paired) t-tests. For the logarithm to be well defined, we excluded from these analyses all animals whose raw sequentiality was zero in either condition due to limited sample size. When analyzing all of the data (Figure 5F), one animal had zero sequentiality in the natural condition, and the same animal and an additional animal had zero sequentiality in the artificial condition, leaving 11 animals in the analysis. (We confirmed that the results remained unchanged when performing a Wilcoxon signed-rank test on the raw sequentiality scores without excluding any of the animals, p = 0.0122.) When comparing two halves of the data in the artificial condition (Figure 5G), the same four animals had zero sequentiality in both halves, leaving 9 animals in the analysis. When comparing two halves of the data in the natural condition (Figure S5), three animals had zero sequentiality in each half, with an overlap of only one animal, leading to the exclusion of 5 animals, leaving 8 animals in the analysis.

## Data Availability

• All original data has been deposited at https://doi.org/10.5281/zenodo.6124683 and is publicly available as of the date of publication. The DOI is listed in the key resources table. • All original code has been deposited at https://doi.org/10.5281/zenodo.6124314 and is publicly available as of the date of publication. The DOI is listed in the key resources table. • Any additional information required to reanalyze the data reported in this paper is available from the lead contact upon request.

## Supplemental information

Document S1. Notes S1–S3 and Figures S1–S5

Document S2. Article plus supplemental information
