# Supplementary material for: Adaptive erasure of spurious sequences in sensory cortical circuits
Source: Neuron. 2022 Jun 1;110(11):1857–1868.e5. doi: 10.1016/j.neuron.2022.03.006 (PMC9616807; doi:10.1016/j.neuron.2022.03.006)
Supplement: Document S1. Notes S1–S3 and Figures S1–S5 [file mmc1.pdf]

Neuron, Volume *110*

Supplemental information

Adaptive erasure of spurious sequences in sensory cortical circuits

Alberto Bernacchia, József Fiser, Guillaume Hennequin, and Máté Lengyel

Adaptive erasure of spurious sequences in cortical circuits SUPPLEMENTARY MATERIAL

Alberto Bernacchia[1,2*], József Fiser[3], Guillaume Hennequin[1,4], Máté Lengyel[1,3,4]

[1]Department of Engineering, University of Cambridge,
Cambridge CB2 1PZ, United Kingdom
[2]MediaTek Research, Building 2010, Cambourne Business Park
Cambourne CB23 6DW, United Kingdom
[3]Department of Cognitive Science, Central European University,
Budapest H-1051, Hungary
[4]Equal contribution.

[*]To whom correspondence should be addressed; E-mail: ab2349@cam.ac.uk.

Contents

Supplementary Math Note 1 Theory of sequentiality **1**
1.1 Bounds 1
1.2 Symmetries 3

Supplementary Math Note 2 Theory of time-reversible systems **3**
2.1 Summary of results 3
2.2 Derivation of covariance functions 4
2.3 Conditions for time reversibility 5
2.4 Linear examples 8

Supplementary Math Note 3 A model of spike timing-dependent plasticity **10**
3.1 Mean field dynamics 10
3.2 Example 1: linear dynamics and white input 11
3.3 Example 2: linear dynamics and colored input 12

Supplementary Math Note 1 Theory of sequentiality

1.1 Bounds

Theorem 1. *Sequentiality is bounded between zero and one.*

Proof. Sequentiality is trivially non-negative, because it is the ratio of non-negative numbers, therefore we only have to prove that it is smaller than one. We will assume that the cross-covariance is computed for all possible values of time lag ($S = T$) and wrapped around in a circle (i.e. $C(T + s) = C(s)$). In particular, we assume that T is odd and consider time

lags between $-(T-1)/2$ and $(T-1)/2$. In practice, this assumption is inessential as long as the maximum time lag is large enough to cover the bulk of the cross-covariance.

In matrix form, sequentiality in Equation 10 can be rewritten as

$$\text{seq}^2 = \frac{\sum_s \text{Tr}\left\{[C(s) - C(-s)]\,[C(s) - C(-s)]^T\right\}}{\sum_s \text{Tr}\left\{[C(s) + C(-s)]\,[C(s) + C(-s)]^T\right\}} \tag{S1}$$

Using the property $C(-s) = C(s)^T$, this can be simplified to

$$\text{seq}^2 = \frac{\sum_s \text{Tr}\left[C(s)C(s)^T\right] - \sum_s \text{Tr}\left[C(s)^2\right]}{\sum_s \text{Tr}\left[C(s)C(s)^T\right] + \sum_s \text{Tr}\left[C(s)^2\right]} \tag{S2}$$

In order to prove that sequentiality never exceeds one, we have to prove that the numerator never exceeds the denominator. By inspection of Equation S2, this is true if and only if $\sum_s \text{Tr}\left[C(s)^2\right] \geq 0$. Note that $\text{Tr}\left[C(s)C(s)^T\right]$ is non-negative for all values of s since it is equal to the sum of squared elements of $C(s)$. However, the second term, $\text{Tr}\left[C(s)^2\right]$ may be negative in some cases. For example, if $C(s)$ is anti-symmetric for some values of s, then $\text{Tr}\left[C(s)^2\right]$ is negative. However, we show below that it is non-negative when summed over all values of s.

We define the cross spectral matrix as the discrete Fourier transform of the cross covariance

$$\hat{C}(\omega) = \frac{1}{\sqrt{T}} \sum_s e^{-2\pi i s\omega/T} C(s) \tag{S3}$$

Since $C(s)^T = C(-s)$, it is straightforward to show that the cross spectral matrix is Hermitian. It is also possible to show that $\hat{C}(\omega)$ is positive semi-definite, for all frequencies ω. We define the matrix $\Pi_{tt'} = \mathbb{E}[x_t x_{t'}] = C(t-t')$, for a scalar and stationary random process x, where $\mathbb{E}$ denotes expectation over its distribution. The matrix Π is positive semidefinite and circulant, and Fourier transforming along both time variables (t and t') we obtain $\hat{\Pi}_{\omega\omega'} = \delta_{\omega\omega'}\hat{C}(\omega)$. Since $\hat{\Pi}$ is positive semidefinite, $\hat{C}(\omega)$ must be non-negative. The extension to the multivariate case is straightforward, and shows that $\hat{C}(\omega)$ is positive semi-definite. Using Parseval's theorem, we have

$$\sum_s \text{Tr}\left[C(s)^2\right] = \sum_\omega \text{Tr}\left[\hat{C}(\omega)\overline{\hat{C}(\omega)}\right] \tag{S4}$$

Each element of the sum on the right hand side must be non-negative, because it is the trace of a product of two Hermitian positive semi-definite matrices (see Theorem 4.3.53 in Ref. 80). Using similar arguments, we find

$$\sum_s \text{Tr}\left[C(s)C(s)^T\right] = \sum_\omega \text{Tr}\left[\hat{C}(\omega)^2\right] \tag{S5}$$

Therefore, substituting these expressions into Equation S2, we find that sequentiality is equal to

$$\text{seq}^2 = \frac{\sum_\omega \text{Tr}\left[\hat{C}(\omega)^2\right] - \sum_\omega \text{Tr}\left[\hat{C}(\omega)\overline{\hat{C}(\omega)}\right]}{\sum_\omega \text{Tr}\left[\hat{C}(\omega)^2\right] + \sum_\omega \text{Tr}\left[\hat{C}(\omega)\overline{\hat{C}(\omega)}\right]} \tag{S6}$$

which never exceeds one since all sums are positive.

$\square$

1.2 Symmetries

Theorem 2. *When its singular values are distinct, all singular vectors of the reshaped cross-covariance* Γ *in Equation 12 are either symmetric or anti-symmetric.*

Proof. Let K be the commutation matrix, of size $N^2 \times N^2$, operating in the vector space of neuron pairs, that replaces the $(i + jN)$ pair with the $(j + iN)$ pair. This corresponds to transposing the original cross covariance matrix, replacing ij with ji, but operating on the vector space of neuron pairs. We also define R as the reflection matrix that replaces the time lag s with the time lag $-s$. This corresponds to flipping the time lag vector. Using the identity $C_{ij}(s) = C_{ji}(-s)$, it is straightforward to note that transposing space or transposing time are two equivalent operations, i.e.

$$K\Gamma R^T = \Gamma \tag{S7}$$

Therefore the reshaped cross-covariance matrix is invariant upon the simultaneous action of K and R. We denote the SVD of Γ by

$$\Gamma = USV^T \tag{S8}$$

where S is a diagonal matrix containing the singular values of Γ, and the columns of matrix U (resp. V) are the (orthogonal) left (resp. right) singular vectors of Γ. Then, Equations S7 and S8 imply that

$$(KU)S(RV)^T = USV^T \tag{S9}$$

Since the permutation matrices K and R are orthogonal, the matrices in round brackets are also orthogonal. Note that the SVD of a matrix is unique, provided that singular values are distinct, and except for a simultaneous sign change of the same column of U and V. Therefore, we must have that

$$KU = U\pm \tag{S10}$$

$$RV = V\pm \tag{S11}$$

where $\pm$ is a diagonal matrix composed of $+1$ and -1 elements only. Note that signs apply separately to different columns, but the sign of a given column of U and V matches. From the meaning of the K and R as spatial transpose and time-reversal respectively, these equations imply that each pair singular vectors (left and right) are either both symmetric or both anti-symmetric in space (left) and time (right). This, in turn, means that each singular value can be unequivocally labelled as "symmetric" or "anti-symmetric", thus justifying the alternative expression of sequentiality given by Equation 13. □

Supplementary Math Note 2 Theory of time-reversible systems

2.1 Summary of results

We are interested in determining under which conditions the activity produced by the dynamical system in Equation 5 is time-reversible. Our main assumptions are: 1) that the system relaxes to a stationary state, and 2) that all $v_i(t)$ and $\xi_j(t)$ are jointly Gaussian. These assumptions are exactly satisfied only when $f_j[v] \propto v$ (linear response functions), but they still hold approximately for weakly nonlinear transfer functions, especially if N is large. We show in Section 2.3 that the following synaptic matrix guarantees (is a sufficient condition for) time reversibility:

$$W_{ij} = H_{ij}D_j \tag{S12}$$

where H — which we refer to as the "Hebb" part — is a matrix that must satisfy the conditions detailed in Section 2.3 and D — the "Dale" part (see below) — is some vector of presynaptic factors. A simple example of a "Hebb" factor H that guarantees time reversibility is the input covariance

$$H_{ij} = \Sigma^{\text{in}}_{ij} \tag{S13}$$

Another possible form is

$$H_{ij} = \int_{-\infty}^{+\infty} ds\, C_{ij}^{\text{out}}(s)k(s) \tag{S14}$$

where $k(s)$ is an arbitrary scalar kernel function and Σ^{out} is the output covariance matrix,

$$C_{ij}^{\text{out}}(s) = \langle \delta v_i(t+s)\delta v_j(t)\rangle \tag{S15}$$

Note that C^{out} depends on H itself, therefore Equation S14 gives an implicit formula that must be solved for H. The proportionality of synaptic strengths to the covariance of neural activity is consistent with empirical observations Cossell et al. (2015). Furthermore, by interpreting $k(s)$ as an STDP kernel, we show in Section 3.1 that this matrix represents a fixed point of STDP dynamics, which can be simulated in order to solve Equation S14. Finally, assuming that all elements of the Hebb component H are positive, then neuron j is excitatory (i.e. all its outgoing weights are positive) if $D_j \geq 0$; it is inhibitory otherwise. This justifies the interpretation of D as a "Dale" component. The derivation of Equations S12 to S14 is provided in Section 2.3 (see derivations leading up to Equations S47 and S54).

2.2 Derivation of covariance functions

In this section we derive formulas for the covariance functions, under the assumptions of stationarity and Gaussianity. For convenience of notation, we rewrite Equation 5 in matrix form as

$$\tau \frac{dv}{dt} = -v(t) + W\, f\,[v(t)] + \xi(t) \tag{S16}$$

We assume that the system relaxes to a stationary state, and define the following cross-covariance matrices

$$C^{\text{in}}(s) = \left\langle \delta\xi(t+s)\delta\xi(t)^T \right\rangle \tag{S17}$$

$$C^{\text{out}}(s) = \left\langle \delta v(t+s)\delta v(t)^T \right\rangle \tag{S18}$$

$$C^{\text{io}}(s) = \left\langle \delta v(t+s)\delta\xi(t)^T \right\rangle \tag{S19}$$

where angular brackets correspond to the operation of averaging over different realizations of the stochastic process $\xi(t)$. Note that stationarity implies that $C^{\text{in}}(-s)^T = C^{\text{in}}(s)$, and $C^{\text{out}}(-s)^T = C^{\text{out}}(s)$. The following theorem provides an expression for these cross-covariance matrices.

Theorem 3. *Given the dynamical system described by Equation S16, if the system is stationary and the variables (v, ξ) are jointly Gaussian, then the cross-covariance matrices satisfy the following expressions:*

$$(i\omega\tau - J)\,\hat{C}^{\text{io}}(\omega) = \hat{C}^{\text{in}}(\omega) \tag{S20}$$

$$(i\omega\tau - J)\,\hat{C}^{\text{out}}(\omega) = \hat{C}^{\text{io}}(-\omega)^T \tag{S21}$$

where

$$J = W\Phi - I \tag{S22}$$

with a diagonal matrix Φ with elements

$$\Phi_{ii} = \langle f_i'\,[v_i]\rangle\,. \tag{S23}$$

Here, the Fourier transform and inverse transform are defined by

$$\hat{C}(\omega) = \frac{1}{\sqrt{2\pi}} \int_{-\infty}^{+\infty} ds\, C(s)e^{-i\omega s} \tag{S24}$$

$$C(s) = \frac{1}{\sqrt{2\pi}} \int_{-\infty}^{+\infty} d\omega\, \hat{C}(\omega)e^{i\omega s}. \tag{S25}$$

Proof. We first derive the differential equations that govern the evolution of the covariance matrices. To do this, we shift temporally and multiply Equation S16 by, respectively, the transposed input and output deviations

$$\tau \frac{dv(t+s)}{ds} \delta\xi(t)^T = -v(t+s)\delta\xi(t)^T + W f[v(t+s)]\,\delta\xi(t)^T + \xi(t+s)\delta\xi(t)^T \tag{S26}$$

$$\tau \frac{dv(t+s)}{ds} \delta v(t)^T = -v(t+s)\delta v(t)^T + W f[v(t+s)]\,\delta v(t)^T + \xi(t+s)\delta v(t)^T \tag{S27}$$

In order to average these equations, we use the assumption that $\{\xi(t), v(s)\}$ are jointly distributed according to a multivariate Gaussian, along with Bussgang's theorem, to obtain[1]

$$\left\langle f[v(t+s)]\,\delta\xi(t)^T \right\rangle = \Phi \left\langle \delta v(t+s)\delta\xi(t)^T \right\rangle = \Phi C^{\text{io}}(s) \tag{S28}$$

$$\left\langle f[v(t+s)]\,\delta v(t)^T \right\rangle = \Phi \left\langle \delta v(t+s)\delta v(t)^T \right\rangle = \Phi C^{\text{out}}(s) \tag{S29}$$

where the diagonal matrix Φ is given in the theorem statement. Note that $\Phi = I$ when the transfer function is linear. Defining the matrix $J = W\Phi - 1$, and averaging Equations S26 and S27, we obtain the following differential equations for the covariance matrices:

$$\tau \frac{d}{ds} C^{\text{io}}(s) = JC^{\text{io}}(s) + C^{\text{in}}(s) \tag{S30}$$

$$\tau \frac{d}{ds} C^{\text{out}}(s) = JC^{\text{out}}(s) + C^{\text{io}}(-s)^T \tag{S31}$$

The theorem follows by applying the Fourier transform to Equations S30 and S31. □

Concerning implementation, we first note that $\hat{C}^{\text{in}}(-\omega)^T = \hat{C}^{\text{in}}(\omega)$, and $\hat{C}^{\text{out}}(-\omega)^T = \hat{C}^{\text{out}}(\omega)$; however, in general $\hat{C}^{\text{io}}(-\omega) \neq \hat{C}^{\text{io}}(\omega)$. Furthermore, $\hat{C}^{\text{io}}(\omega)$ can be eliminated from Equations S20 and S21, to obtain

$$(i\omega\tau - J)\,\hat{C}^{\text{out}}(\omega)\left(-i\omega\tau - J^T\right) = \hat{C}^{\text{in}}(\omega) \tag{S32}$$

This equation is nonlinear, since the matrix Φ, and therefore J, depends on the diagonal of $C^{\text{out}}(0)$. However, J does not depend on ω; thus, we can alternate between (i) solving a *linear* equation for $\hat{C}^{\text{out}}$ given fixed J, and (ii) fixing $\hat{C}^{\text{out}}$ and using Equation S22 to obtain J. This will yield a self-consistent solution.

2.3 Conditions for time reversibility

In this section, we turn to the problem of finding necessary and sufficient conditions for time reversibility. Recall that we assumed that the input covariance is symmetric in time (cf. Equation 6 and below):

$$C^{\text{in}}(-s) = C^{\text{in}}(s) \tag{S33}$$

By definition, the network output is said to be time-reversible if the same type of equality holds for network activity as well:

$$C^{\text{out}}(-s) = C^{\text{out}}(s) \tag{S34}$$

Therefore, the goal is to find conditions under which Equation S33 implies Equation S34. We stress that time reversibility implies that the joint probability of two states, at two different time points, is symmetric. Because of the Gaussianity assumption, this reduces to time reversibility of the covariance, Equation S34, since higher order statistics do not contribute (and the mean is constant due to stationarity). We prove the following.

[1] For a Gaussian distribution $P(\boldsymbol{x})$ of zero mean and covariance Σ, the following identity holds: $\Sigma\boldsymbol{\nabla}P = -\boldsymbol{x}P$, therefore $\langle f(x_1)x_2\rangle = \int dx_1 \int dx_2 f(x_1)x_2 P(x_1,x_2) = -\int dx_1 \int dx_2 f(x_1)\left(\Sigma_{21}\partial_{x_1} + \Sigma_{22}\partial_{x_2}\right)P(x_1,x_2) = -\Sigma_{21}\int dx_1 f(x_1)\partial_{x_1}P(x_1) - \Sigma_{22}\int dx_1 f(x_1)\int dx_2 \partial_{x_2}P(x_1,x_2)$. Integrating by parts, the second term is zero while the first is equal to $\Sigma_{21}\langle f'(x_1)\rangle$.

Theorem 4. *Given time-reversible input, the output is time reversible if and only if any one of the following equalities holds:*

$$JC^{\text{out}}(s) = C^{\text{out}}(s)J^T \qquad \textit{for all } s \tag{S35}$$
$$JC^{\text{io}}(s) = C^{\text{io}}(s)J^T \qquad \textit{for all } s \tag{S36}$$
$$JC^{\text{in}}(s) = C^{\text{in}}(s)J^T \qquad \textit{for all } s \tag{S37}$$

Proof. Due to the stationarity assumption, symmetry in time implies symmetry in space, since as noted above we must have that $C^{\text{in}}(-s)^T = C^{\text{in}}(s)$, which is equivalent to $\hat{C}^{\text{in}}(-\omega)^T = \hat{C}^{\text{in}}(\omega)$ in Fourier space. Therefore, the input covariance satisfies the following equalities:

$$C^{\text{in}}(s) = C^{\text{in}}(s)^T \tag{S38}$$
$$\hat{C}^{\text{in}}(\omega) = \hat{C}^{\text{in}}(\omega)^T \tag{S39}$$

Similarly, the output is time reversible if and only if either of the following equalities holds:

$$C^{\text{out}}(s) = C^{\text{out}}(s)^T \tag{S40}$$
$$\hat{C}^{\text{out}}(\omega) = \hat{C}^{\text{out}}(\omega)^T \tag{S41}$$

We focus on proving $\hat{C}^{\text{out}}(\omega)^T = \hat{C}^{\text{out}}(\omega)$. For brevity, we omit the dependence on ω, and we denote by $\hat{C}^{\text{out}}_{\text{sym}} = \hat{C}^{\text{out}} + \hat{C}^{outT}$ the symmetric part of the covariance, and by $\hat{C}^{\text{out}}_{\text{asy}} = \hat{C}^{\text{out}} - \hat{C}^{\text{out}T}$ its anti-symmetric part. Using Equations S32 and S33, we have that

$$i\omega\tau\left(J\hat{C}^{\text{out}}_{\text{sym}} - \hat{C}^{\text{out}}_{\text{sym}}J^T\right) + \omega^2\tau^2\hat{C}^{\text{out}}_{\text{asy}} + J\hat{C}^{\text{out}}_{\text{asy}}J^T = 0 \tag{S42}$$

Since this equation is linear in the covariance, the anti-symmetric part is zero if, and only if, the term in round brackets is zero. Therefore, a necessary and sufficient condition for time reversibility is

$$J\hat{C}^{\text{out}}(\omega) = \hat{C}^{\text{out}}(\omega)J^T \quad \text{for all } \omega \tag{S43}$$

Furthermore, it is straightforward to show that if J satisfies this equation, then all its powers satisfy the same equation, and therefore so does any analytic function of J. The converse is also true, since the inverse of any analytic function is also analytic. In particular, $i\omega\tau - J$ is analytic in J, and so is its inverse. Thus, from Equations S20 and S21, we have that

$$J\hat{C}^{\text{io}}(\omega) = \hat{C}^{\text{io}}(\omega)J^T \quad \text{for all } \omega \tag{S44}$$
$$J\hat{C}^{\text{in}}(\omega) = \hat{C}^{\text{in}}(\omega)J^T \quad \text{for all } \omega \tag{S45}$$

Therefore, any one of the three Equations S43 to S45 implies, and is implied by, any one of the other two. The theorem follows by applying the inverse Fourier transform to Equations S43 to S45. □

Consequence of Theorem 4 for "Hebb and Dale" conditions. Each one of Equations S35 to S37, on its own, is necessary and sufficient for time-reversibility of the output. Since the input covariance is given, while the other covariances must be determined, it is easiest to test for reversibility by checking whether Equation S37 holds. Using the separability of the input covariance (Equation 6), Equation S37 is rewritten as

$$J\Sigma^{\text{in}} - \Sigma^{\text{in}}J^T = 0 \tag{S46}$$

Using $J = W\Phi - I$, and noting that Φ is diagonal, it is possible to show that

$$W = \Sigma^{\text{in}}D \tag{S47}$$

satisfies Equation S37 for any arbitrary diagonal matrix D. This corresponds to Equation S13 in Section 2.1. However, other forms are also possible, including Equation S14, and we show in the following theorem how to derive them. We also note that, in the special case of a linear transfer function ($\Phi = I$), Equation S46 can be rewritten as

$$W\Sigma^{\text{in}} - \Sigma^{\text{in}}W^T = 0 \tag{S48}$$

Since sequentiality is zero when this equation holds, we use the norm of the left hand side of this expression as an approximation for *asym* in Equation 4 of the main text for $N = 2$ and for symmetric W and Σ. We verified that this approximation was accurate over a wide range of parameters in Figure 2C (Star Methods, "Parameter values").

Theorem 5. *We assume that the matrix J has distinct eigenvalues and the matrix Σ is non-singular. If a matrix J exists satisfying the equation*

$$J\Sigma = \Sigma J^T \tag{S49}$$

then Σ must be equal to

$$\Sigma = VEV^T \tag{S50}$$

where the columns of V are the eigenvectors of J, and E is a diagonal matrix. (Note that V needs not be an orthogonal basis, i.e. $V^TV \neq I$ in general). If we further assume that Σ is positive definite, then J must be equal to

$$J = \Sigma L \tag{S51}$$

where L is a symmetric matrix.

Proof. To verify this statement, note that Equation S49 implies $J = \Sigma J^T \Sigma^{-1}$. By writing the spectral decomposition of $J = V\Delta V^{-1}$, where V and Δ are, respectively, the eigenvectors and eigenvalues of J, we have that $\Delta = \left(V^T\Sigma^{-1}V\right)^{-1} \Delta \left(V^T\Sigma^{-1}V\right)$. Since the eigenvalues of J are distinct, $V^T\Sigma^{-1}V$ must be diagonal. Call this diagonal matrix E^{-1}, this gives us Equation S50. Moreover, Sylvester's law of inertial states that VEV^T have the same number of positive and negative eigenvalues as E does. Therefore, if we further assume that Σ is positive definite, then E must have positive elements. Under this assumption, we consider the unique singular value decomposition of $VE^{1/2} = U\Lambda^{1/2}U'$, where Λ is the diagonal matrix of singular values and U, U' are the orthogonal matrices of left and right singular vectors, respectively. It is straightforward to show that U and Λ are, respectively, the eigenvectors and eigenvalues of $\Sigma = U\Lambda U^T$. Finally, we rewrite J by substituting the expression of its eigenvectors, $V = U\Lambda^{1/2}U'E^{-1/2}$, in its spectral decomposition, $J = V\Delta V^{-1}$, and find

$$J = U\Lambda^{1/2}U'E^{-1/2}\Delta E^{1/2}U'^T\Lambda^{-1/2}U^T = U\Lambda U^T \left[U\Lambda^{-1/2}U'\Delta U'^T\Lambda^{-1/2}U^T\right] = \Sigma L \tag{S52}$$

where the last equality defines L. If J is unknown, while Σ is given, then U and Λ are given, while U' and Δ are arbitrary. Therefore, $U'\Delta U'^T$ is an arbitrary symmetric matrix, and so is L. □

In particular, Equation S50 allows us to conclude that, if a matrix J exists for which the system is time reversible, and Equation S35 holds, then we must have that $C^{\text{out}}(s) = VE^{\text{out}}(s)V^T$, where V are the eigenvectors of J and $E^{\text{out}}(s)$ is some diagonal matrix. Furthermore, since Equation S35 holds for all values of s, then any linear combination of different values of s can be taken. By taking an integral over different s values, weighted by a kernel $k(s)$, we have that $J \int ds\ k(s)C^{\text{out}}(s) = \int ds\ k(s)C^{\text{out}}(s)\ J^T$. Provided that the integral is positive definite, Theorem 5 implies that the synaptic matrix J must be equal to

$$J = \int_{-\infty}^{+\infty} ds\ k(s)C^{\text{out}}(s)\ L \tag{S53}$$

for some arbitrary scalar kernel $k(s)$ and symmetric matrix L. Conversely, we can set J by choosing any arbitrary symmetric matrix L in this expression, and this would still guarantee time reversibility. It is straightforward to show that L can be chosen in a way that

$$W = \int_{-\infty}^{+\infty} ds\ k(s)C^{\text{out}}(s)\ D \tag{S54}$$

where D is an arbitrary diagonal matrix. This corresponds to Equation S14 in Section 2.1. We will show in Section 3.1 that this expression can be interpreted as a fixed point of spike timing-dependent plasticity (STDP). As a final remark, we stress that no J may exist for which time reversibility holds (for example, for more complex types of input), in which case Equation S54 does not guarantee time reversibility.

2.4 Linear examples

In this section we consider the simple case in which the neural dynamics are linear, thus $\Phi = I$ and $J = W - I$ (see Section 2.2). In this case, the cross-covariances can be calculated analytically when the input has simple statistics.

First, we consider the case of white noise input, i.e. an input covariance of the form $C^{\text{in}}(s) = \Sigma^{\text{in}}\delta(s)$. We denote by $C^{\text{out}}(s)$ the output cross-covariance and by $\Sigma^{\text{out}} = C^{\text{out}}(0)$ the output covariance at zero time lag. The output covariance can be calculated from Equation S32, and is equal to

$$C^{\text{out}}(s) = \begin{cases} e^{(W-I)s/\tau}\ \Sigma^{\text{out}} & \text{for } s \geq 0 \\ \Sigma^{\text{out}}\ e^{-(W^T-I)s/\tau} & \text{for } s < 0 \end{cases} \tag{S55}$$

The covariance at zero time lag Σ^{out} is equal to

$$\Sigma^{\text{out}} = \frac{1}{\tau^2}\int_0^{+\infty} ds\ e^{(W-I)s/\tau}\ \Sigma^{\text{in}}\ e^{(W^T-I)s/\tau} \tag{S56}$$

Computing this integral is complicated, but Σ^{out} can be calculated instead by solving the Lyapunov equation

$$(W-I)\,\Sigma^{\text{out}} + \Sigma^{\text{out}}\left(W^T - I\right) + \Sigma^{\text{in}}/\tau = 0 \tag{S57}$$

We calculate the sequentiality using the definition, Equation 10, that we rewrite here substituting sums with integrals, since time is continuous in this case

$$\text{seq}^2 = \frac{\text{Tr}\int_{-\infty}^{+\infty} ds\ C^{\text{out}}(s)C^{\text{out}}(s)^T - \text{Tr}\int_{-\infty}^{+\infty} ds\ C^{\text{out}}(s)^2}{\text{Tr}\int_{-\infty}^{+\infty} ds\ C^{\text{out}}(s)C^{\text{out}}(s)^T + \text{Tr}\int_{-\infty}^{+\infty} ds\ C^{\text{out}}(s)^2} \tag{S58}$$

We substitute the expression for C^{out}, given by Equation S55, in the integrals and find that sequentiality can be expressed as

$$\text{seq}^2 = \frac{\text{Tr}\left(\mathcal{I}_1\right) - \text{Tr}\left(\mathcal{I}_2\Sigma^{\text{out}}\right)}{\text{Tr}\left(\mathcal{I}_1\right) + \text{Tr}\left(\mathcal{I}_2\Sigma^{\text{out}}\right)} \tag{S59}$$

Where the integrals $\mathcal{I}_1$ and $\mathcal{I}_2$ are equal to

$$\mathcal{I}_1 = \int_{-\infty}^{+\infty} ds\ e^{(W-I)s/\tau}\ {\Sigma^{\text{out}}}^2 e^{(W^T-I)s/\tau} \tag{S60}$$

$$\mathcal{I}_2 = \int_{-\infty}^{+\infty} ds\ e^{(W-I)s/\tau}\ \Sigma^{\text{out}} e^{(W-I)s/\tau} \tag{S61}$$

These integrals can be calculated as the solution of the following equations

$$\left(W-I\right)\mathcal{I}_1 + \mathcal{I}_1\left(W^T - I\right) + 2\tau{\Sigma^{\text{out}}}^2 = 0 \tag{S62}$$

$$\left(W-I\right)\mathcal{I}_2 + \mathcal{I}_2\left(W - I\right) + 2\tau\Sigma^{\text{out}} = 0 \tag{S63}$$

These can be solved by standard methods for Lyapunov equations.

Another interesting case, which we use in all simulations, is that of colored noise input, which we model as a multivariate Ornstein-Uhlenbeck (OU) process with a covariance that is separable in space and time. The derivations performed above can be generalized to handle such coloured inputs by defining auxiliary variables that independently integrate their

own white noise inputs and feed their outputs (which now have colored OU structure) to the network. Thus, the first block of N variables correspond to the neural network, interacting through the synaptic matrix W, and integrating the input from the auxiliary variables with a time constant τ. The second block of N variables correspond to the auxiliary variables, which integrate white noise of covariance B, with a time constant τ_{in}, and they interact through a mixing matrix Z. The (colored) auxiliary variables are then fed into the neural variables. The mixing matrix Z is set to zero in all simulations implemented here. Under these block ordering conventions, the extended system is described by the following Jacobian:

$$J_e = \begin{pmatrix} \frac{W-I}{\tau} & \frac{I}{\tau} \\ 0 & \frac{Z-I}{\tau_{\text{in}}} \end{pmatrix} \tag{S64}$$

The extended input covariance is equal to $\Sigma_e^{\text{in}}\delta(s)$, with

$$\Sigma_e^{\text{in}} = \begin{pmatrix} 0 & 0 \\ 0 & B \end{pmatrix} \tag{S65}$$

showing that only the second block (auxiliary variables) receives the white noise input. Analogously, the output covariance of the extended system is composed of four blocks:

$$C_e(s) = \begin{pmatrix} C^{\text{out}}(s) & C^{\text{io}}(s) \\ C^{\text{io}}(-s)^T & C^{\text{in}}(s) \end{pmatrix} \tag{S66}$$

Note that since the output of the second block is interpreted as the input to the neural network, we have denoted it by $C^{\text{in}}(s)$. Similarly, the cross-covariance of the two blocks has the same interpretation as the input-output covariance as above, and is therefore denoted by $C^{\text{io}}(s)$. Finally, the top-left block is the covariance of the network output, naturally denoted by $C^{\text{out}}(s)$.

Since the extended system is driven by white noise, the extended output covariance can be calculated analytically, as in Equation S55, and is equal to

$$C_e(s) = \begin{cases} e^{J_e s} C_e(0) & \text{for } s \geq 0 \\ C_e(0) e^{-J_e^T s} & \text{for } s < 0 \end{cases} \tag{S67}$$

where the covariance at zero time lag $C_e(0)$ follows the Lyapunov equation

$$J_e C_e(0) + C_e(0) J_e^T + \Sigma_e^{\text{in}}/\tau_{\text{in}}^2 = 0 \tag{S68}$$

By summing over the corresponding blocks, the first term of the Lyapunov equation $J_e C_e(0)$ is rewritten as

$$\begin{pmatrix} (W-I)\Sigma^{\text{out}}/\tau + {\Sigma^{\text{io}}}^T/\tau & (W-I)\Sigma^{\text{io}}/\tau + \Sigma^{\text{in}}/\tau \\ (Z-I){\Sigma^{\text{io}}}^T/\tau_{\text{in}} & (Z-I)\Sigma^{\text{in}}/\tau_{\text{in}} \end{pmatrix} \tag{S69}$$

where Σ^{out}, Σ^{in}, Σ^{io} are the respective cross-covariances at zero time lag. The following equations follow, one for each block of the Lyapunov equation:

$$(Z-I)\Sigma^{\text{in}} + \Sigma^{\text{in}}(Z^T - I) + B/\tau_{\text{in}} = 0 \tag{S70}$$

$$(W-I)\Sigma^{\text{io}}/\tau + \Sigma^{\text{io}}(Z^T - I)/\tau_{\text{in}} + \Sigma^{\text{in}}/\tau = 0 \tag{S71}$$

$$(W-I)\Sigma^{\text{out}} + \Sigma^{\text{out}}(W^T - I) + {\Sigma^{\text{io}}}^T + \Sigma^{\text{io}} = 0 \tag{S72}$$

These equations can be solved numerically in order to find Σ^{in}, Σ^{io}, Σ^{out}. The white noise limit corresponds to $Z = 0$ and $\tau_{\text{in}} \to 0$, for which we have $\Sigma^{\text{io}} = B/2\tau$. In this limit, we can recover the Lyapunov Equation S57.

Sequentiality in the colored noise case can be calculated following similar methods as in the white noise case. We start again from Eq.S58, and we note that we can express C^{out} as

$$C^{\text{out}} = P C_e(s) P^T \tag{S73}$$

where $C_e(s)$ is given by Equation S66 and P is a $N \times 2N$ matrix equal with two blocks, $(I0)$, the $N \times N$ identity matrix and the $N \times N$ matrix with all elements equal to zero. Then, substituting the expression for C^{out} in the integrals, we find that sequentiality can be expressed as

$$\text{seq}^2 = \frac{\text{Tr}\left(P\mathcal{I}'_1 P^T\right) - \text{Tr}\left(P\mathcal{I}'_2 C_e(0) P^T\right)}{\text{Tr}\left(P\mathcal{I}'_1 P^T\right) + \text{Tr}\left(P\mathcal{I}'_2 C_e(0) P^T\right)}, \tag{S74}$$

where the integrals $\mathcal{I}'_1$ and $\mathcal{I}'_2$ are equal to

$$\mathcal{I}'_1 = \int_{-\infty}^{+\infty} ds\; e^{J_e s} C_e(0) P^T P C_e(0) e^{J_e^T s} \tag{S75}$$

$$\mathcal{I}'_2 = \int_{-\infty}^{+\infty} ds\; e^{J_e s} C_e(0) P^T P e^{J_e s}. \tag{S76}$$

These integrals can be calculated as the solution of the following equations

$$J_e \mathcal{I}'_1 + \mathcal{I}'_1 J_e^T + 2C_e(0) P^T P C_e(0) = 0 \tag{S77}$$

$$J_e \mathcal{I}'_2 + \mathcal{I}'_2 J_e + 2C_e(0) P^T P = 0 \tag{S78}$$

These can be solved by standard methods for Lyapunov equations.

Supplementary Math Note 3 A model of spike timing-dependent plasticity

3.1 Mean field dynamics

We now consider the case in which the dynamics of the synaptic matrix follow spike-timing-dependent plasticity (STDP), and we show that a fixed point of this dynamics corresponds to a time reversible state (Equation S54). We use a variant of STDP that depends on presynaptic spikes and the subthreshold postsynaptic membrane potential (rather than postsynaptic spikes; Clopath et al., 2010, see also Figure 4A). According to this rule, (additive) changes to the synaptic weight between presynaptic neuron j and postsynaptic neuron i occur due to two kinds of contributions. First, at the time of its occurrence, each presynaptic spike causes LTD (a decrease in the synaptic weight) that is proportional to the low-pass (causally) filtered version of the postsynaptic membrane potential at that time. Second, each presynaptic spike also gives rise to an (exponentially decaying) eligibility trace, which continually causes LTP (an increase in the synaptic weight) that is proportional to the momentary product of the eligibility trace and the (unfiltered) postsynaptic membrane potential. Thus, the change in the synaptic weight between the two cells can be described by the following equation:

$$\tau_s \frac{dW_{ij}}{dt} = -W_{ij} + \int_0^{+\infty} dt'\; k(t')\; \delta v_i(t)\, Y_j(t-t') + \int_0^{+\infty} dt'\; k(-t')\; \delta v_i(t-t')\, Y_j(t) \tag{S79}$$

where τ_s is the time constant of synaptic changes, $\delta v_i(\cdot)$ is the (unfiltered) membrane potential of the postsynaptic cell (relative to its long-run time-average), $Y_j(\cdot)$ is the spike train of the presynaptic cell (represented as a sum of Dirac-delta functions), and $k(t)$ for $t \geq 0$ describes the eligibility trace corresponding to a single presynaptic spike, used for determining the magnitude of LTP (described by the first integral), while for $t < 0$ it is the (sign- and time-inverted) kernel with which the postsynaptic membrane potential is filtered, used for determining the magnitude of LTD (described by the second integral).

An example kernel often used in the literature is

$$k(t) = \begin{cases} a_+ \exp(-t/\tau_+) & \text{if } t \geq 0 \\ -a_- \exp(t/\tau_-) & \text{otherwise} \end{cases} \tag{S80}$$

Therefore, if the activity of postsynaptic neuron i is high within a time interval of about τ_+ *following* a presynaptic spike in neuron j, then the synaptic weight increases by an amount proportional to a_+. Conversely, if the activity of postsynaptic neuron i is high within a time interval of about τ_- *preceding* a presynaptic spike in neuron j, then the synaptic weight decreases by an amount proportional to a_-.

While STDP is naturally defined for spiking neurons, e.g. as given by Equation S79, following Kempter et al. (1999); Dayan and Abbott (2001), we use a mean-field approach and describe the average effects of STDP assuming that presynaptic spiking is described by an inhomogeneous Poisson process. Thus, we first average Equation S79 over the distribution of possible spike trains given the underlying presynaptic firing rate time course, and describe STDP dynamics as

$$\tau_s \frac{dW_{ij}}{dt} = -W_{ij} + \int_0^{+\infty} dt'\ k(t')\ \delta v_i(t) f_j\left[v_j(t-t')\right] + \int_0^{+\infty} dt'\ k(-t')\ \delta v_i(t-t') f_j\left[v_j(t)\right] \tag{S81}$$

Second, we also average Equation S81 over the realizations of firing rates and membrane potentials, given their stationary distributions (and in particular, cross-covariances; Equation S29). As a result, STDP dynamics reduces to, in matrix form,

$$\tau_s \frac{dW}{dt} = -W + \int_{-\infty}^{+\infty} ds\ k(s)\, C^{\text{out}}(s)\ \Phi \tag{S82}$$

where we used Bussgang theorem and Φ is the diagonal matrix of average gains (see Section 2.2 and Equation S23). Instead of Equation S82, we consider the following, more general form

$$\tau_s \frac{dW}{dt} = -W + \int_{-\infty}^{+\infty} ds\ k(s)\, C^{\text{out}}(s)\ D \tag{S83}$$

where D is an arbitrary diagonal matrix that effectively gives each presynaptic neuron its own learning rate. The fixed points of Equation S83 are given by

$$W = \int_{-\infty}^{+\infty} ds\ k(s)\, C^{\text{out}}(s)\ D \tag{S84}$$

Thus, the diagonal matrix D plays the role of the "Dale" factor in Equation S54, and indeed we choose it to have a mix of positive and negative elements. Note that a negative element in D for an inhibitory synapse means that the learning rate is $|D|$ but the effective STDP kernel has been sign-inverted relative to that of excitatory synapses. Also note that the covariance $C^{\text{out}}(s)$ depends on W through Equation S32. In order to search for fixed points, we can simulate Equation S83 numerically. Nevertheless, note that Equation S84 itself does not guarantee Hebb-and-Dale connectivity, which would require that weights only depend on the zero-time lag correlations, $C^{\text{out}}(0)$, while here $C^{\text{out}}(s)$ is integrated over time lags s.

Equation S84 corresponds to the condition of time reversibility, Equation S54, which holds for any kernel function $k(s)$, provided that the integral is positive definite. However, stability of the fixed point may depend on the specific choice of $k(s)$. In the following sections we show two cases in which fixed points are described by simple algebraic equations.

3.2 Example 1: linear dynamics and white input

Using the STDP kernel of Equation S80 and the covariance in Equation S55, the integral in Equation S83 can be calculated analytically. Then, synaptic dynamics is equal to

$$\tau_s \frac{dW}{dt} = -W + a_+\tau_+ \left[I + \frac{\tau_+}{\tau}(I-W)\right]^{-1} \Sigma^{\text{out}} D - a_-\tau_-\Sigma^{\text{out}} \left[I + \frac{\tau_-}{\tau}\left(I - W^T\right)\right]^{-1} D \tag{S85}$$

At the time reversible fixed point, Equation S49 holds, which also implies that $W\Sigma = \Sigma W^T$. Substituting this expression in Equation S57, we find that the output covariance has the following expression $\Sigma^{\text{out}} = (I-W)^{-1}\Sigma^{\text{in}}/2\tau$. We further assume that the timescale of potentiation and depression are equal, $\tau_+ = \tau_- = \tau_k$; Then, using Equation S49 in Equation S85 and setting the time derivative to zero, we find that at the time reversible fixed point the synaptic matrix satisfies

$$2W\left(I-W\right)\left[I+\frac{\tau_k}{\tau}\left(I-W\right)\right]=\frac{\tau_k}{\tau}\left(a_+-a_-\right)\Sigma^{\text{in}}D \tag{S86}$$

This equation can be solved numerically by noting that the eigenvectors of W are equal to the eigenvectors of $\Sigma^{\text{in}}D$, and by solving a third order equation for the eigenvalues of W.

3.3 Example 2: linear dynamics and colored input

Using the STDP kernel of Equation S80 and the covariance in Equation S67, the integral in Equation S83 can be calculated analytically and is equal to

$$\int_{-\infty}^{+\infty}ds\;k(s)C_e(s)=a_+\left(\frac{1}{\tau_+}-J_e\right)^{-1}C_e(0)-a_-C_e(0)\left(\frac{1}{\tau_-}-J_e^T\right)^{-1} \tag{S87}$$

Note that only the first block of this matrix needs to be computed in order to simulate synaptic dynamics. The matrix in round brackets can be expressed in block form as

$$\frac{1}{\tau_\pm}-J_e=\begin{pmatrix}\frac{1}{\tau_\pm}+\frac{I-W}{\tau} & -\frac{I}{\tau}\\ 0 & \frac{1}{\tau_\pm}+\frac{I-Z}{\tau_{\text{in}}}\end{pmatrix} \tag{S88}$$

The matrix block inverse is equal to

$$\left(\frac{1}{\tau_\pm}-J_e\right)^{-1}=\begin{pmatrix}\left(\frac{1}{\tau_\pm}+\frac{I-W}{\tau}\right)^{-1} & \frac{1}{\tau}\left(\frac{1}{\tau_\pm}+\frac{I-W}{\tau}\right)^{-1}\left(\frac{1}{\tau_\pm}+\frac{I-Z}{\tau_{\text{in}}}\right)^{-1}\\ 0 & \left(\frac{1}{\tau_\pm}+\frac{I-Z}{\tau_{\text{in}}}\right)^{-1}\end{pmatrix} \tag{S89}$$

Therefore, the first block of the integral in Equation S87 is equal to

$$\begin{aligned}\int_{-\infty}^{+\infty}ds\;k(s)C^{\text{out}}(s)=a_+\left(\frac{1}{\tau_+}+\frac{I-W}{\tau}\right)^{-1}\Sigma^{\text{out}}-a_-\Sigma^{\text{out}}\left(\frac{1}{\tau_-}+\frac{I-W^T}{\tau}\right)^{-1}+\\ +\frac{a_+}{\tau}\left(\frac{1}{\tau_+}+\frac{I-W}{\tau}\right)^{-1}\left(\frac{1}{\tau_+}+\frac{I-Z}{\tau_{\text{in}}}\right)^{-1}\Sigma^{\text{io}T}-\\ -\Sigma^{\text{io}}\frac{a_-}{\tau}\left(\frac{1}{\tau_-}+\frac{I-Z^T}{\tau_{\text{in}}}\right)^{-1}\left(\frac{1}{\tau_-}+\frac{I-W^T}{\tau}\right)^{-1}\end{aligned} \tag{S90}$$

This expression, along with Equations S70 to S72 and S83, can be used to simulate synaptic plasticity under colored noise input. Note that this expression reduces to the white noise case for $\tau_{\text{in}}\to 0$.

When time reversibility holds, separately for both the auxiliary variables and the neural network, the covariances can be calculated explicitly, and we have that

$$\Sigma^{\text{in}}=(I-Z)^{-1}\frac{B}{2\tau_{\text{in}}} \tag{S91}$$

$$\Sigma^{\text{io}}=\frac{1}{\tau}\left(\frac{I-W}{\tau}+\frac{I-Z}{\tau_{\text{in}}}\right)^{-1}\Sigma^{\text{in}}=\left(\frac{I-W}{\tau}+\frac{I-Z}{\tau_{\text{in}}}\right)^{-1}(I-Z)^{-1}\frac{B}{2\tau\tau_{\text{in}}} \tag{S92}$$

$$\Sigma^{\text{out}}=(I-W)^{-1}\Sigma^{\text{io}}=(I-W)^{-1}\left(\frac{I-W}{\tau}+\frac{I-Z}{\tau_{\text{in}}}\right)^{-1}(I-Z)^{-1}\frac{B}{2\tau\tau_{\text{in}}} \tag{S93}$$

Furthermore, I assume that $\tau_+=\tau_-=\tau_\pm$ and I define $k_0=a_+-a_-$. Under time reversibility, the integral is equal to

$$\int_{-\infty}^{+\infty} ds\; k(s) C^{\rm out}(s) = k_0 \left(\frac{1}{\tau_\pm} + \frac{I-W}{\tau} \right)^{-1} \Sigma^{\rm out} + \tag{S94}$$

$$+ \quad \frac{k_0}{\tau} \left(\frac{1}{\tau_\pm} + \frac{I-W}{\tau} \right)^{-1} \left(\frac{1}{\tau_\pm} + \frac{I-Z}{\tau_{\rm in}} \right)^{-1} \Sigma^{\rm io} = \tag{S95}$$

$$= \quad k_0 \left(\frac{1}{\tau_\pm} + \frac{I-W}{\tau} \right)^{-1} (I-W)^{-1} \left(\frac{I-W}{\tau} + \frac{I-Z}{\tau_{\rm in}} \right)^{-1} (I-Z)^{-1} \frac{B}{2\tau\tau_{\rm in}} + \tag{S96}$$

$$+ \quad \frac{k_0}{\tau} \left(\frac{1}{\tau_\pm} + \frac{I-W}{\tau} \right)^{-1} \left(\frac{1}{\tau_\pm} + \frac{I-Z}{\tau_{\rm in}} \right)^{-1} \left(\frac{I-W}{\tau} + \frac{I-Z}{\tau_{\rm in}} \right)^{-1} (I-Z)^{-1} \frac{B}{2\tau\tau_{\rm in}} \tag{S97}$$

The time-reversible solution can be found by solving

$$W(I-W) \left(\frac{1}{\tau_\pm} + \frac{I-W}{\tau} \right) \left(\frac{I-W}{\tau} + \frac{I-Z}{\tau_{\rm in}} \right) =$$
$$= k_0 \left\{ I + \frac{I-W}{\tau} \left(\frac{1}{\tau_\pm} + \frac{I-Z}{\tau_{\rm in}} \right)^{-1} \right\} (I-Z)^{-1} \frac{BD}{2\tau\tau_{\rm in}}$$

This is a fourth order equation for W and can be solved by noting that W, Z, BD have the same eigenvectors, and solving numerically the fourth order equation for the eigenvalues of W.

Supplementary Figures

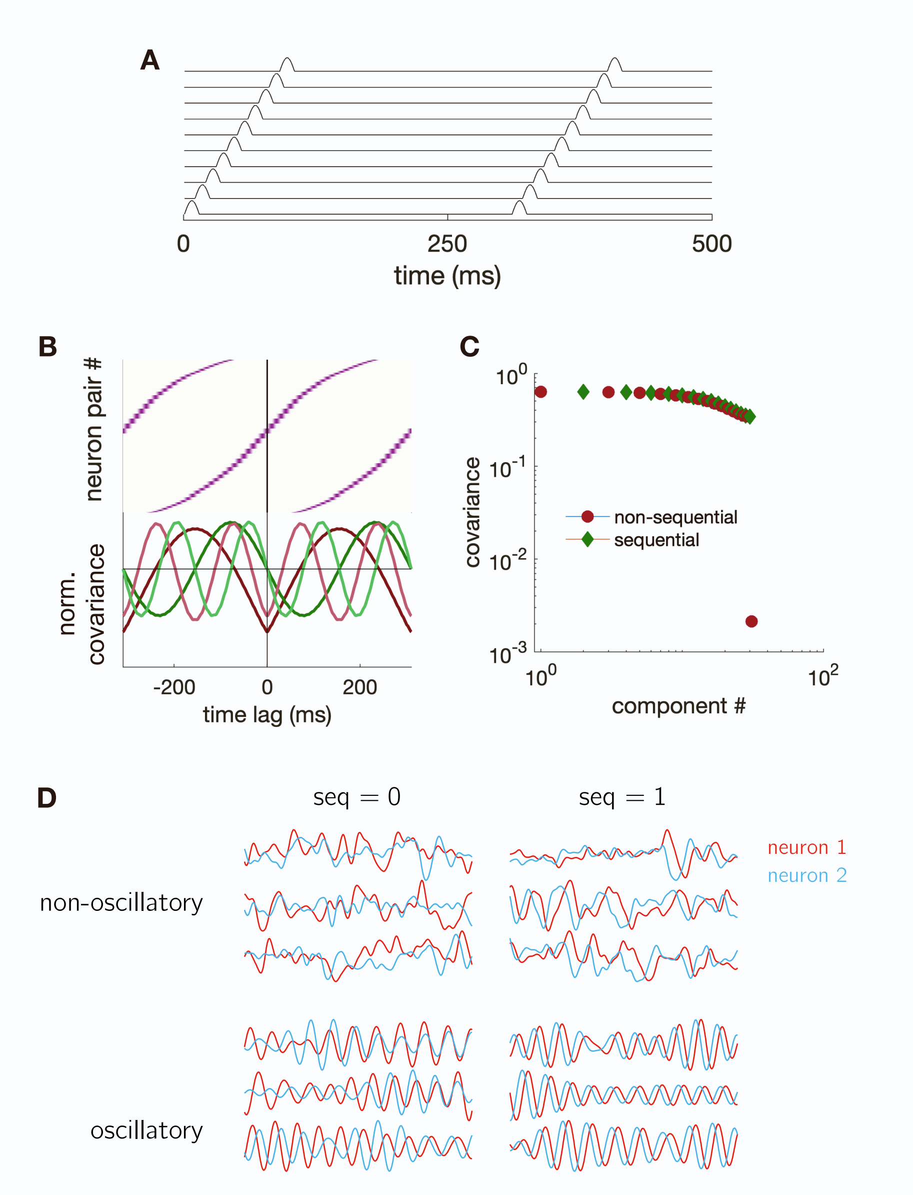

Figure S1: **Examples of sequential and non-sequential activity, and link to synfire chains and neuronal oscillations**, related to Figure 1. **(A)-(C)** A synfire chain (Equation 11; Abeles, 1991) has sequentiality equal to one (seq = 1 in Equations 3 and 10). **(A)** Time series of the activities of the first 10 neurons of a synfire chain (of 31 neurons), showing two activity sweeps across the population. **(B)** Top: time-lagged cross-covariance (CC) functions of the activities of all neuron pairs (as in Figure 1H). Neuron pairs are ordered according to the location of the peak of their cross-covariances. Bottom: first four temporal components (red: symmetric; green: anti-symmetric; as in Figure 1I). **(C)** Sequentiality spectrum (as in Figure 1G): covariance associated with each temporal component of the synfire chain, in decreasing rank order (colors as in the middle panel). **(D)** Our measure of sequentiality is not systematically related to oscillatoriness in neural activity. We consider two neurons whose marginal activity statistics are either smooth but non-oscillatory (top row) or oscillatory (bottom row). The degree of temporal coupling between the two neurons can be controlled independently of these marginals, leading either to zero (left column) or maximal (right column) sequentiality. Three examples are shown for each combination of oscillatoriness and sequentiality. The cases where seq = 1 were constructed following Rutten et al. (2020).

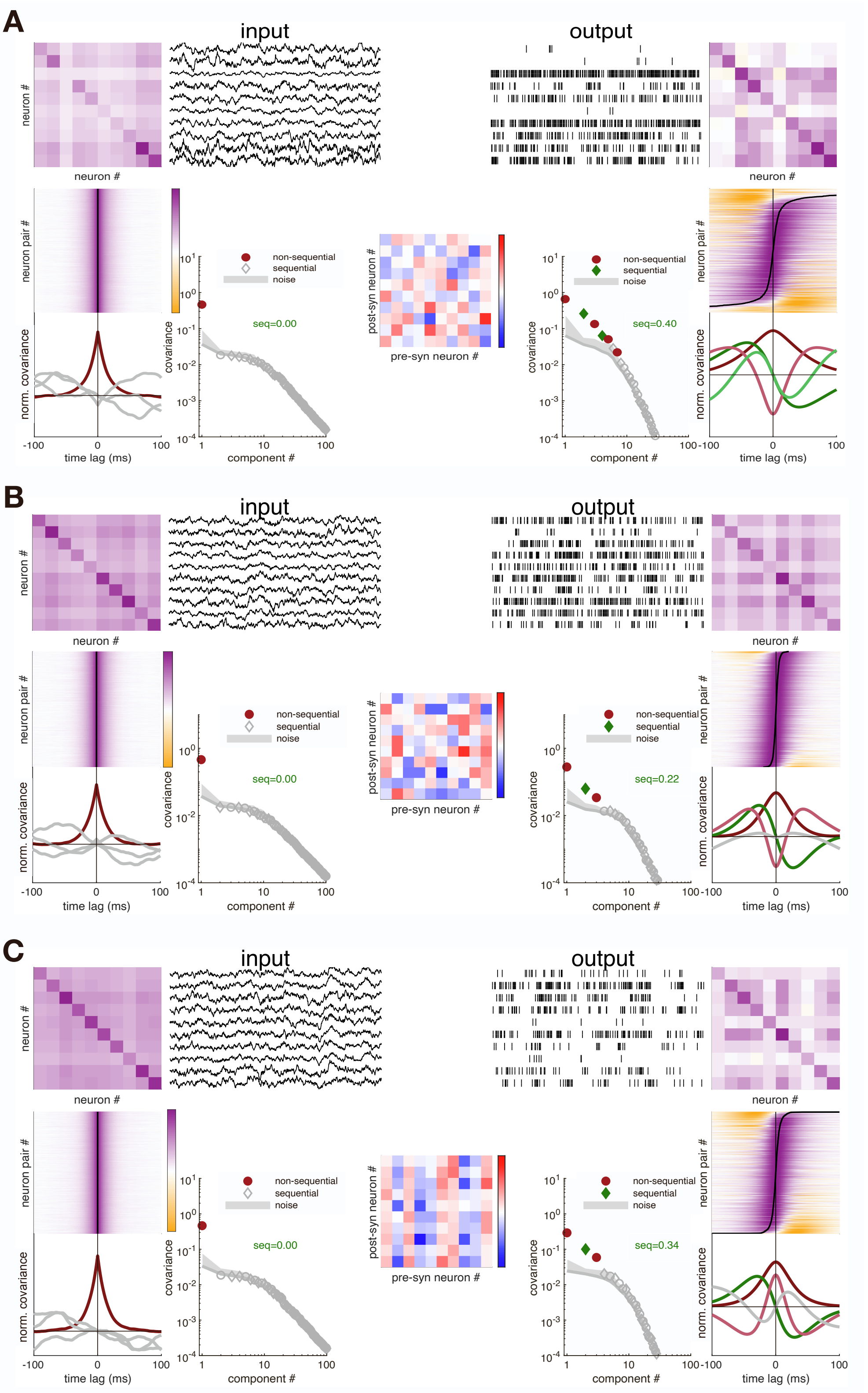

Figure S2: **Sequentiality in example neural circuits with various types of random connectivity**, related to Figure 1. Panels as in Figure 1. **(A)** Random connectivity. Note that, unlike in Figure 1, the connectivity matrix (middle) was not constrained to be symmetric. **(B)** Random antisymmetric connectivity. Note that the connectivity matrix (middle) is skew-symmmetric. **(C)** Random connectivity obeying Dale's law. Note that each column of the connectivity matrix (middle) has all positive or all negative elements.

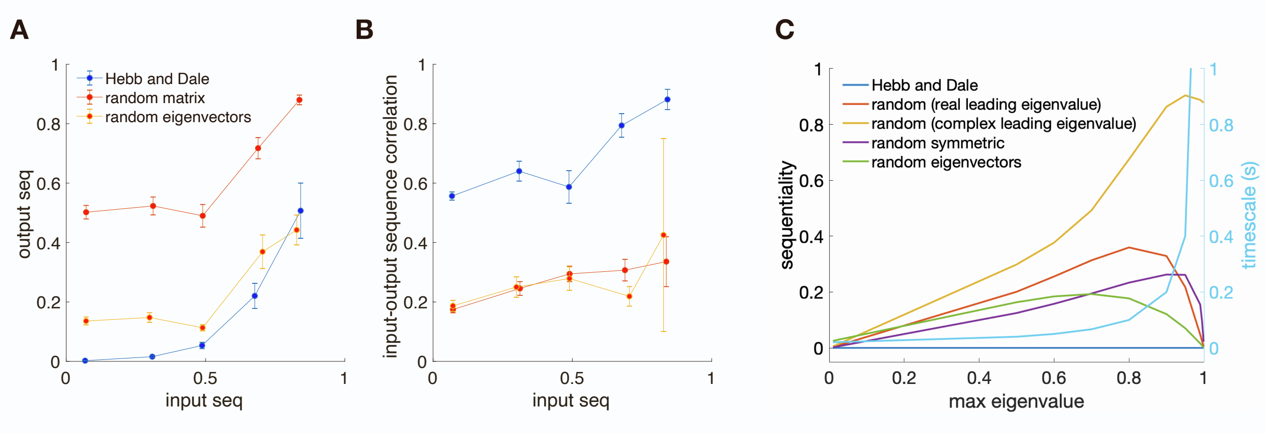

Figure S3: **Hebb-and-Dale connectivity is useful with sequential input or with strong dynamics**, related to Figure 2. **(A)**-**(B)** We compare networks with three different types of synaptic weight matrices: 1) Hebb and Dale (blue) – as explained in the main text (Figure 2E); random matrix (red) – each entry is drawn independently from a Gaussian distribution of zero mean (Figure S2A; matrix is scaled to have the same spectral abscissa as of the Hebb and Dale matrix); random eigenvectors (orange) – all eigenvalues are identical to those of the Hebb and Dale matrix, but each entry of every eigenvector is drawn independently from a Gaussian distribution of zero mean (scale does not matter). For each connectivity type, we simulated 100 networks at each level of input sequentiality, and show mean$\pm$1 s.e.m. across these networks. **(A)** Output vs. input sequentiality. Sequentiality is computed as explained in the main text. When the input sequentiality is zero, Hebb and Dale connectivity is the only one resulting in zero output sequentiality. When input sequentiality increases, output sequentiality increases for all three types of networks. **(B)** Input-output sequence correlation vs. input sequentiality. Input-output sequence correlation is computed by taking the leading skew-symmetric spatial mode of the input, interpreted as the main sequential input pattern, and finding the highest correlation among the leading 10 output patterns. The Hebb and Dale network achieves significantly higher correlation between the input and output sequential patterns than the other networks. **(C)**: Sequentiality (left y-axis) and network timescale (right y-axis) vs. spectral abscissa (i.e. maximum real eigenvalue) of the synaptic weight matrix, for different types of networks. In addition to those shown in panels (A) and (B), we show results for a *random symmetric* matrix (same as "random matrix" in A-B, but with reciprocal connection strengths set to be equal). In the "random matrix" case, we distinguish sampled weight matrices based on whether their leading eigenvalue (that with maximum real part) is real or complex. All matrices are rescaled by an appropriate factor to obtain the desired value of the spectral abscissa (x-axis). The light blue line shows the corresponding timescale of network integration (i.e. $\tau/\left[1 - \text{spectral abscissa}\right]$, identical for all networks). As we show (and as is well-known; Dayan and Abbott, 2001), across a variety of networks, timescales (and overall fluctuation magnitude, not shown) tend to explode when the connectivity is scaled up towards criticality (spectral abscissa = 1). Yet, we find that this does not affect sequentiality in Hebb and Dale networks (dark blue), as it is provably uniformly zero (Supplementary Math Note 2). However, for more general weight matrices, sequentiality tends to exhibit a maximum at some intermediate connectivity strength (other colors). This can be understood by noting that (i) for weak connectivity, the network does not substantially modify the spatiotemporal structure of the input, which we defined to have seq = 0 in this case, and (ii) as the network approaches criticality, activity becomes dominated by the dominant eigenmode and thus becomes effectively one-dimensional (Ganguli et al., 2008) (except for the case when the leading eigenvalue is complex, yellow) – therefore, by definition, it can only be non-sequential.

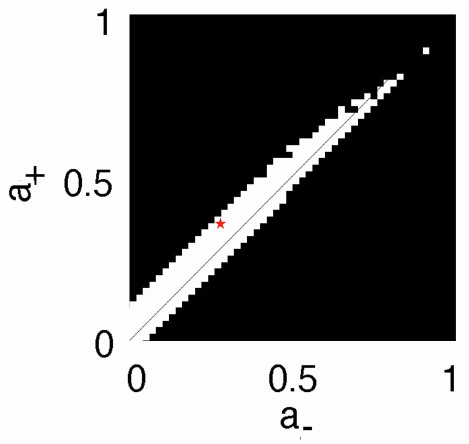

Figure S4: **Stability of STDP learning dynamics**, related to Figure 4. STDP dynamics is stable for a subset of (a_+, a_-) combinations (white region). Red star shows parameter combination used in the simulations of the main text, Figure 4.

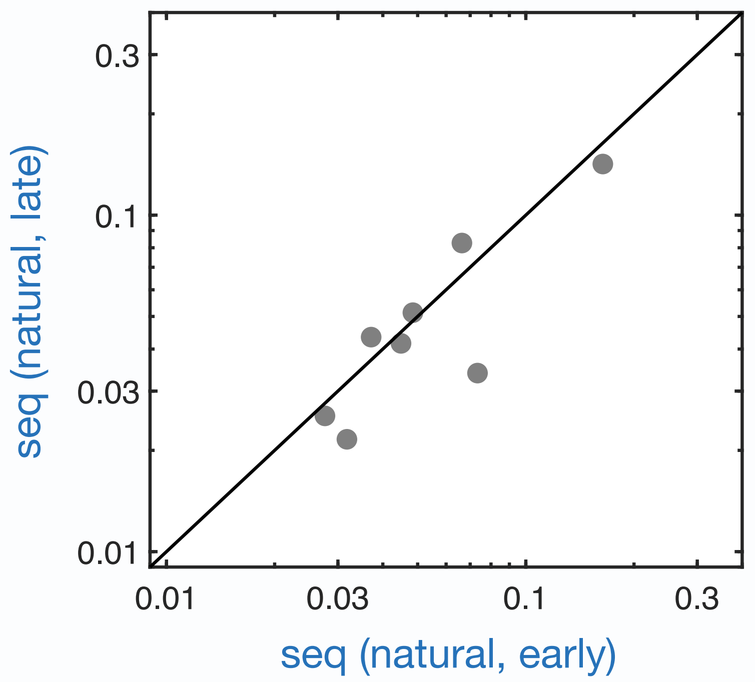

Figure S5: **Temporal changes in sequentiality for natural stimuli.** Same as Figure 5G, but for natural stimuli. Sequentiality of neural responses during the late (y-axis) vs. the early half of exposure (x-axis) to the natural stimuli across animals (dots). Sequentiality is not systematically lower later than earlier, and this does not seem to be due to a ceiling effect (in which case dots with a lower x- or y-coordinate would be closer to the diagonal).
