# Supplementary material for: Adaptive erasure of spurious sequences in sensory cortical circuits
Source: Neuron. 2022 Jun 1;110(11):1857–1868.e5. doi: 10.1016/j.neuron.2022.03.006 (PMC9616807; doi:10.1016/j.neuron.2022.03.006)
Supplement: Document S2. Article plus supplemental information [file mmc2.pdf]

Neuron

Article

Adaptive erasure of spurious sequences in sensory cortical circuits

Highlights

- Recurrent circuits generate spurious sequences without sequential inputs
- A principled measure of total sequentiality in population responses is developed
- Theory predicts that Hebbian plasticity should abolish spurious sequences
- Spurious sequences in the visual cortex diminish with experience

Authors

Alberto Bernacchia, József Fiser, Guillaume Hennequin, Máté Lengyel

Correspondence

ab2349@cam.ac.uk

In brief

Bernacchia et al. developed a theory of sequential activity in recurrent circuits that predicted the prevalence of “spurious” sequences in cortical activity that are unrelated to sequences in the inputs and their experience-dependent erasure by Hebbian plasticity. They confirmed these predictions in visual cortical recordings.

Bernacchia et al., 2022, Neuron *110*, 1857–1868
June 1, 2022 © 2022 The Authors. Published by Elsevier Inc.
https://doi.org/10.1016/j.neuron.2022.03.006

Neuron

CellPress
OPEN ACCESS

Article

Adaptive erasure of spurious sequences in sensory cortical circuits

Alberto Bernacchia,[1,2,6,*] József Fiser,[3] Guillaume Hennequin,[1,4,5] and Máté Lengyel[1,3,4,5]
[1]Computational and Biological Learning Lab, Department of Engineering, University of Cambridge, Cambridge CB2 1PZ, United Kingdom
[2]MediaTek Research, Building 2010, Cambourne Business Park, Cambourne, CB23 6DW, United Kingdom
[3]Center for Cognitive Computation, Department of Cognitive Science, Central European University, Budapest 1051, Hungary
[4]These authors contributed equally
[5]Senior author
[6]Lead contact
*Correspondence: ab2349@cam.ac.uk
https://doi.org/10.1016/j.neuron.2022.03.006

SUMMARY

Sequential activity reflecting previously experienced temporal sequences is considered a hallmark of learning across cortical areas. However, it is unknown how cortical circuits avoid the converse problem: producing spurious sequences that are not reflecting sequences in their inputs. We develop methods to quantify and study sequentiality in neural responses. We show that recurrent circuit responses generally include spurious sequences, which are specifically prevented in circuits that obey two widely known features of cortical microcircuit organization: Dale's law and Hebbian connectivity. In particular, spike-timing-dependent plasticity in excitation-inhibition networks leads to an adaptive erasure of spurious sequences. We tested our theory in multielectrode recordings from the visual cortex of awake ferrets. Although responses to natural stimuli were largely non-sequential, responses to artificial stimuli initially included spurious sequences, which diminished over extended exposure. These results reveal an unexpected role for Hebbian experience-dependent plasticity and Dale's law in sensory cortical circuits.

INTRODUCTION

Sequential neural activity is believed to underlie a variety of functions (Abeles, 1991), such as learning (Hebb, 1949; Bi and Poo, 2001), perception (Wehr and Laurent, 1996), memory (Goldman, 2009; Ólafsdóttir et al., 2018), planning (Pfeiffer and Foster, 2013; Mattar and Daw, 2018), and motor control (Churchland et al., 2012). Indeed, it has been observed in a number of cortical areas (Hoffman and McNaughton, 2002; Mazzucato et al., 2015), including the hippocampus during both navigation and off-line periods (Skaggs and McNaughton, 1996; Diba and Buzsáki, 2007; Gupta et al., 2010; Carr et al., 2011; Lee et al., 2012), the cortical nuclei of songbirds during vocalization (Dave and Margoliash, 2000; Fee et al., 2004; Okubo et al., 2015), and the visual cortex (Ji and Wilson, 2007; Xu et al., 2012; Ekman et al., 2017). The learning of such sequential activity from experience has been proposed to be supported by specific circuit and synaptic plasticity mechanisms, such as spike-timing-dependent plasticity (STDP) (Fiete et al., 2010; Lengyel et al., 2005; Clopath et al., 2010; Gütig and Sompolinsky, 2006; Branco et al., 2010). However, the converse problem has so far been neglected: do spurious sequences, not reflecting sequences that ever appear in the input, also appear in cortical activity? If so, are there mechanisms in place that can erase them? Although such spontaneously generated activity sequences may in some cases be desirable in brain areas involved in motor control (Churchland et al., 2012; Okubo et al., 2015) or in areas undergoing development (Ackman et al., 2012), they will generally be disruptive to the normal operation of sensory areas by interfering with the temporal ordering of responses to external stimuli.

Inspired by the general phenomenon that unusual, often carefully crafted, inputs can result in spurious or atypical neural responses (Rossi et al., 1996; Grosof et al., 1993; Walker et al., 2019; Bashivan et al., 2019) and even lead to overt illusions (Brewster, 1844; Kanizsa, 1955), we hypothesized that sensory cortical circuits not adapted to the statistics of their inputs will be more prone to producing spurious sequences. Conversely, we wondered whether statistical adaptation would lead to the erasure of such spurious sequential activity.

In order to study the appearance and erasure of spurious sequences in sensory cortical circuits, we used a combination of theory, computational modeling of neural circuit dynamics, and analyses of neural recordings of the ferret visual cortex. We developed a measure of sequentiality for multivariate time series and derived theoretical conditions for a recurrent neural network to produce activity that is non-sequential by this measure. Surprisingly, we found that activity sequences tend to arise generically in recurrent networks, even when external inputs are non-sequential. Indeed, eliminating spurious sequences requires that a specific, non-trivial relationship be established

This is an open access article under the CC BY license (http://creativecommons.org/licenses/by/4.0/).

between a network's recurrent connectivity and the statistics of its input. We found that this relationship can be established and maintained via the joint effect of two widely known features of cortical microcircuit connectivity: Hebbian plasticity (connections between co-active cells are stronger) and Dale's law (all efferent connections of a cell are either excitatory or inhibitory). We show that the Hebbian connectivity criterion can be achieved by STDP, which thus serves the erasure of spurious sequences rather than just the learning of new sequences. These theoretical results lead to two key predictions. First, neural responses to natural stimuli—to which connectivity has adapted—should not show spurious sequences. In contrast, artificial stimuli without *any* sequential structure should produce sequential responses that, due to Hebbian plasticity, should abate with experience. We confirmed these predictions directly in electrophysiological recordings from the primary visual cortex of awake ferrets. Taken together, our results establish the avoidance of spurious sequences as a key component of statistical adaptation in sensory circuits (Berkes et al., 2011) and elucidate the mechanisms by which the cortex solves this challenging computational task.

RESULTS

Sequentiality in neural circuits

We began by considering a standard model of a recurrent neural network that describes how the instantaneous firing rate of each neuron changes over time (STAR Methods). Specifically, the momentary firing rate of each neuron i is a nonlinear, non-negative function $f(v_i)$ of an internal activation variable v_i, which models sub-threshold membrane potential fluctuations and evolves according to

$$\tau \frac{dv_i}{dt} = -v_i(t) + \sum_{j=1}^{N} W_{ij} f[v_j(t)] + \xi_i(t) \qquad \text{(Equation 1)}$$

where τ is the membrane time constant and W_{ij} is the strength of the recurrent connection from neuron j to neuron i. In addition to recurrent synaptic interactions with other neurons in the network, each neuron i also receives a time-varying external input, $\xi_i(t)$. Our goal was to understand how statistical covariations in the inputs of pairs of neurons determine statistical covariations between their responses and, in particular, the presence or absence of any temporal ordering between those responses.

In general, natural stimuli may include sequences (e.g., due to self-motion-induced visual flow), potentially leading to sequential patterns of neural responses that could be difficult to identify as input driven rather than spurious. To be able to unequivocally identify any sequential activity appearing in the output of the network as spurious, we first focused on the simple case in which the input to the network had no sequences (Figure 1A). Specifically, we precluded any temporal ordering between network inputs by enforcing temporal symmetry in the input covariance: $C^{\text{in}}_{ij}(\tau) = C^{\text{in}}_{ij}(-\tau)$, where $C^{\text{in}}_{ij}(\tau) = \text{cov}[\xi_i(t), \xi_j(t+\tau)]$ is the covariance between the inputs of neuron i and j at a time lag τ. As we show below, networks that successfully avoid spurious sequences in this non-sequential input regime can also more faithfully represent temporally ordered information in the regime in which inputs are sequential.

As an extreme case, we first considered a network in which connections between neurons were random but entirely symmetric, such that $W_{ij} = W_{ji}$ and the network, therefore, did not embed any directed chains (Goldman, 2009; Figure 1B). Recurrent interactions between the neurons of the network through these connections produced complex, time-varying responses (Figure 1C). The high dimensionality of these responses required us to develop a principled method to quantify how much (if any) sequential activity was present in them (STAR Methods; Note S1).

Our method is based on simultaneously analyzing the time-lagged cross-covariances (CCs) of the responses of all cell pairs in a population, $C_{ij}(\tau) = \text{cov}[f(v_i(t)), f(v_j(t+\tau))]$. Although, by construction, all CCs in the input were time symmetric (Figure 1D), the CCs in the output showed marked asymmetries, resulting in a clear temporal ordering across cell pairs (Figure 1H). Our method decomposes this population of CCs into a set of separable spatio-temporal components, using a computationally efficient and principled generalized singular value decomposition-based approach:

$$C_{ij}(\tau) = \sum_{k=1}^{N^2} \sigma_k A^{(k)}_{ij} f^{(k)}(\tau) \qquad \text{(Equation 2)}$$

where the σ_k are positive constants scaling the overall covariance accounted for by each component, $A^{(k)}$ are (normalized and pairwise orthogonal) spatial components, and $f^{(k)}(\tau)$ are (normalized and pairwise orthogonal) temporal components. Importantly, we were able to show that each of the temporal components, $f^{(k)}(\tau)$, is guaranteed to be either exactly symmetric or anti-symmetric (Figures 1E and 1I; Note S1). Following standard approaches (Machens et al., 2010), we then separated out the genuinely present "signal" components from those "noise" components that arise from measurement errors and finite recording durations, based on their overall covariance, σ_k (Figures 1F and 1G; STAR Methods).

Although in the example we are considering here, the time courses of signal components in the input were all time symmetric (Figure 1E), the output contained several signal components that were temporally anti-symmetric (Figure 1I). To quantitatively compare the sequentiality of input and output, we defined the overall level of sequentiality of each time series ("seq") as the total contribution (root-sum-of-squares) of all its anti-symmetric ("asym") signal components (normalized by the contribution of its symmetric signal components, "sym"; Figures 1F and 1G):

$$\text{seq} = \sqrt{\frac{\sum_{k \in \text{asym}} \sigma_k^2}{\sum_{k \in \text{sym}} \sigma_k^2}} \qquad \text{(Equation 3)}$$

This measure of sequentiality is zero when all covariances are time symmetric (up to noise), and its maximum is one, which can be achieved by a population that persistently generates a single sequence—as in so-called synfire chains (Abeles, 1991; Figures S1A–S1C). (Note that this measure is largely independent of the presence of oscillations as such, as it can be high even in networks that do not oscillate and can be low even in

CellPress
OPEN ACCESS

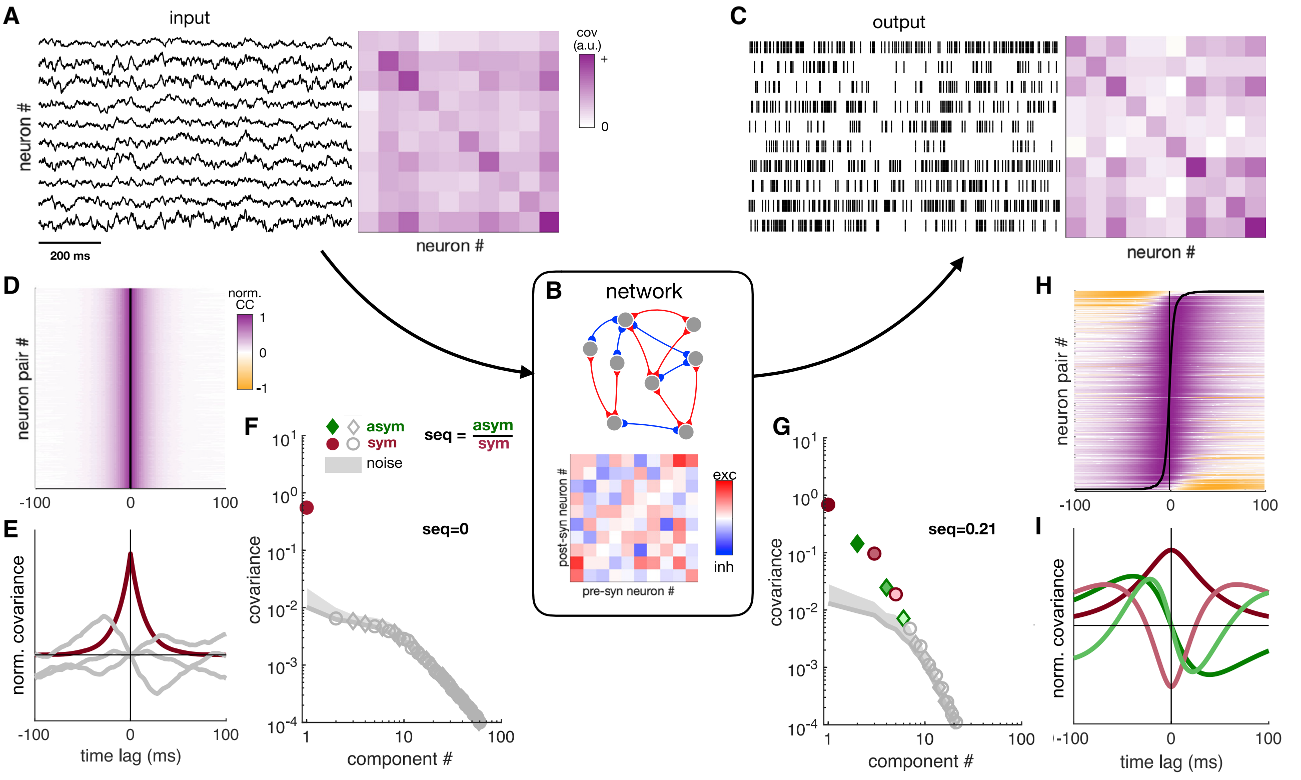

Figure 1. Sequentiality in symmetric neural circuits

(A) Time series (left) and instantaneous covariance (cov) matrix of the inputs ($\boldsymbol{\Sigma} = \mathbf{C}^{\text{in}}(0)$, right) of 10 representative neurons (out of 50).

(B) Schematic of the recurrent neural network model (top) and synaptic connection strengths of the same 10 neurons as in (A) (bottom).

(C) Spiking output of the same 10 neurons as in A (left) and their covariances (right). (Exact spike times are notional, only firing rates are considered.)

(D) Time-lagged CC functions of the inputs of all neuron pairs (each normalized to have a maximum magnitude of 1). For illustration, neuron pairs are ordered according to the location of the peak of their CCs (all at 0 in this case), and black line in the middle connects these peaks across cell pairs (all subsequent measures illustrated in E–G and I are independent of this ordering or the detection of peaks in CC functions).

(E) The first four temporal components of the CC structure of the input to the network (red: symmetric, sym; green: anti-symmetric, asym; gray: noise).

(F) Sequentiality spectrum: total covariance associated with each component of the input, in decreasing rank order (colors as in E). Gray line and shaded area show mean + 7 SD of covariances expected due to limited sample-size effects (STAR Methods). Sequentiality (seq) is computed as the ratio between the total covariance (root-sum-of-squares) carried by significant (non-noise) asymmetric versus symmetric components (Equation 3).

(G–I) Same as (D)–(F) for the output of the network. See also Figure S1 illustrating the behavior of seq for artificial signals with precisely controlled properties and Figure S2 for similar results as shown here with other types of networks.

oscillatory networks when neurons do not have consistent phase relationships; Figure S1D.)

As expected, in the example we studied here, sequentiality was zero for the input by our measure (Figure 1F). However, despite the network being symmetric, the output displayed substantial sequentiality (seq = 0.21; Figure 1G). These results, showing the generation of (spurious) sequential activity in response to non-sequential inputs, generalized to networks with random non-symmetric or random anti-symmetric connections, as well as to random “Dale” networks composed of split populations of excitatory and inhibitory neurons (Figure S2).

A theory of spurious sequences

Given the ubiquity of spurious sequences, as indicated by our simulations, we wondered what mechanisms explained their appearance even under conditions that seemed to maximally work against them (i.e., non-sequential input and symmetric connections). For this, we began by considering a minimal, symmetrically connected circuit motif with only two units, representing individual neurons or small populations. We found that sequentiality arose mainly from two independent properties of the model. First, when these two units received inputs that were unequal in their magnitudes, such that unit 1 received much larger inputs than unit 2, unit 2 became mainly entrained (with some lag) by unit 1 rather than by its own external input (Figure 2A). As a result, the activity of unit 2 trailed that of unit 1. Second, even with balanced input magnitudes, systematic lags between units could also develop due to unequal self-connection strengths (Figure 2B). Self-connections alter the effective time constant with which neural populations integrate their inputs, such that larger self-connections result in slower input integration (Seung, 1996). Thus, in our example, unit 2 with excitatory self-connections trailed unit 1 with inhibitory self-connections. We were able to show (Figure 2D; Note S2) that the presence and magnitude of these two basic motifs together accurately predict the

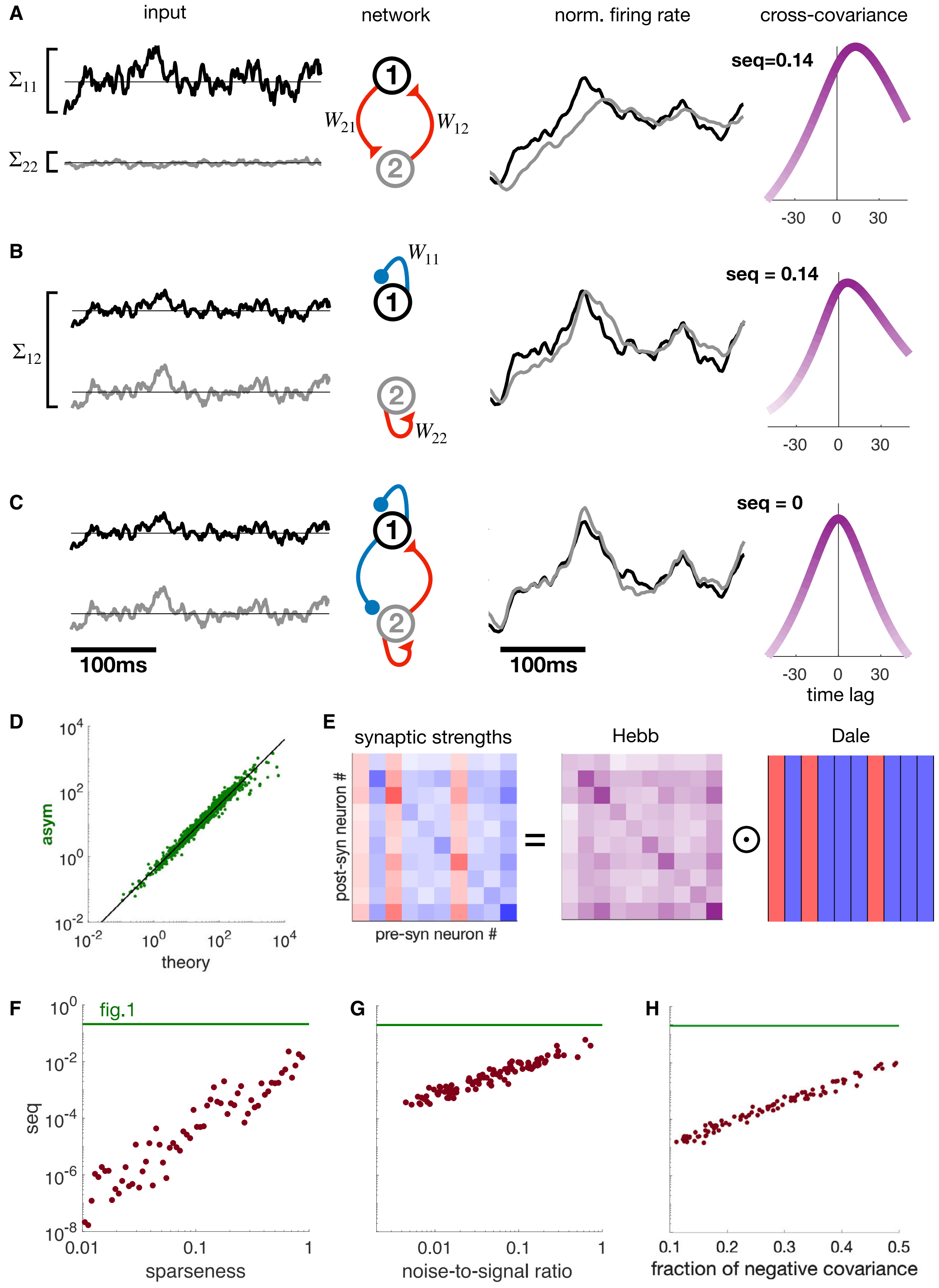

Figure 2. A theory of spurious sequences

(A) A two-unit network with symmetric excitatory connections ($W_{12} = W_{21} > 0$, middle left), receiving inputs of unequal magnitude (variances $\Sigma_{11} > \Sigma_{22}$, far left), produces outputs (shown as normalized firing rates, middle right) such that unit 2 (gray) trails unit 1 (black), as also shown by their CC function and the resulting sequentiality (far right).

(legend continued on next page)

CellPress
OPEN ACCESS

appearance of spurious sequences (i.e., the total amount of temporal anti-symmetry, quantified by the numerator of our sequentiality measure, Equation 3) in a simple mathematical form:

$$\sqrt{\sum_{k \in \text{asym}} \sigma_k^2} \;\tilde{\propto}\; \left| \underbrace{(\Sigma_{22} - \Sigma_{11})\, W_{12}}_{\text{motif A}} + \underbrace{(W_{11} - W_{22})\, \Sigma_{12}}_{\text{motif B}} \right| \quad \text{(Equation 4)}$$

where $\Sigma_{ij} = C_{ij}^{\text{in}}(0)$ is the instantaneous covariance of the inputs of units i and j. The two motifs described above respectively correspond to the first and second terms of this sum.

Importantly, further analysis also identified specific asymmetric connections which, when included in the network, allowed the effects of the two basic motifs to cancel, resulting in zero sequentiality (Figure 2C; STAR Methods). We noted that the primary effect of these additional asymmetric connections was to match the connectivity of the network to the input correlations between neurons, such that the network came to comply with two fundamental principles of the organization of cortical circuits: (1) Hebb's postulate by which connection strengths between neurons are expected to be proportional to their correlations (Hebb, 1949) and (2) Dale's rule, which results in each neuron either exciting or inhibiting all of its postsynaptic targets (Eccles, 1986).

For larger networks (with more than two neurons), the interplay between network connectivity, input statistics, and our sequentiality measure is more complex (STAR Methods; Note S2). Nevertheless, the motifs we identified for two-neuron networks are still relevant. For example, seq = 0 for any network with symmetric connectivity, homogeneous input variances (i.e., absence of motif 1), and homogeneous self-connections (i.e., absence of motif 2). Most importantly, our mathematical theory of sequentiality in non-linear recurrent neural circuits of arbitrary size revealed that the principles of Hebb and Dale continue to predict the erasure of spurious sequences. Specifically, we were able to prove that for any non-sequential input, sequentiality in the output of a network is zero if each connection strength in the network is the product of (1) the covariance between the inputs to the pre- and postsynaptic neurons ("Hebb") and (2) a positive or negative constant that depends only on the presynaptic neuron ("Dale"; Figure 2E). In Note S2, we show that the first factor can alternatively be replaced by the output (instead of input) covariance. Thus, as long as correlations are non-negative, this "Hebb-and-Dale" construction results in connections that satisfy Dale's law.

We verified in numerical simulations that non-sequentiality of the outputs is preserved even when connectivity is sparse (Figure 2F), when connection strengths are only approximately proportional to input covariances (Figure 2G) or when some fraction of input covariances is negative but synapses corresponding to these covariances are pruned to preserve Dale's principle (Figure 2H; seq < 0.01 up to 90% sparseness, 20% noise-to-signal ratio, or 50% negative input covariances). These results show that Dale's law in tandem with Hebb's rule is sufficient for eliminating spurious sequences even in these more realistic scenarios, with only minor, if any, modifications. Interestingly, even when modifications are necessary, these simply result in eliminating some of the synaptic connections, thus improving the biological plausibility of the network, which may otherwise need to be fully connected.

Although our focus was primarily on the erasure of spurious sequences in response to purely non-sequential inputs, we also confirmed that Hebb-and-Dale connectivity remains useful even in the more general case, when the input to a network can contain sequences. Specifically, we found that more sequential inputs resulted in increasingly sequential outputs in this case (Figure S3A) and that output sequences represented input sequences substantially better than in networks with random connectivity (matched for basic properties of the resulting network dynamics; Figure S3B). In addition, for non-sequential inputs, we could also show that Hebb-and-Dale networks maintain zero sequentiality over a broad range of intrinsic network time constants. This was particularly notable in networks with connections strong enough as to induce long (but finite) timescales in their dynamics (Ganguli et al., 2008), as networks with random connectivity produced strongly sequential inputs in that case (Figure S3C).

Hebbian plasticity erases spurious sequences

Based on our theoretical results, we predicted that excitatory-inhibitory cortical circuits (which already satisfy the theory's

(B) Same as (A), for a network of two units with only self-connections of opposite signs ($W_{11}<0<W_{22}$, middle left), receiving strongly correlated inputs (covariance $\Sigma_{12} \gg 0$, far left).

(C) Same as (B), after adding specific asymmetric connections between the two units of the network (middle left), making its output temporally symmetric (middle and far right).

(D) Theoretical prediction of Equation 4 matches well the actual total anti-symmetry of the activity of 2-node symmetric networks. 1,000 randomly generated networks are shown (green dots). Black diagonal is the identity (note that both axes are logarithmic).

(E) Theoretical condition for avoiding spurious sequences: a network of any size produces no sequentiality if its synaptic strengths are the product of a term that is proportional to neural correlations (Hebb) and a term that has the same sign for all efferent synapses of each neuron (Dale, $\odot$ denotes element-wise product of matrices).

(F–H) Sequentiality (seq) for more realistic networks whose connectivity increasingly deviates from the theoretical "Hebb-and-Dale" optimum (cf. E). In all cases, 100 random 50-neuron networks were first generated according to the theory and their connectivity was then perturbed (red dots). Green line shows sequentiality of the network shown in Figure 1 for reference, note the order-of-magnitude difference (logarithmic y axis).

(F) Sparseness: a fraction of the connections were removed, starting from the weakest and resulting in sparser connectivity.

(G) Imprecision of Hebbian plasticity (noise-to-signal ratio): noise was added to the original Hebbian component (signal) of synaptic strengths.

(H) Negative correlations: a fraction of input correlations were negative, and the corresponding connections were removed to preserve Dale's law.

See also Figure S3 comparing the responses of Hebb-and-Dale networks with those of various random networks to sequential inputs, and their respective behaviors at a range of intrinsic network time constants for non-sequential inputs.

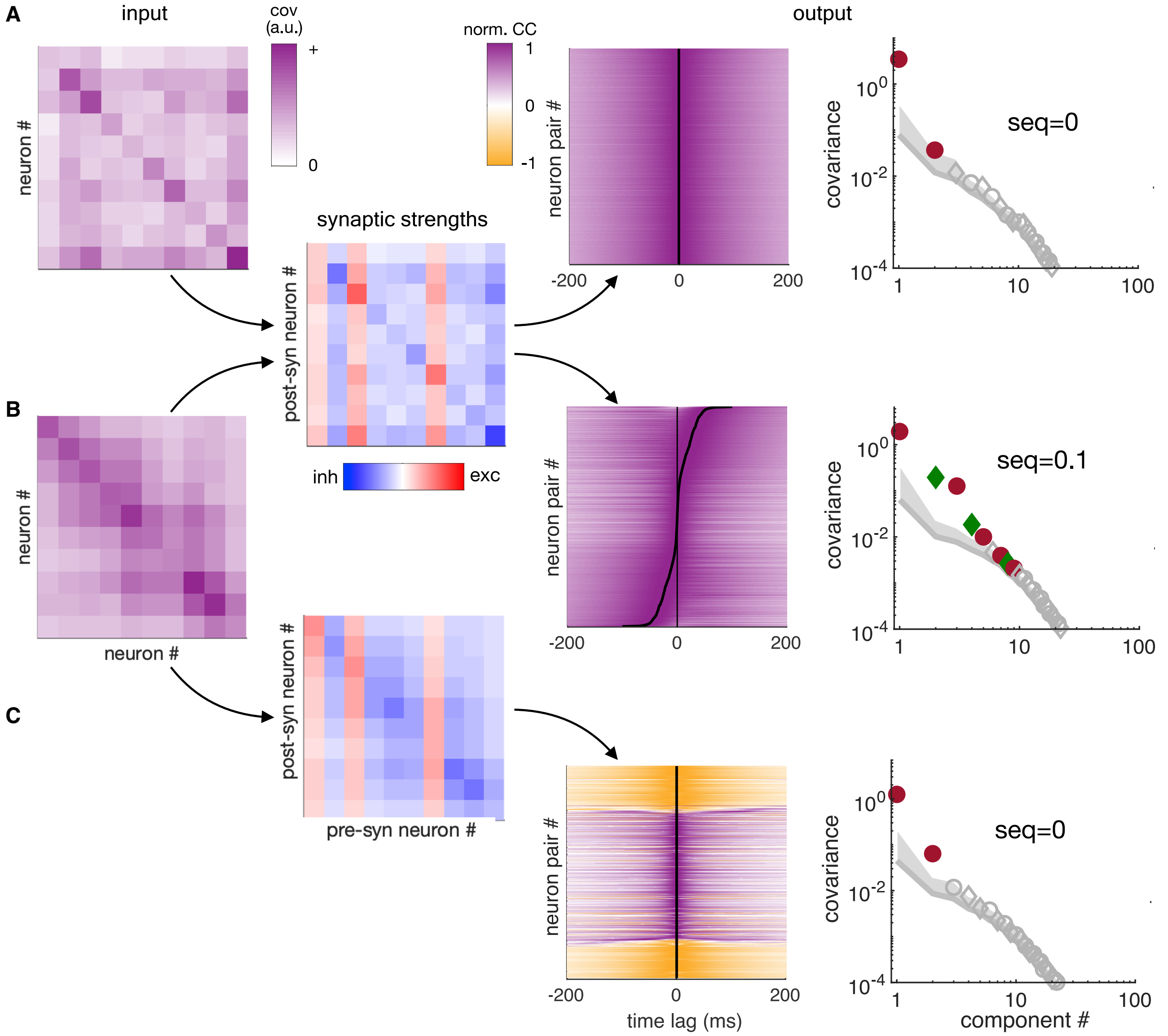

Figure 3. Adaptive erasure of spurious sequences in a model network

(A) A neural network with Hebb-and-Dale synaptic connections (middle, cf. Figure 2E) receiving input to which it has undergone Hebbian adaptation (left, input covariance shown as in Figure 1A) produces non-sequential output (right, CCs and sequentiality spectrum shown as in Figures 1G and 1H).

(B) The same network as in (A) receiving input with a different input covariance structure (left) produces sequential output (right).

(C) The network receiving the same input as in (B) but after its synaptic connections adapted to the input covariance structure (middle) once again produces non-sequential output (right).

Dale requirement) will exhibit or eliminate spurious sequences depending on whether their connection strengths have undergone Hebbian adaptation to their inputs. To test these predictions, we first simulated a network whose synaptic connections were of Hebb-and-Dale form as required by the theory (Figure 3A, left and middle). As predicted, its output was time symmetric (seq = 0; Figure 3A, right). We then simulated the effect of a sudden change of input statistics by feeding the network with an input that had the same non-sequential temporal statistics but a different set of pairwise correlations between neurons, all the while keeping synaptic connections in the network unchanged (Figure 3B, left). As a result, the network exhibited spurious sequential activity (seq = 0.1; Figure 3B, right). Finally, we simulated the effects of Hebbian plasticity in the network under these changed input conditions by setting the connection strengths to be proportional to the new input correlations (Figure 3C, middle). The adapted network again showed no spurious output sequences (seq = 0; Figure 3C, right).

What form of plasticity might give rise to the Hebb-and-Dale synaptic connectivities that are necessary for avoiding spurious sequences? We reasoned that STDP, a prototypical form of Hebbian plasticity (Bi and Poo, 2001; Gerstner et al., 1996;

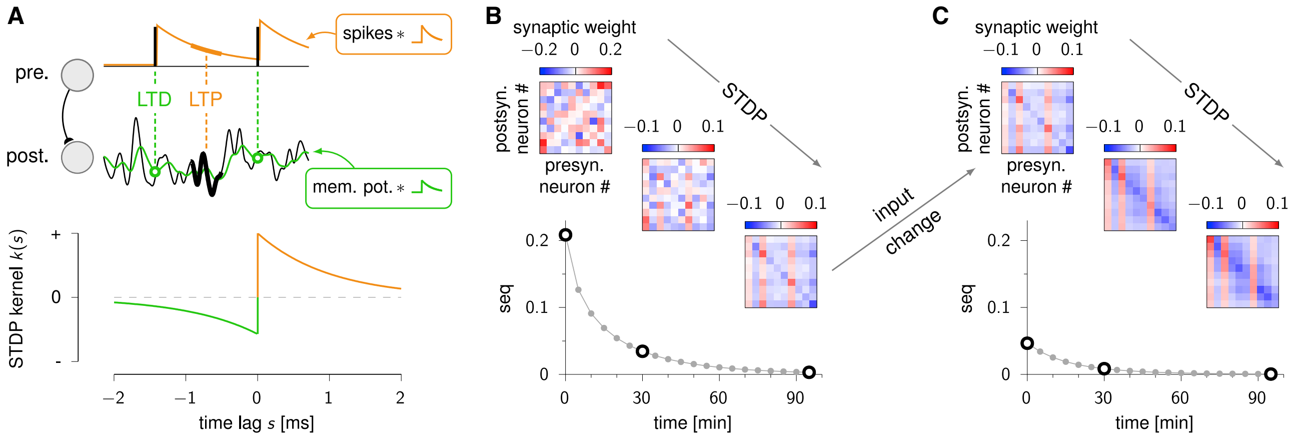

Figure 4. STDP adaptively eliminates spurious sequences

(A) Top: schematic with a pair of synaptically connected cells (left) and with a sample of their activity and quantities determining synaptic modification according to a standard model of STDP (Clopath et al., 2010; right). Each presynaptic spike (top, black ticks) contributes an exponentially decaying waveform (orange inset) to the synaptic eligibility trace (top, orange curve). The postsynaptic membrane potential (bottom, black trace) is passed through a low pass filter (green inset) to produce a running average (bottom, green trace). The synapse is strengthened continually (orange, LTP) in proportion to the presynaptic eligibility trace and the (unfiltered) postsynaptic membrane potential and weakened at the time of presynaptic spikes (green, LTD) in proportion to the filtered postsynaptic membrane potential. Bottom: effective STDP kernel, $k(s)$, describing the change in synaptic strength as a function of the lag between pre- and postsynaptic activity, s. Orange half is the presynaptic eligibility waveform, green half is the time- and sign-reversed postsynaptic filter waveform. Rule shown is for synapses in which the presynaptic cell is excitatory, the same rule with the opposite sign applies to synapses with inhibitory presynaptic neurons. Beside the additive term shown here, synaptic modifications also included a (multiplicative) weight decay (not shown), ensuring that synaptic strengths remained bounded (STAR Methods).

(B) Sequentiality index during the course of STDP, starting from the connectivity matrix shown in Figure 1B and under the input statistics of Figure 1A. Insets show snapshots of the synaptic connectivity matrix at the times shown by the three large black circles. Note the similarity of the final weight matrix to the direct Hebb-and-Dale solution of Figure 3A.

(C) Continuation of (B), after a sudden change in input statistics to that of Figure 3B. Note the similarity of the final weight matrix to the direct Hebb-and-Dale solution of Figure 3C. For efficiency, we did not simulate individual spikes in (B) and (C). Instead, we used a rate-based description of neural responses and an analytical approach to compute both the corresponding average changes in synaptic connections (Kempter et al., 1999; Dayan and Abbott, 2001) and the resulting sequentiality index (STAR Methods; Note S3). Stability of STDP learning dynamics is illustrated in Figure S4.

Morrison et al., 2008), may be a natural candidate. Intriguingly, we were able to show that the connectivities produced by STDP do not have the specific Hebb-and-Dale form we have studied so far. In particular, despite STDP changing synaptic connectivities depending on neuronal output covariances, the “Hebb” part of the resulting connectivities is not simply proportional to neuronal input or output covariances (Figure 2C; Note S3). Nevertheless, we were also able to show that, under broad conditions, STDP gives rise to more general forms of Hebb-and-Dale connectivities that still guarantee the absence of spurious sequences. This is true irrespective of the precise shape of the function describing the dependence of potentiation and depression on the relative timing of presynaptic spikes and postsynaptic activity (Figure 4A), so long as synaptic strengths remain bounded and STDP acts on excitatory and inhibitory synapses alike (with opposite signs; Vogels et al., 2013; Hennequin et al., 2017; see also Figure S4). To illustrate this, we revisited the example of Figure 3, now using STDP to explicitly model the time course with which synaptic connectivity and thus the sequentiality of network responses changes over time (Figures 4B and 4C). Specifically, we assumed that the network’s inputs had the same statistics as those used in Figure 3A, but we initialized the connectivity of the network to the non-Hebb-and-non-Dale configuration shown in Figure 1B. This resulted in initially sequential activity (seq $\simeq$ 0.2), which gradually decayed to zero with the ongoing action of STDP (Figure 4B). In fact, synaptic connections converged to a very similar configuration as in the direct Hebb-and-Dale solution we used above (Figure 3A). Note that in this example, the initial connectivity did not respect Dale’s law but the final connectivity did. Thus, STDP in the model not only tuned the strengths of synapses to make them Hebbian with respect to the stimulus statistics but occasionally also flipped their signs such that the network came to comply with Dale’s law. Finally, following a sudden change in input statistics and thus a sudden jump in sequentiality, as in Figure 3B, ongoing STDP gradually adjusted the synaptic strengths such that spurious sequences were once more eliminated (Figure 4C). Indeed, synaptic connections reached a configuration similar to the direct Hebb-and-Dale solution of Figure 3C. In this case, the Dale structure of connectivity was preserved throughout adaptation, while connection strengths gradually became Hebbian with respect to the new stimulus statistics.

Adaptive erasure of spurious sequences in the ferret visual cortex

Finally, we analyzed neural recordings in the primary visual cortex (V1) of awake ferrets to test the experience-dependent erasure of spurious sequences that our theory and simulations predicted (Figure 5). For this, we used neural activity in animals between postnatal days 44 and 151, i.e., after the maturation of orientation tuning and long-range horizontal connections (Sengpiel and Kind, 2002). As stimuli, we used natural-scene

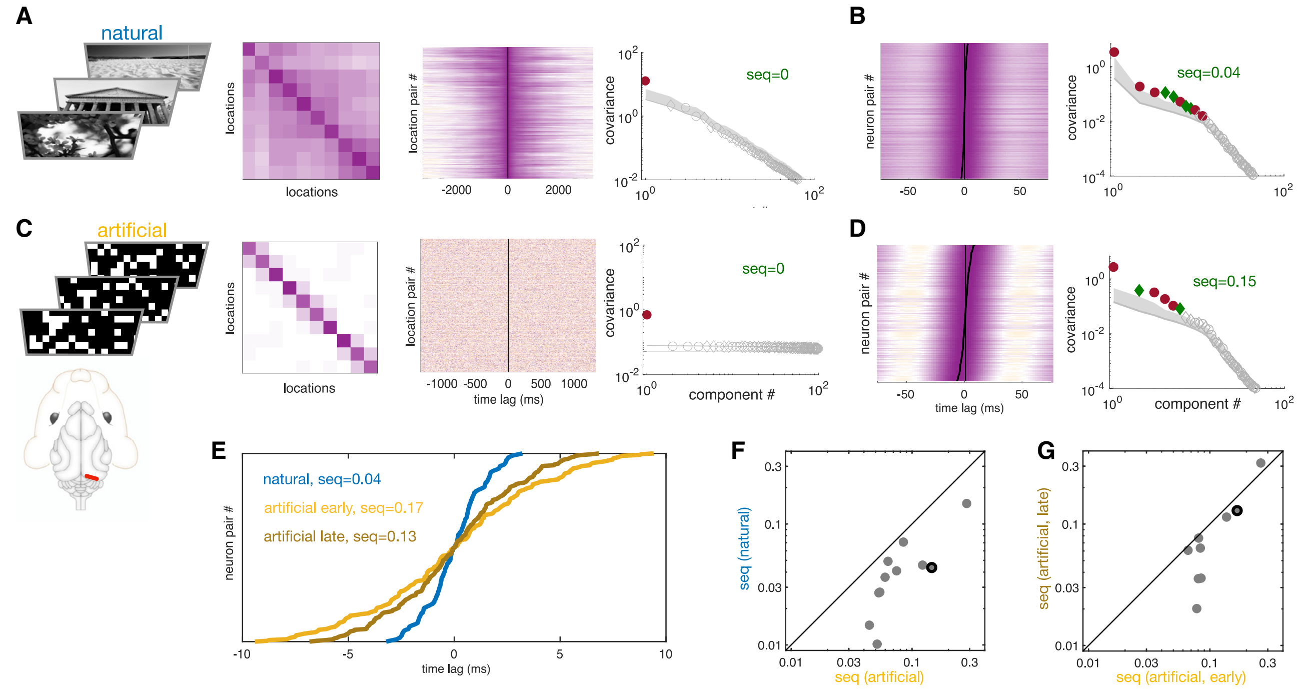

Figure 5. Adaptive erasure of spurious sequences in the ferret visual cortex

(A) Natural movie stimuli: three example frames (far left), the input covariance (middle left), CCs (middle right), and sequentiality spectrum (far right) for 10 representative pixels (cf. Figures 1A, 1D, and 1F). Stimuli shown are for illustration only; stimuli actually used are not shown for copyright reasons (STAR Methods). Analysis shown is for actual stimuli.

(B) Neural responses to natural video stimuli in a representative animal: CCs (left) and sequentiality spectrum (right) for the 16 channels recorded (cf. Figures 1H and 1G). Sequentiality is low (seq = 0.04).

(C and D) Same as (A and B) for artificial (block noise) stimuli. Sequentiality of neural responses is higher (seq = 0.15). Red strip in ferret head schematic illustrates approximate electrode location.

(E) Adaptation to continued exposure to artificial stimuli. Peaks of CCs for the same animal (cf. black line in B and D): sequentiality is higher in the first half of the experimental session (yellow, seq = 0.17) and decreases in the second half of the experimental session (brown, seq = 0.13). CC peaks for natural stimuli are shown for reference (blue, seq = 0.04).

(F) Sequentiality of neural responses to artificial (y axis) versus natural stimuli (x axis) across animals (dots). Sequentiality is lower for natural stimuli for all animals ($p = 0.0001$, $n = 11$).

(G) Sequentiality of neural responses during the late (y axis) versus the early half of exposure (x axis) to the artificial stimuli across animals (dots). Sequentiality is lower later in all animals but one ($p = 0.037$, $n = 9$ after excluding two animals whose sequentiality fell to zero due to limited sample size when restricting analysis to half of the data). See Figure S5 for the same analysis for responses to natural stimuli. Dots with black contour in (F) and (G) indicate the animal whose data are shown in (B), (D), and (E).

movies (Fiser et al., 2004; Berkes et al., 2011), which showed symmetric temporal correlations and thus no sequentiality by our measure (seq = 0, Figure 5A). Using such non-sequential stimuli was consistent with our theoretical analyses and ensured that any sequentiality in neural responses could be unequivocally identified as spurious. We expected the V1 of these animals to have already been adapted to the statistics of these stimuli. In line with our predictions (cf. Figure 3A), V1 activity showed only very weak sequentiality in this case (Figures 5B and 5F, seq = 0.04 for the animal shown). We then used random block noise stimuli, which were strictly temporally symmetric by construction (seq = 0; Figure 5C) and to which we expected V1 not to have adapted (cf. Figure 3B). Again, in line with predictions, V1 responses became substantially and significantly more sequential than responses to natural stimuli, despite the stimuli being perfectly non-sequential in this case (seq = 0.15, Figure 5D versus Figure 5B; Figure 5F, 11 animals showing non-zero sequentiality, $p = 0.0001$, paired t test). Moreover, when comparing the first and last 12.5 min of stimulation with these artificial stimuli, we also found a small but significant decrease in sequentiality over continual stimulation (Figure 5E, yellow versus brown; Figure 5G, $p = 0.037$, paired t test), in line with our predictions about the effects of ongoing adaptation to novel inputs (cf. Figure 3C). As a control, there was no such difference between early and late stimulation with natural stimuli (Figure S5, $p = 0.29$, paired t test), excluding the possibility that the temporal change we found for artificial stimuli was simply due to general fatigue-, arousal-, or electrode displacement-based (or similar, stimulus-independent slow-timescale) effects.

DISCUSSION

For neural circuits to reliably process sequential sensory information, they need to both store experience-dependent

CellPress
OPEN ACCESS

sequences and avoid generating spurious sequences. While most previous work focused on how experience-driven sequences are stored and recalled (Abbott and Blum, 1996; Lengyel et al., 2005; Fiete et al., 2010; Brea et al., 2011), our results highlight the potential prevalence of spurious sequential activity in neural circuits and provide theoretic tools for its study. Hebbian plasticity has been well established as a key component in storing sequential information (Abbott and Blum, 1996; Lengyel et al., 2005; Fiete et al., 2010; Brea et al., 2011). Our analyses show, for the first time, that Hebbian mechanisms can also eliminate spurious sequences when acting in conjunction with Dale's law. These results suggest that the two main organizing principles of cortical circuits, Hebbian plasticity and Dale's law, together play an important role in sequential information processing and, more broadly, in the statistical adaptation of sensory cortical responses (Berkes et al., 2011).

Measuring neural sequentiality

In order to quantify sequentiality in the responses of a neural population rigorously, in a way that is directly relevant to testing the predictions of our theory, we developed a measure of sequentiality. There have been multiple previous proposals for characterizing sequences in neural population responses. Some of these methods focus on identifying individual sequences within a distribution of responses, which were recorded over multiple trials or over an extended time period (Nádasdy et al., 1999; Ikegaya et al., 2004; Mackevicius et al., 2019). Another set of methods measures the sequentiality of responses in individual trials, rather than across a distribution, by assessing the presence of particular pre-defined spatio-temporally regular motifs (Orhan and Ma, 2019; Zhou et al., 2020). Whether the motifs or sequences are pre-defined or identified in a data-driven way, both these sets of methods provide an overall measure of sequentiality, if at all, only once individual motifs or sequences have been reliably determined. In contrast, our method sidesteps determining particular motifs or sequences and provides a direct measure of sequentiality. The advantage of our method is that it does not require strong prior assumptions about the nature of motifs in neural responses while also avoiding the sensitivity of sequence identification to noise (Mackevicius et al., 2019). It achieves this by automatically separating sequential, non-sequential, and noise components of the high-dimensional time series of the inputs and outputs of a neural circuit.

An obvious disadvantage of our method is that when responses are sequential, it alone does not identify the actual sequences they contain—though such extensions are possible (Rutten et al., 2020a, 2020b). Furthermore, while other methods effectively assess sequentiality via complex nonlinear features, our method only uses second-order information. Although this restriction enabled us to develop a complete analytical theory of sequentiality in stochastic recurrent neural networks, there are cases where activity sequences cannot be detected in spatio-temporal covariances alone. In particular, if a circuit generated a sequence in the forward and reverse directions in approximately equal measure, its responses would erroneously appear to be non-sequential when quantified by our method. Nevertheless, it is unclear how severe a limitation this is in practice. For example, when analyzing hippocampal place cell responses while an animal traverses the same linear track back-and-forth, one might expect a balanced distribution of forward and reverse neural sequences corresponding to the two running directions. However, in such cases, outward and inward journeys activate two largely non-overlapping ensembles of place cells instead of the same ensemble (McNaughton et al., 1983). Moreover, during periods of inactivity, although the replay of either of these sequences in the hippocampus is known to occur in both the forward and reverse direction, forward replay is significantly more frequent than reverse replay (Diba and Buzsáki, 2007; Davidson et al., 2009), thus again avoiding the collapse of second-order sequentiality, which could otherwise mislead our method.

Sequential versus non-sequential inputs

In our analyses, we primarily focused on the cases where inputs to a sensory cortical population are non-sequential. There is good reason to believe that this scenario may not be unrealistic, and natural stimuli will generally lead to temporally symmetric input covariances. First, whereas different neurons in an upstream area may have different onset latencies, realistic levels of divergence and convergence of connections between the two areas can effectively average out these differences. Second, onset latencies are usually established using artificial stimuli with hard onsets (Euler and Masland, 2000; Nowak et al., 1995), which rarely occur in natural stimuli. Conversely, the smoother transitions in natural stimuli will typically diminish differences between the onset latencies of different neurons. Third, different delays between the inputs of different cells in naturalistic conditions can be expected to be dominated by the (temporal) statistics of natural stimuli (which may sequentially excite the receptive fields of appropriately ordered neurons), rather than different physiological delays. Indeed, with sequentially presented stimuli, onset latencies were found to be history dependent (Bair et al., 2002). Critically, the statistics of these input sequences can easily average out in natural stimuli. Our analysis showing the lack of consistent sequentiality in naturalistic video stimuli at the level of pixels (Figure 5A) and V1 activity in response to these stimuli (Figure 5B) provides support for this effect. Nevertheless, we also studied the case of non-sequential inputs and found Hebb-and-Dale networks to show distinct functional advantages even in that domain (Figure S3A).

Negative neural correlations and Dale's law

Our theory predicts that in "Hebb-and-Dale" connectivities that successfully eliminate spurious sequences, Dale's law only arises provided the covariances in the Hebbian component are all non-negative. These covariances correspond to "signal" correlations in studies of neural coding (Averbeck et al., 2006) and may be negative in sensory areas (e.g., due to ON-OFF opponency; Mastronarde, 1989). As such, they appear to compromise the role of Dale's law in the suppression of spurious sequentiality. However, in our formalism, the experimentally found negative signal correlations relate to output, not input, covariances. Although balanced network dynamics tend to yield equal proportions of positive and negative output correlations (Renart et al., 2010), the correlations between the total external inputs received by different neurons are likely to be large and

positive on average due to the shared and excitatory nature of these inputs. Importantly, according to our theory, recurrent connections adhering to Dale's law guarantee the absence of spurious sequences when input covariances are positive.

STDP and Dale's law

Our analysis of the effect of STDP on synaptic connectivity suggests an unexpected potential connection between STDP and Dale's law. STDP is commonly thought of as an activity-dependent plasticity mechanism that ensures that the efficacies of synapses obey Hebbian principles (more co-active neurons are more strongly connected; Bi and Poo, 2001). Our results confirmed this role for STDP. However, we showed that when inputs to a network are non-sequential, STDP can also ensure that the "signs" of connections in a network respect Dale's law, i.e., that all efferent synapses of a cell are either excitatory or inhibitory. Dale's law is usually considered to be developmentally predetermined via the primary neurotransmitter released by a given cell type (Kandel et al., 2013). Nevertheless, there is evidence that it can also be altered by plasticity. For example, neurotransmitter expression has been shown to be under the control of activity-dependent mechanisms (Spitzer, 2012). Moreover, the postsynaptic action of even the same neurotransmitter—the ultimate determinant of the "sign" of a synapse—might depend on the receptors expressed postsynaptically and local ionic driving forces. Changes in each of these factors have been shown to be able to switch between excitation and inhibition in synapses using the same neurotransmitter (Liu and Wilson, 2013; Raimondo et al., 2012). It remains to be demonstrated whether some combination of these (and perhaps some yet-unknown) mechanisms are sufficient to control the sign of individual synapses in an activity-dependent manner and in accordance with Dale's law, in the way STDP in our model can.

Experimental tests

Based on our theoretical predictions, we found experimental support for the adaptive erasure of spurious sequences in ferret V1. This data set was ideal for testing our theory, as it satisfied three important requirements. First, recordings need to be performed during normal operation (as opposed to, e.g., development or off-line periods) in a sensory area (as opposed to motor areas) such that the erasure of spurious sequences can be expected to be a priority for the underlying circuit. Second, recordings need to be sufficiently long to allow the reliable estimation of CCs, which in general are noisy such that acceptable signal-to-noise ratios require averaging over many trials (or, equivalently, long recordings). In fact, what matters primarily is the overall number of action potentials recorded per neuron, with more quiet neurons typically calling for longer recordings. We estimate that our approach requires, on average, several tens of thousands of spikes in each individual unit. Third, the temporal resolution of our ferret data set was fine enough that we could reliably estimate the sequentiality index. In particular, it is important that the sampling frequency be large enough to enable the detection of systematic lags between neurons, even when their CCs peak at short lags. In the ferret data used in this study, multi-unit recordings have a resolution of 2 ms, and the asymmetries in the cross-correlations occur on a timescale of about 10 ms. Detecting such asymmetries would be harder in, e.g., calcium imaging data, which suffer from poorer temporal resolution.

A straightforward prediction of our theory is that rearing animals under artificial stimulus statistics (e.g., block noise) should revert our main experimental result and lead to less sequentiality for that specific class of artificial stimuli and more sequentiality when tested with natural stimuli (i.e., dots above rather than below the diagonal in Figure 5F). Alternatively, in normally reared animals, more extensive stimulation with artificial stimuli should not only lead to less sequentiality in the late period of the artificial condition (i.e., dots further below the diagonal than in Figure 5G) but eventually also to an increase in sequentiality during (at least the early period of) the natural stimulus condition (blue line in Figure 5E becoming less steep). In fact, simply introducing another artificial stimulus statistics following adaptation to the first one should also lead to an (at least transient) increase in sequentiality. This would also eliminate a potential shortcoming in our current approach, which uses only one class of artificial stimuli. Namely, we found a decrease of sequentiality for the artificial stimuli over time and, as a control, that no such decrease occurs for natural stimuli. However, we cannot exclude the possibility that this difference is due to more fundamental differences in the ways natural and artificial stimuli are processed upstream of V1, which are beyond the differences between their covariance structure and which we did not control. Comparing the processing of two different but "equally" artificial stimulus sets instead would provide a stronger control.

Finally, our theory also predicts that inhibitory synaptic strengths should be proportional to neural correlations (Figure 2E). This prediction has recently been confirmed in the mouse visual cortex (Ko et al., 2011; Cossell et al., 2015; Znamenskiy et al., 2018) and corroborated by Hebbian synaptic plasticity rules found in GABAergic synapses (Vogels et al., 2013; Hennequin et al., 2017). Together with earlier results on experience-dependent sequential activity, our work lays the foundations for a unified understanding of how circuits can use neural sequences to faithfully represent temporal ordering of information in the environment and to drive sequential behavior.

STAR★METHODS

Detailed methods are provided in the online version of this paper and include the following:

- KEY RESOURCES TABLE
- RESOURCE AVAILABILITY
  - Lead contact
  - Materials availability
  - Data and code availability
- EXPERIMENTAL MODEL AND SUBJECT DETAILS
- METHOD DETAILS
  - Animal preparation and data acquisition
  - Visual stimulation
  - Neural circuit model
  - Parameter values
- QUANTIFICATION AND STATISTICAL ANALYSIS
  - Cross-covariance
  - Sequentiality

CellPress
OPEN ACCESS

- ○ Noise estimation
- ○ Data analysis and exclusion

SUPPLEMENTAL INFORMATION

Supplemental information can be found online at https://doi.org/10.1016/j.neuron.2022.03.006.

ACKNOWLEDGMENTS

This work was supported by the Wellcome Trust (Investigator Award in Science 212262/Z/18/Z to M.L. and Seed Award 202111/Z/16/Z to G.H.) and the Human Frontiers Science Programme (research grant RGP0044/2018 to M.L.). We thank G. Orbán for allowing us to use his photographic pictures and ferret brain schematic in Figures 5A and 5C. For the purpose of open access, the authors have applied a CC BY public copyright license to any Author Accepted Manuscript version arising from this submission.

AUTHOR CONTRIBUTIONS

A.B. developed the theoretical framework, performed analytical derivations, and analyzed neural data. A.B. and G.H. performed numerical simulations. A.B., G.H., and M.L. conceptualized the study, interpreted the results, and wrote the manuscript. J.F. provided experimental data and input for the manuscript.

DECLARATION OF INTERESTS

The authors declare no competing interests.

Received: February 23, 2021
Revised: November 12, 2021
Accepted: March 7, 2022
Published: March 30, 2022

REFERENCES

Abbott, L.F., and Blum, K.I. (1996). Functional significance of long-term potentiation for sequence learning and prediction. Cereb. Cortex *6*, 406–416.

Abeles, M. (1991). Corticonics: Neural Circuits of the Cerebral Cortex (Cambridge University Press).

Ackman, J.B., Burbridge, T.J., and Crair, M.C. (2012). Retinal waves coordinate patterned activity throughout the developing visual system. Nature *490*, 219–225.

Averbeck, B.B., Latham, P.E., and Pouget, A. (2006). Neural correlations, population coding and computation. Nat. Rev. Neurosci. *7*, 358–366.

Bair, W., Cavanaugh, J.R., Smith, M.A., and Movshon, J.A. (2002). The timing of response onset and offset in macaque visual neurons. J. Neurosci. *22*, 3189–3205.

Bashivan, P., Kar, K., and DiCarlo, J.J. (2019). Neural population control via deep image synthesis. Science *364*, eaav9436.

Berkes, P., Orbán, G., Lengyel, M., and Fiser, J. (2011). Spontaneous cortical activity reveals hallmarks of an optimal internal model of the environment. Science *331*, 83–87.

Bi, G.-Q., and Poo, M.-M. (2001). Synaptic modification by correlated activity: Hebb's postulate revisited. Annu. Rev. Neurosci. *24*, 139–166.

Branco, T., Clark, B.A., and Häusser, M. (2010). Dendritic discrimination of temporal input sequences in cortical neurons. Science *329*, 1671–1675.

Brea, J., Senn, W., and Pfister, J.-P. (2011). Sequence learning with hidden units in spiking neural networks. In Advances in Neural Information Processing Systems, pp. 1422–1430.

Brewster, S.D. (1844). A notice explaining the cause of an optical phenomenon observed by the Rev. W. Selwyn. Report of the Fourteenth Meeting of the British Association for the Advancement of Science.

Carr, M.F., Jadhav, S.P., and Frank, L.M. (2011). Hippocampal replay in the awake state: a potential substrate for memory consolidation and retrieval. Nat. Neurosci. *14*, 147–153.

Chiu, C., and Weliky, M. (2001). Spontaneous activity in developing ferret visual cortex *in vivo*. J. Neurosci. *21*, 8906–8914.

Churchland, M.M., Cunningham, J.P., Kaufman, M.T., Foster, J.D., Nuyujukian, P., Ryu, S.I., and Shenoy, K.V. (2012). Neural population dynamics during reaching. Nature *487*, 51–56.

Clopath, C., Büsing, L., Vasilaki, E., and Gerstner, W. (2010). Connectivity reflects coding: a model of voltage-based stdp with homeostasis. Nat. Neurosci. *13*, 344–352.

Cossell, L., Iacaruso, M.F., Muir, D.R., Houlton, R., Sader, E.N., Ko, H., Hofer, S.B., and Mrsic-Flogel, T.D. (2015). Functional organization of excitatory synaptic strength in primary visual cortex. Nature *518*, 399–403.

Dave, A.S., and Margoliash, D. (2000). Song replay during sleep and computational rules for sensorimotor vocal learning. Science *290*, 812–816.

Davidson, T.J., Kloosterman, F., and Wilson, M.A. (2009). Hippocampal replay of extended experience. Neuron *63*, 497–507.

Dayan, P., and Abbott, L.F. (2001). Theoretical Neuroscience: Computational and Mathematical Modeling of Neural Systems (MIT Press).

Diba, K., and Buzsáki, G. (2007). Forward and reverse hippocampal place-cell sequences during ripples. Nat. Neurosci. *10*, 1241–1242.

Eccles, J.C. (1986). Chemical transmission and Dale's principle. Prog. Brain Res. *68*, 3–13.

Ekman, M., Kok, P., and de Lange, F.P. (2017). Time-compressed preplay of anticipated events in human primary visual cortex. Nat. Commun. *8*, 15276.

Euler, T., and Masland, R.H. (2000). Light-evoked responses of bipolar cells in a mammalian retina. J. Neurophysiol. *83*, 1817–1829.

Fee, M.S., Kozhevnikov, A.A., and Hahnloser, R.H. (2004). Neural mechanisms of vocal sequence generation in the songbird. Ann. N. Y. Acad. Sci. *1016*, 153–170.

Fiete, I.R., Senn, W., Wang, C.Z., and Hahnloser, R.H. (2010). Spike-time-dependent plasticity and heterosynaptic competition organize networks to produce long scale-free sequences of neural activity. Neuron *65*, 563–576.

Fiser, J., Chiu, C., and Weliky, M. (2004). Small modulation of ongoing cortical dynamics by sensory input during natural vision. Nature *431*, 573–578.

Ganguli, S., Bisley, J.W., Roitman, J.D., Shadlen, M.N., Goldberg, M.E., and Miller, K.D. (2008). One-dimensional dynamics of attention and decision making in LIP. Neuron *58*, 15–25.

Gerstner, W., Kempter, R., Van Hemmen, J.L., and Wagner, H. (1996). A neuronal learning rule for sub-millisecond temporal coding. Nature *383*, 76–81.

Goldman, M.S. (2009). Memory without feedback in a neural network. Neuron *61*, 621–634.

Grosof, D.H., Shapley, R.M., and Hawken, M.J. (1993). Macaque VI neurons can signal 'illusory' contours. Nature *365*, 550–552.

Gupta, A.S., van der Meer, M.A., Touretzky, D.S., and Redish, A.D. (2010). Hippocampal replay is not a simple function of experience. Neuron *65*, 695–705.

Gütig, R., and Sompolinsky, H. (2006). The tempotron: a neuron that learns spike timing–based decisions. Nat. Neurosci. *9*, 420–428.

Hebb, D.O. (1949). The Organization of Behavior: A Neuropsychological Theory (Wiley).

Hennequin, G., Agnes, E.J., and Vogels, T.P. (2017). Inhibitory plasticity: balance, control, and codependence. Annu. Rev. Neurosci. *40*, 557–579.

Hoffman, K.L., and McNaughton, B.L. (2002). Coordinated reactivation of distributed memory traces in primate neocortex. Science *297*, 2070–2073.

Horn, R.A., and Johnson, C.R. (2012). Matrix analysis (Cambridge University Press).

Ikegaya, Y., Aaron, G., Cossart, R., Aronov, D., Lampl, I., Ferster, D., and Yuste, R. (2004). Synfire chains and cortical songs: temporal modules of cortical activity. Science *304*, 559–564.

Ji, D., and Wilson, M.A. (2007). Coordinated memory replay in the visual cortex and hippocampus during sleep. Nat. Neurosci. *10*, 100–107.

Kandel, E.R., Schwartz, J.H., Jessell, T.M., Siegelbaum, S., and Hudspeth, A. (2013). Principles of Neural Science, Fifth Edition (McGraw-Hill).

Kanizsa, G. (1955). Margini quasi-percettivi in campi con stimolazione omogenea. Riv. Psicol. *49*, 7–30.

Kempter, R., Gerstner, W., and Van Hemmen, J.L. (1999). Hebbian learning and spiking neurons. Phys. Rev. E *59*, 4498–4514.

Ko, H., Hofer, S.B., Pichler, B., Buchanan, K.A., Sjöström, P.J., and Mrsic-Flogel, T.D. (2011). Functional specificity of local synaptic connections in neocortical networks. Nature *473*, 87–91.

Lee, D., Seo, H., and Jung, M.W. (2012). Neural basis of reinforcement learning and decision making. Annu. Rev. Neurosci. *35*, 287–308.

Lengyel, M., Kwag, J., Paulsen, O., and Dayan, P. (2005). Matching storage and recall: hippocampal spike timing–dependent plasticity and phase response curves. Nat. Neurosci. *8*, 1677–1683.

Liu, W.W., and Wilson, R.I. (2013). Glutamate is an inhibitory neurotransmitter in the drosophila olfactory system. Proc. Natl. Acad. Sci. USA *110*, 10294–10299.

Machens, C.K., Romo, R., and Brody, C.D. (2010). Functional, but not anatomical, separation of "what" and "when" in prefrontal cortex. J. Neurosci. *30*, 350–360.

Mackevicius, E.L., Bahle, A.H., Williams, A.H., Gu, S., Denisenko, N.I., Goldman, M.S., and Fee, M.S. (2019). Unsupervised discovery of temporal sequences in high-dimensional datasets, with applications to neuroscience. eLife *8*, e38471.

Mastronarde, D.N. (1989). Correlated firing of retinal ganglion cells. Trends Neurosci. *12*, 75–80.

Mattar, M.G., and Daw, N.D. (2018). Prioritized memory access explains planning and hippocampal replay. Nat. Neurosci. *21*, 1609–1617.

Mazzucato, L., Fontanini, A., and La Camera, G. (2015). Dynamics of multistable states during ongoing and evoked cortical activity. J. Neurosci. *35*, 8214–8231.

McNaughton, B.L., Barnes, C.A., and O'Keefe, J. (1983). The contributions of position, direction, and velocity to single unit activity in the hippocampus of freely-moving rats. Exp. Brain Res. *52*, 41–49.

Morrison, A., Diesmann, M., and Gerstner, W. (2008). Phenomenological models of synaptic plasticity based on spike timing. Biol. Cybern. *98*, 459–478.

Nádasdy, Z., Hirase, H., Czurkó, A., Csicsvari, J., and Buzsáki, G. (1999). Replay and time compression of recurring spike sequences in the hippocampus. J. Neurosci. *19*, 9497–9507.

Nowak, L.G., Munk, M.H., Girard, P., and Bullier, J. (1995). Visual latencies in areas v1 and v2 of the macaque monkey. Vis. Neurosci. *12*, 371–384.

Okubo, T.S., Mackevicius, E.L., Payne, H.L., Lynch, G.F., and Fee, M.S. (2015). Growth and splitting of neural sequences in songbird vocal development. Nature *528*, 352–357.

Ólafsdóttir, H.F., Bush, D., and Barry, C. (2018). The role of hippocampal replay in memory and planning. Curr. Biol. *28*, R37–R50.

Orhan, A.E., and Ma, W.J. (2019). A diverse range of factors affect the nature of neural representations underlying short-term memory. Nat. Neurosci. *22*, 275–283.

Pfeiffer, B.E., and Foster, D.J. (2013). Hippocampal place-cell sequences depict future paths to remembered goals. Nature *497*, 74–79.

Raimondo, J.V., Markram, H., and Akerman, C.J. (2012). Short-term ionic plasticity at GABAergic synapses. Front. Synaptic Neurosci. *4*, 5.

Renart, A., De La Rocha, J., Bartho, P., Hollender, L., Parga, N., Reyes, A., and Harris, K.D. (2010). The asynchronous state in cortical circuits. Science *327*, 587–590.

Rossi, A.F., Rittenhouse, C.D., and Paradiso, M.A. (1996). The representation of brightness in primary visual cortex. Science *273*, 1104–1107.

Rutten, V., Bernacchia, A., and Hennequin, G. (2020a). Sequential components analysis. In Cosyne (Denver, CO), T-22.

Rutten, V., Bernacchia, A., Sahani, M., and Hennequin, G. (2020b). Non-reversible Gaussian processes for identifying latent dynamical structure in neural data. Adv. Neural Inf. Process. Syst. *33*, 9622–9632.

Sengpiel, F., and Kind, P.C. (2002). The role of activity in development of the visual system. Curr. Biol. *12*, R818–R826.

SenGupta, A.M., and Mitra, P.P. (1999). Distributions of singular values for some random matrices. Phys. Rev. E Stat. Phys. Plasmas Fluids Relat. Interdiscip. Topics *60*, 3389–3392.

Seung, H.S. (1996). How the brain keeps the eyes still. Proc. Natl. Acad. Sci. USA *93*, 13339–13344.

Skaggs, W.E., and McNaughton, B.L. (1996). Replay of neuronal firing sequences in rat hippocampus during sleep following spatial experience. Science *271*, 1870–1873.

Spitzer, N.C. (2012). Activity-dependent neurotransmitter respecification. Nat. Rev. Neurosci. *13*, 94–106.

Vogels, T.P., Froemke, R.C., Doyon, N., Gilson, M., Haas, J.S., Liu, R., Maffei, A., Miller, P., Wierenga, C.J., Woodin, M.A., et al. (2013). Inhibitory synaptic plasticity: spike timing-dependence and putative network function. Front. Neural Circuits *7*, 119.

Walker, E.Y., Sinz, F.H., Cobos, E., Muhammad, T., Froudarakis, E., Fahey, P.G., Ecker, A.S., Reimer, J., Pitkow, X., and Tolias, A.S. (2019). Inception loops discover what excites neurons most using deep predictive models. Nat. Neurosci. *22*, 2060–2065.

Wehr, M., and Laurent, G. (1996). Odour encoding by temporal sequences of firing in oscillating neural assemblies. Nature *384*, 162–166.

Xu, S., Jiang, W., Poo, M.-M., and Dan, Y. (2012). Activity recall in a visual cortical ensemble. Nat. Neurosci. *15*, 449–455, S1.

Zhou, S., Masmanidis, S.C., and Buonomano, D.V. (2020). Neural sequences as an optimal dynamical regime for the readout of time. Neuron *108*, 651–658.e5.

Znamenskiy, P., Kim, M.-H., Muir, D.R., Iacaruso, M.F., Hofer, S.B., and Mrsic-Flogel, T.D. (2018). Functional selectivity and specific connectivity of inhibitory neurons in primary visual cortex. Preprint at bioRxiv. https://doi.org/10.1101/294835.

CellPress
OPEN ACCESS

STAR★METHODS

KEY RESOURCES TABLE

REAGENT or RESOURCE	SOURCE	IDENTIFIER
Deposited data		
Raw and analyzed data	This paper	https://doi.org/10.5281/zenodo.6124683
Experimental models: Organisms/strains		
13 male sable ferrets (*Mustela putorius furo*) at different stages of visual development ranging from P44 to P151.	The Fiser Laboratory	Wild type
Software and algorithms		
MATLAB	Mathworks	R2018b (9.5.0.944444)
Code for computing sequentiality	This paper	https://doi.org/10.5281/zenodo.6124314

RESOURCE AVAILABILITY

Lead contact

Further information and requests for resources should be directed to and will be fulfilled by the lead contact, Alberto Bernacchia (ab2349@cam.ac.uk).

Materials availability

This study did not generate new unique reagents.

Data and code availability

- All original data has been deposited at https://doi.org/10.5281/zenodo.6124683 and is publicly available as of the date of publication. The DOI is listed in the key resources table.
- All original code has been deposited at https://doi.org/10.5281/zenodo.6124314 and is publicly available as of the date of publication. The DOI is listed in the key resources table.
- Any additional information required to reanalyze the data reported in this paper is available from the lead contact upon request.

EXPERIMENTAL MODEL AND SUBJECT DETAILS

For the experiments reported in Figures 5 and S5, 13 male sable ferrets (*Mustela putorius furo*) were used at different stages of visual development ranging from P44 to P151. All experimental procedures and handling of animals were approved by the University of Rochester Committee on Animal Research and performed in compliance with National Institutes of Health guidelines.

METHOD DETAILS

Animal preparation and data acquisition

The details of the procedures have been described in detail previously (Fiser et al., 2004; Chiu and Weliky, 2001; Berkes et al., 2011). Briefly, a linear array of 16 electrodes, spaced by 200 micrometers, was implanted in layer 2-3 of the primary visual cortex (V1) under isoflurane anesthesia. The electrodes typically provided clear multi-unit (and occasionally single-unit) signal on each channel. The signal was pre-processed by band-pass filtering (600-6000 Hz) and digitized at 10 kHz. Spike discrimination was performed offline by manually setting a separate voltage threshold for each electrode. Stable recordings were maintained for 8-12 hours.

Visual stimulation

Shortly after recovery from surgery, neural activity in response to different stimulus ensembles was recorded in awake animals. Animals rested on a padded platform with their head fixed to a rigid metal post and were free to make natural eye movements. Stimuli were displayed on a 4 × 3 feet back-projection screen at a distance of 30 cm from the head, covering 130 × 100 degrees of visual angle. The screen resolution was 1024 × 768 pixels, with a refresh rate of 75 Hz.

Animals were presented with two stimulus conditions to which responses were analyzed here:

- Movie evoked (natural) activity: Stimuli consisted of a movie (the trailer for the film The Matrix), presented at a resolution of 720 × 480 pixels and a frame rate of 24 Hz. This stimulus ensemble is meant to capture the distribution of the statistics of natural stimuli at the level of the simple visual elements encoded by V1 neurons.
- Noise evoked (artificial) activity: Random noise was generated as a grid of black and white squares, each occupying 5 × 5 degrees of visual angle. A new pattern was generated at random at each screen refresh, with white squares appearing independently with probability 1/4.

Recordings were also performed in two other stimulus conditions, using drifting gratings and complete darkness (spontaneous activity), respectively. We reported analyses of these conditions elsewhere (Berkes et al., 2011), but they were not analyzed here as our theory of sequentiality did not apply to them: drifting gratings themselves were already sequential by construction, making it impossible to identify sequentiality in responses as spurious, and we had no control over the input reaching visual cortex in the spontaneous activity condition.

Recordings with different stimulus ensembles were performed in interleaved trials of 100 sec, 15 trials for each ensemble, for a total of 25 minutes of recording in each condition. Figure 5F presents an analysis using all 25 mins of each of movie-evoked (natural) and noise-evoked (artificial) recordings. Figures 5G and S5 present analyses using the first and second halves of these ‘concatenated’ 25 minutes of each condition.

Neural circuit model

Our model is composed of a set of N variables v_i, $i = 1,\ldots,N$, each variable describing the activation of a given neuron, e.g. the membrane potential. The membrane potential evolves in time according to a firing rate model, i.e. a set of N coupled first-order differential equations (see also Equation 1, repeated here for convenience):

$$\tau\frac{dv_i}{dt} = -v_i(t) + \sum_{j=1}^{N} W_{ij}\, f[v_j(t)] + \xi_i(t) \qquad \text{(Equation 5)}$$

where W is the synaptic matrix, $f[\cdot]$ is a function expressing the transformation of membrane potentials into momentary firing rates, ξ_i is the external input and τ is the membrane time constant. Although network dynamics were purely rate-based (Equation 5), for illustrative purposes only, we also generated spikes based on neural firing rates in Figure 1C. Specifically, for each neuron i, action potentials were generated according to an inhomogeneous Poisson process with rate $f[v_i(t)]$. We also used a mean-field approach to simulate the average effects of STDP on synaptic weights (Figure 4), which again was only based on firing rates (see below, A model of spike timing-dependent plasticity). Similarly, all analyses of simulated data (e.g. covariances, sequentiality) were based on the firing rates, not the spikes. Thus, the spikes we generated did not enter into any further simulations or computations. Nevertheless, all our analyses using firing rates are equivalent to analyzing spiking activity over sufficiently long recordings in which the effects of (Poisson) spiking average out.

We assume that the external input $\xi_i(t)$ (the last term in Equation 5) is a stationary Gaussian stochastic process, with a mean μ (that is the same for all cells) and a "separable" covariance function that factorizes across space and time:

$$C_{ij}^{\text{in}}(s) = \langle \delta\xi_i(t+s)\ \delta\xi_j(t)\rangle = \Sigma_{ij}^{\text{in}} g(s) \qquad \text{(Equation 6)}$$

where angular brackets $\langle\cdot\rangle$ denote averaging over different realizations of the stochastic process $\xi_i(t)$, and $\delta\cdot$ denotes deviations from mean. Equation 6 defines the matrix of input spatial covariances Σ^{in}, a positive definite matrix that does not depend on s, as well as the temporal covariance function $g(s)$, a scalar and time-symmetric function obeying $g(-s) = g(s)$. In all simulations we use

$$g(s) = e^{-|s|/\tau_{\text{in}}}. \qquad \text{(Equation 7)}$$

We generate Gaussian noise with such exponentially decaying correlations (Ornstein-Uhlenbeck process) by filtering white noise with a first-order low-pass filter with characteristic timescale τ_{in}. The values of the synaptic matrix W and spatial covariance matrix Σ^{in} vary across simulations, and are described in Parameter values along with the values of all other parameters. We simulate the temporal evolution of v_i in Equation 5 using the Euler method with step size dt, initial conditions $v_i = 0$, and a total number of time steps T.

Parameter values

In all simulations we set $dt = 1$ ms, $\tau = 20$ ms, $\tau_{\text{in}} = 15$ ms, $T = 10^5$, and $\mu = 0.1$. In Figures 1 and 3, we use rectified linear units (ReLU) as response functions for all neurons: $f[v] = \max(0, v)$ (also clipped at one), whose slope is adjusted in order to have an average population output of 0.1. In Figure 2 we use a linear response function, $f[v] = v$. In Figures 1, 2F–2H, and 3, we set $N = 50$, while $N = 2$ in Figures 2A–2D.

In Figures 1 and 3A, the input covariance is equal to $\Sigma_{ij}^{\text{in}} = 0.5\, s_i\, s_j$ for $i \neq j$, and $\Sigma_{ii}^{\text{in}} = s_i^2$, where s_i values are drawn independently from an exponential distribution with mean equal to one. This corresponds to an input with correlations fixed to 0.5 and random variances s_i^2 across neurons. In Figures 3B and 3C, the input covariance is equal to $\Sigma_{ij}^{\text{in}} = s_i\, s_j \exp(-|i-j|/10)$. This

corresponds to correlations that decay in space within a scale of 10 neurons, and again random variances s_i^2 across neurons. In Figure 2 the input covariance is equal to, respectively, $\Sigma^{in} = [25\ 0; 0\ 1]$ in panel A, and $\Sigma^{in} = [1\ 0.9; 0.9\ 1]$ in panels B and C. In Figure 2D we draw Σ^{in} from a Wishart distribution with mean equal to the 2×2 identity matrix and 2 degrees of freedom. In Figures 2F and 2G we set Σ^{in} as in Figure 1. In Figure 2H, we set it to the sum of (i) a uniform matrix with all elements set to the same c drawn uniformly between 0 and 0.2 (which determined the resulting fraction of negative covariances), and (ii) a random covariance matrix $\propto U\Lambda U^T$ where U is a random unitary matrix and Λ is a diagonal matrix of exponentially decaying eigenvalues $\Lambda_{ii} = \exp(-i/10)$.

In Figure 1 we draw W_{ij} independently from a Gaussian distribution with zero mean and variance α^2/N, and then we set $W \to (W + W^T)/(2\sqrt{2})$ in order to obtain a symmetric matrix with maximum eigenvalue $\simeq \alpha = 0.9$. In Figure 2 we set, respectively, $W = [0\ 0.5; 0.5\ 0]$ in panel A, $W = [-0.5\ 0; 0\ 0.5]$ in B, $W = [-0.5\ 0.4; -0.4\ 0.5]$ in C. In Figure 2D, the matrix W is drawn as for Figure 1. In Figures 2E and 3, we set $W_{ij} = \Sigma_{ij}^{in} D$, where D_j are randomly drawn $+1$ or -1 with equal probability, and we rescale the matrix in order to have a maximum eigenvalue $\alpha = 0.9$. In Figure 2F, W is set as in Figure 2E, but the $p\,N^2$ connections corresponding to the smallest values of $|W_{ij}|$ are set to zero, where p is the sparseness. In Figure 2G, W is set as in Figure 2E, but the Hebb part of the matrix is drawn from an inverse Wishart distribution with mean equal to Σ^{in} and a varying number of degrees of freedoms, simulating a varying degree of noise. In Figure S2A, we draw W_{ij} independently from a Gaussian distribution with zero mean and variance α^2/N, in order to obtain a random matrix with maximum real part of the eigenvalue $\simeq \alpha = 0.9$. In Figure S2B, we follow the same procedure as in Figure S2A, and then we set $W \to (W - W^T)/2\sqrt{2}$ in order to obtain an anti-symmetric matrix. In Figure S2C, we follow the same procedure as in Figure S2A, and then we set $W_{ij} \to |W_{ij}| \cdot \text{sign}\left[\sum_i W_{ij}\right]$ in order to obtain a Dale matrix.

In STDP experiments (Figure 4), we do not simulate the network dynamics (Equation 5) explicitly, and instead compute sequentiality from the weight matrix (and other parameters) analytically using the expressions in Equations S74, S77, and S78 (assuming linear network dynamics). For tracking changes in synaptic weights, we simulate the mean field dynamics of Equation S82, with the kernel of Equation S80. We use the following values of parameters for the kernel: $\tau_+ = \tau_- = 15$ ms, $a_+ = 0.4375/\tau_+$, $a_- = 0.25/\tau_-$. The timescale for the STDP dynamics of Equation S82 is set to $\tau_S = 10$ minutes. The integral in Equation S82 is computed assuming linear dynamics in the colored input case, as derived in Equation S90. In Figure 4B, the input covariance and initial synaptic matrix are the same as of Figure 1, while in Figure 4C the input covariance is the same as in Figure 3B, and the initial synaptic matrix is equal to the end result of Figure 4C. The Dale matrix D is fixed in all simulations as explained above.

QUANTIFICATION AND STATISTICAL ANALYSIS

Cross-covariance

The cross-covariance is calculated for either the input or output signal, $X_{j,t}$, relevant for a neural population. In Figures 1, 2, and 3 these are the inputs received by individual simulated neurons, $\xi_j(t)$, and their activities, $v_j(t)$ (Equation 1), respectively. In Figure 5, these are the pixels of the stimulus and the spiking responses of the experimentally recorded units, with action potentials smoothed using an exponential kernel of 10 ms width. (Methods for the acquisition of neural recordings are described in experimental model and subject details). In Figures 1, 3, and 5, the cross-covariance is computed using the standard formula

$$C_{jk}(s) = \frac{1}{T}\sum_t (X_{j,t+s} - \mu_j)\,(X_{k,t} - \mu_k) \qquad \text{(Equation 8)}$$

$$\mu_j = \frac{1}{T}\sum_t X_{j,t} \qquad \text{(Equation 9)}$$

where $X_{j,t}$ is the value of signal j at time t, μ_j is the mean of $X_{j,t}$ over time, and s is the time lag. The cross-covariance is calculated for all N^2 pairs of signals, and for a set of S uniformly spaced time lags ranging from $s = -200$ ms to $s = +200$ ms in 2 ms bins. The index t runs over all allowed time steps, typically the length of the time series minus the number of time lags. Each dataset (either a numerical simulation or a recording session) is divided into $M = 10$ epochs; cross-covariances are computed in each epoch and then averaged across epochs. This procedure reduced the effects of non-stationarities and enabled estimation of the noise floor in the singular values of the matrix C (see noise estimation).

In Figure 2, cross-covariances are computed using theoretical predictions of the linearized system, Equations S64–S68. We have always found a very good agreement between theoretical predictions and numerical simulations.

Sequentiality

Sequentiality is defined as the ratio of the Frobenius norm of C^{asy} and that of C^{sym}, where C^{asy} and C^{sym} are the anti-symmetric and symmetric parts of the cross-covariance matrix C, respectively:

$$\text{seq} = \frac{||C^{asy}||_F}{||C^{sym}||_F} = \sqrt{\frac{\sum_{s,j,k}[C_{jk}(s) - C_{jk}(-s)]^2}{\sum_{s,j,k}[C_{jk}(s) + C_{jk}(-s)]^2}} \qquad \text{(Equation 10)}$$

Sequentiality is non-negative, because it is the ratio of non-negative numbers. Moreover, it is clear from Equation 10 that sequentiality is equal to zero if, and only if, the cross-covariance is symmetric for all neuron pairs, i.e. $C_{jk}(s) = C_{jk}(-s)$. This corresponds to a time series in which the second-order statistics is exactly time-reversible. Finally, we prove in Math Note S1 that sequentiality never exceeds one (Theorem 1). It is possible to show that seq = 1 in the case of a perfect sequence, defined by

$$X_{j,t} = f_{(t-j) \bmod N} \qquad \text{(Equation 11)}$$

where f_t is an arbitrary function of time t, which is displaced by one time step from one neuron to the next with cyclic boundary conditions (Figure S1).

Since data is noisy, we designed a method for estimating sequentiality, by separating the symmetric, anti-symmetric and noise terms of the cross covariance. We first define the matrix Γ, equal to $C_{ij}(s)$ where each column contains the cross-covariances of all possible pairs of neurons for a specific time lag (e.g. Figures 1F and 1G):

$$\Gamma_{(i+jN),s} = C_{ij}(s) \qquad \text{(Equation 12)}$$

Thus, Γ is a matrix with N^2 rows and S columns, where each row corresponds to a given pair of neurons and each column corresponds to a given time lag. We prove in Theorem 2 that all right singular vectors of Γ (which have the interpretation of temporal components) are either symmetric or anti-symmetric in the lag s. Moreover, from the definition of the Frobenius norm, we have that

$$\text{seq} = \sqrt{\frac{\sum_{m \in \text{asy}} \sigma_m^2}{\sum_{m \in \text{sym}} \sigma_m^2}} \qquad \text{(Equation 13)}$$

where the index m runs over either the anti-symmetric (asy) or the symmetric (sym) singular vectors of Γ, associated with their corresponding singular values σ_m. Noise can be eliminated by dropping all singular values smaller than a given threshold, obtained by estimating the singular values of the noise (see below).

Noise estimation

We consider the problem of estimating the error made when measuring the singular values of a matrix (Horn and Johnson, 2012) estimated from noisy data. Let Γ denote our stochastic matrix estimate, composed of a signal part Γ_s and a noise part Γ_n

$$\Gamma = \Gamma_s + \Gamma_n \qquad \text{(Equation 14)}$$

We assume that Γ_s is fixed (ground truth), and the noise term Γ_n is normally distributed with zero mean and an arbitrary covariance matrix Σ, expressing covariances in terms of the vectorized matrix $\text{vec}(\Gamma_n)$. The distribution of singular values of Γ_n has been calculated analytically under some assumptions, e.g. when the covariance Σ has separable contributions for the rows and columns of Γ_n (see case (*ii*) in SenGupta and Mitra, 1999). For arbitrary covariance matrices, this distribution can be estimated from the data by resampling. We assume that the estimate Γ is obtained from M independent and identically distributed samples indexed by i, namely

$$\Gamma = \frac{1}{M}\sum_{i=1}^{M}\Gamma^{(i)} \qquad \text{(Equation 15)}$$

For example, the samples $\Gamma^{(i)}$ can be obtained by dividing the data into M equal epochs. These samples can then be used to estimate the noise. In particular, we denote by $\Gamma_n^{(i)}$ the estimation error associated with sample i:

$$\Gamma^{(i)} = \Gamma_s + \Gamma_n^{(i)} \qquad \text{(Equation 16)}$$

The error in our overall estimate is simply the mean of all these error terms:

$$\Gamma_n = \frac{1}{M}\sum_{i=1}^{M}\Gamma_n^{(i)} \qquad \text{(Equation 17)}$$

Assuming that each $\Gamma_n^{(i)}$ is also normally, independently and identically distributed with zero mean, then its covariance is equal to $\sqrt{M}\,\Sigma$. Therefore, the distribution of the singular values of Γ_n is equal to the distribution of the singular values of $\Gamma_n^{(i)}/\sqrt{M}$:

$$\sigma(\Gamma_n) \sim \sigma\left(\Gamma_n^{(i)} \Big/ \sqrt{M}\right) = \sigma\left(\Gamma_n^{(i)}\right) \Big/ \sqrt{M} \qquad \text{(Equation 18)}$$

CellPress
OPEN ACCESS

Noise samples are not directly available in data, but can be obtained by taking the difference of pairs of epochs (such that the common signal part cancels out), and rescaling by the appropriate factor:

$$\Delta\Gamma_{ij} = \Gamma^{(i)} - \Gamma^{(j)} = \Gamma_{\mathrm{n}}^{(i)} - \Gamma_{\mathrm{n}}^{(j)} \qquad \sigma(\Gamma_n) \sim \sigma(\Delta\Gamma_{ij})\Big/\sqrt{2M} \qquad \text{(Equation 19)}$$

Note that pairwise differences may be correlated, for example when using a single epoch of data to compute multiple pairs, but all possible $N(N-1)/2$ differences are expected to be distributed equally, and can all be used to estimate the mean and covariance of $\sigma(\Gamma_{\mathrm{n}})$. In Figures 1, 3, and 5 we use this method to compute the mean μ and standard deviation σ of the spectrum of singular values of the noise, and subsequently discard all singular values of Γ that fall below $\mu + 7\sigma$.

Data analysis and exclusion

As our measure of sequentiality is based on a ratio taking values between zero and one, we reported its logarithm in all analyses based on experimental data (Figures 5 and S5), and for statistical testing using (two-tailed paired) t-tests. For the logarithm to be well defined, we excluded from these analyses all animals whose raw sequentiality was zero in either condition due to limited sample size. When analyzing all of the data (Figure 5F), one animal had zero sequentiality in the natural condition, and the same animal and an additional animal had zero sequentiality in the artificial condition, leaving 11 animals in the analysis. (We confirmed that the results remained unchanged when performing a Wilcoxon signed-rank test on the raw sequentiality scores without excluding any of the animals, p = 0.0122.) When comparing two halves of the data in the artificial condition (Figure 5G), the same four animals had zero sequentiality in both halves, leaving 9 animals in the analysis. When comparing two halves of the data in the natural condition (Figure S5), three animals had zero sequentiality in each half, with an overlap of only one animal, leading to the exclusion of 5 animals, leaving 8 animals in the analysis.

Neuron, Volume *110*

Supplemental information

Adaptive erasure of spurious sequences in sensory cortical circuits

Alberto Bernacchia, József Fiser, Guillaume Hennequin, and Máté Lengyel

Adaptive erasure of spurious sequences in cortical circuits
SUPPLEMENTARY MATERIAL

Alberto Bernacchia[1,2*], József Fiser[3], Guillaume Hennequin[1,4], Máté Lengyel[1,3,4]

[1]Department of Engineering, University of Cambridge,
Cambridge CB2 1PZ, United Kingdom
[2]MediaTek Research, Building 2010, Cambourne Business Park
Cambourne CB23 6DW, United Kingdom
[3]Department of Cognitive Science, Central European University,
Budapest H-1051, Hungary
[4]Equal contribution.

*To whom correspondence should be addressed; E-mail: ab2349@cam.ac.uk.

Contents

Supplementary Math Note 1 Theory of sequentiality **1**
1.1 Bounds . . . 1
1.2 Symmetries . . . 3

Supplementary Math Note 2 Theory of time-reversible systems **3**
2.1 Summary of results . . . 3
2.2 Derivation of covariance functions . . . 4
2.3 Conditions for time reversibility . . . 5
2.4 Linear examples . . . 8

Supplementary Math Note 3 A model of spike timing-dependent plasticity **10**
3.1 Mean field dynamics . . . 10
3.2 Example 1: linear dynamics and white input . . . 11
3.3 Example 2: linear dynamics and colored input . . . 12

Supplementary Math Note 1 Theory of sequentiality

1.1 Bounds

Theorem 1. *Sequentiality is bounded between zero and one.*

Proof. Sequentiality is trivially non-negative, because it is the ratio of non-negative numbers, therefore we only have to prove that it is smaller than one. We will assume that the cross-covariance is computed for all possible values of time lag ($S = T$) and wrapped around in a circle (i.e. $C(T + s) = C(s)$). In particular, we assume that T is odd and consider time

lags between $-(T-1)/2$ and $(T-1)/2$. In practice, this assumption is inessential as long as the maximum time lag is large enough to cover the bulk of the cross-covariance.

In matrix form, sequentiality in Equation 10 can be rewritten as

$$\text{seq}^2 = \frac{\sum_s \text{Tr}\left\{[C(s) - C(-s)]\,[C(s) - C(-s)]^T\right\}}{\sum_s \text{Tr}\left\{[C(s) + C(-s)]\,[C(s) + C(-s)]^T\right\}} \tag{S1}$$

Using the property $C(-s) = C(s)^T$, this can be simplified to

$$\text{seq}^2 = \frac{\sum_s \text{Tr}\left[C(s)C(s)^T\right] - \sum_s \text{Tr}\left[C(s)^2\right]}{\sum_s \text{Tr}\left[C(s)C(s)^T\right] + \sum_s \text{Tr}\left[C(s)^2\right]} \tag{S2}$$

In order to prove that sequentiality never exceeds one, we have to prove that the numerator never exceeds the denominator. By inspection of Equation S2, this is true if and only if $\sum_s \text{Tr}\left[C(s)^2\right] \geq 0$. Note that $\text{Tr}\left[C(s)C(s)^T\right]$ is non-negative for all values of s since it is equal to the sum of squared elements of $C(s)$. However, the second term, $\text{Tr}\left[C(s)^2\right]$ may be negative in some cases. For example, if $C(s)$ is anti-symmetric for some values of s, then $\text{Tr}\left[C(s)^2\right]$ is negative. However, we show below that it is non-negative when summed over all values of s.

We define the cross spectral matrix as the discrete Fourier transform of the cross covariance

$$\hat{C}(\omega) = \frac{1}{\sqrt{T}} \sum_s e^{-2\pi i s\omega/T} C(s) \tag{S3}$$

Since $C(s)^T = C(-s)$, it is straightforward to show that the cross spectral matrix is Hermitian. It is also possible to show that $\hat{C}(\omega)$ is positive semi-definite, for all frequencies ω. We define the matrix $\Pi_{tt'} = \mathbb{E}[x_t x_{t'}] = C(t-t')$, for a scalar and stationary random process x, where $\mathbb{E}$ denotes expectation over its distribution. The matrix Π is positive semidefinite and circulant, and Fourier transforming along both time variables (t and t') we obtain $\hat{\Pi}_{\omega\omega'} = \delta_{\omega\omega'}\hat{C}(\omega)$. Since $\hat{\Pi}$ is positive semidefinite, $\hat{C}(\omega)$ must be non-negative. The extension to the multivariate case is straightforward, and shows that $\hat{C}(\omega)$ is positive semi-definite. Using Parseval's theorem, we have

$$\sum_s \text{Tr}\left[C(s)^2\right] = \sum_\omega \text{Tr}\left[\hat{C}(\omega)\overline{\hat{C}}(\omega)\right] \tag{S4}$$

Each element of the sum on the right hand side must be non-negative, because it is the trace of a product of two Hermitian positive semi-definite matrices (see Theorem 4.3.53 in Ref. 80). Using similar arguments, we find

$$\sum_s \text{Tr}\left[C(s)C(s)^T\right] = \sum_\omega \text{Tr}\left[\hat{C}(\omega)^2\right] \tag{S5}$$

Therefore, substituting these expressions into Equation S2, we find that sequentiality is equal to

$$\text{seq}^2 = \frac{\sum_\omega \text{Tr}\left[\hat{C}(\omega)^2\right] - \sum_\omega \text{Tr}\left[\hat{C}(\omega)\overline{\hat{C}}(\omega)\right]}{\sum_\omega \text{Tr}\left[\hat{C}(\omega)^2\right] + \sum_\omega \text{Tr}\left[\hat{C}(\omega)\overline{\hat{C}}(\omega)\right]} \tag{S6}$$

which never exceeds one since all sums are positive.

□

1.2 Symmetries

Theorem 2. *When its singular values are distinct, all singular vectors of the reshaped cross-covariance* Γ *in Equation 12 are either symmetric or anti-symmetric.*

Proof. Let K be the commutation matrix, of size $N^2 \times N^2$, operating in the vector space of neuron pairs, that replaces the $(i + jN)$ pair with the $(j + iN)$ pair. This corresponds to transposing the original cross covariance matrix, replacing ij with ji, but operating on the vector space of neuron pairs. We also define R as the reflection matrix that replaces the time lag s with the time lag $-s$. This corresponds to flipping the time lag vector. Using the identity $C_{ij}(s) = C_{ji}(-s)$, it is straightforward to note that transposing space or transposing time are two equivalent operations, i.e.

$$K\Gamma R^T = \Gamma \tag{S7}$$

Therefore the reshaped cross-covariance matrix is invariant upon the simultaneous action of K and R. We denote the SVD of Γ by

$$\Gamma = USV^T \tag{S8}$$

where S is a diagonal matrix containing the singular values of Γ, and the columns of matrix U (resp. V) are the (orthogonal) left (resp. right) singular vectors of Γ. Then, Equations S7 and S8 imply that

$$(KU)S(RV)^T = USV^T \tag{S9}$$

Since the permutation matrices K and R are orthogonal, the matrices in round brackets are also orthogonal. Note that the SVD of a matrix is unique, provided that singular values are distinct, and except for a simultaneous sign change of the same column of U and V. Therefore, we must have that

$$KU = U\pm \tag{S10}$$

$$RV = V\pm \tag{S11}$$

where $\pm$ is a diagonal matrix composed of $+1$ and -1 elements only. Note that signs apply separately to different columns, but the sign of a given column of U and V matches. From the meaning of the K and R as spatial transpose and time-reversal respectively, these equations imply that each pair singular vectors (left and right) are either both symmetric or both anti-symmetric in space (left) and time (right). This, in turn, means that each singular value can be unequivocally labelled as "symmetric" or "anti-symmetric", thus justifying the alternative expression of sequentiality given by Equation 13. □

Supplementary Math Note 2 Theory of time-reversible systems

2.1 Summary of results

We are interested in determining under which conditions the activity produced by the dynamical system in Equation 5 is time-reversible. Our main assumptions are: 1) that the system relaxes to a stationary state, and 2) that all $v_i(t)$ and $\xi_j(t)$ are jointly Gaussian. These assumptions are exactly satisfied only when $f_j[v] \propto v$ (linear response functions), but they still hold approximately for weakly nonlinear transfer functions, especially if N is large. We show in Section 2.3 that the following synaptic matrix guarantees (is a sufficient condition for) time reversibility:

$$W_{ij} = H_{ij}D_j \tag{S12}$$

where H — which we refer to as the "Hebb" part — is a matrix that must satisfy the conditions detailed in Section 2.3 and D — the "Dale" part (see below) — is some vector of presynaptic factors. A simple example of a "Hebb" factor H that guarantees time reversibility is the input covariance

$$H_{ij} = \Sigma^{\text{in}}_{ij} \tag{S13}$$

Another possible form is

$$H_{ij} = \int_{-\infty}^{+\infty} ds\, C_{ij}^{\text{out}}(s) k(s) \tag{S14}$$

where $k(s)$ is an arbitrary scalar kernel function and Σ^{out} is the output covariance matrix,

$$C_{ij}^{\text{out}}(s) = \langle \delta v_i(t+s) \delta v_j(t) \rangle \tag{S15}$$

Note that C^{out} depends on H itself, therefore Equation S14 gives an implicit formula that must be solved for H. The proportionality of synaptic strengths to the covariance of neural activity is consistent with empirical observations Cossell et al. (2015). Furthermore, by interpreting $k(s)$ as an STDP kernel, we show in Section 3.1 that this matrix represents a fixed point of STDP dynamics, which can be simulated in order to solve Equation S14. Finally, assuming that all elements of the Hebb component H are positive, then neuron j is excitatory (i.e. all its outgoing weights are positive) if $D_j \geq 0$; it is inhibitory otherwise. This justifies the interpretation of D as a "Dale" component. The derivation of Equations S12 to S14 is provided in Section 2.3 (see derivations leading up to Equations S47 and S54).

2.2 Derivation of covariance functions

In this section we derive formulas for the covariance functions, under the assumptions of stationarity and Gaussianity. For convenience of notation, we rewrite Equation 5 in matrix form as

$$\tau \frac{dv}{dt} = -v(t) + W\, f\left[v(t)\right] + \xi(t) \tag{S16}$$

We assume that the system relaxes to a stationary state, and define the following cross-covariance matrices

$$C^{\text{in}}(s) = \left\langle \delta\xi(t+s) \delta\xi(t)^T \right\rangle \tag{S17}$$

$$C^{\text{out}}(s) = \left\langle \delta v(t+s) \delta v(t)^T \right\rangle \tag{S18}$$

$$C^{\text{io}}(s) = \left\langle \delta v(t+s) \delta\xi(t)^T \right\rangle \tag{S19}$$

where angular brackets correspond to the operation of averaging over different realizations of the stochastic process $\xi(t)$. Note that stationarity implies that $C^{\text{in}}(-s)^T = C^{\text{in}}(s)$, and $C^{\text{out}}(-s)^T = C^{\text{out}}(s)$. The following theorem provides an expression for these cross-covariance matrices.

Theorem 3. *Given the dynamical system described by Equation S16, if the system is stationary and the variables (v, ξ) are jointly Gaussian, then the cross-covariance matrices satisfy the following expressions:*

$$(i\omega\tau - J)\, \hat{C}^{\text{io}}(\omega) = \hat{C}^{\text{in}}(\omega) \tag{S20}$$

$$(i\omega\tau - J)\, \hat{C}^{\text{out}}(\omega) = \hat{C}^{\text{io}}(-\omega)^T \tag{S21}$$

where

$$J = W\Phi - I \tag{S22}$$

with a diagonal matrix Φ with elements

$$\Phi_{ii} = \langle f_i' \left[v_i\right] \rangle \,. \tag{S23}$$

Here, the Fourier transform and inverse transform are defined by

$$\hat{C}(\omega) = \frac{1}{\sqrt{2\pi}} \int_{-\infty}^{+\infty} ds\, C(s) e^{-i\omega s} \tag{S24}$$

$$C(s) = \frac{1}{\sqrt{2\pi}} \int_{-\infty}^{+\infty} d\omega\, \hat{C}(\omega) e^{i\omega s}. \tag{S25}$$

Proof. We first derive the differential equations that govern the evolution of the covariance matrices. To do this, we shift temporally and multiply Equation S16 by, respectively, the transposed input and output deviations

$$\tau \frac{dv(t+s)}{ds}\delta\xi(t)^T = -v(t+s)\delta\xi(t)^T + W\, f\left[v(t+s)\right]\delta\xi(t)^T + \xi(t+s)\delta\xi(t)^T \tag{S26}$$

$$\tau \frac{dv(t+s)}{ds}\delta v(t)^T = -v(t+s)\delta v(t)^T + W\, f\left[v(t+s)\right]\delta v(t)^T + \xi(t+s)\delta v(t)^T \tag{S27}$$

In order to average these equations, we use the assumption that $\{\xi(t), v(s)\}$ are jointly distributed according to a multivariate Gaussian, along with Bussgang's theorem, to obtain[1]

$$\left\langle f\left[v(t+s)\right]\delta\xi(t)^T\right\rangle = \Phi\left\langle \delta v(t+s)\delta\xi(t)^T\right\rangle = \Phi C^{\text{io}}(s) \tag{S28}$$

$$\left\langle f\left[v(t+s)\right]\delta v(t)^T\right\rangle = \Phi\left\langle \delta v(t+s)\delta v(t)^T\right\rangle = \Phi C^{\text{out}}(s) \tag{S29}$$

where the diagonal matrix Φ is given in the theorem statement. Note that $\Phi = I$ when the transfer function is linear. Defining the matrix $J = W\Phi - 1$, and averaging Equations S26 and S27, we obtain the following differential equations for the covariance matrices:

$$\tau \frac{d}{ds}C^{\text{io}}(s) = JC^{\text{io}}(s) + C^{\text{in}}(s) \tag{S30}$$

$$\tau \frac{d}{ds}C^{\text{out}}(s) = JC^{\text{out}}(s) + C^{\text{io}}(-s)^T \tag{S31}$$

The theorem follows by applying the Fourier transform to Equations S30 and S31. □

Concerning implementation, we first note that $\hat{C}^{\text{in}}(-\omega)^T = \hat{C}^{\text{in}}(\omega)$, and $\hat{C}^{\text{out}}(-\omega)^T = \hat{C}^{\text{out}}(\omega)$; however, in general $\hat{C}^{\text{io}}(-\omega) \neq \hat{C}^{\text{io}}(\omega)$. Furthermore, $\hat{C}^{\text{io}}(\omega)$ can be eliminated from Equations S20 and S21, to obtain

$$\left(i\omega\tau - J\right)\hat{C}^{\text{out}}(\omega)\left(-i\omega\tau - J^T\right) = \hat{C}^{\text{in}}(\omega) \tag{S32}$$

This equation is nonlinear, since the matrix Φ, and therefore J, depends on the diagonal of $C^{\text{out}}(0)$. However, J does not depend on ω; thus, we can alternate between (i) solving a *linear* equation for $\hat{C}^{\text{out}}$ given fixed J, and (ii) fixing $\hat{C}^{\text{out}}$ and using Equation S22 to obtain J. This will yield a self-consistent solution.

2.3 Conditions for time reversibility

In this section, we turn to the problem of finding necessary and sufficient conditions for time reversibility. Recall that we assumed that the input covariance is symmetric in time (cf. Equation 6 and below):

$$C^{\text{in}}(-s) = C^{\text{in}}(s) \tag{S33}$$

By definition, the network output is said to be time-reversible if the same type of equality holds for network activity as well:

$$C^{\text{out}}(-s) = C^{\text{out}}(s) \tag{S34}$$

Therefore, the goal is to find conditions under which Equation S33 implies Equation S34. We stress that time reversibility implies that the joint probability of two states, at two different time points, is symmetric. Because of the Gaussianity assumption, this reduces to time reversibility of the covariance, Equation S34, since higher order statistics do not contribute (and the mean is constant due to stationarity). We prove the following.

[1] For a Gaussian distribution $P(\boldsymbol{x})$ of zero mean and covariance Σ, the following identity holds: $\Sigma\boldsymbol{\nabla}P = -\boldsymbol{x}P$, therefore $\langle f(x_1)x_2\rangle = \int dx_1 \int dx_2 f(x_1)x_2 P(x_1,x_2) = -\int dx_1 \int dx_2 f(x_1)\left(\Sigma_{21}\partial_{x_1} + \Sigma_{22}\partial_{x_2}\right)P(x_1,x_2) = -\Sigma_{21}\int dx_1 f(x_1)\partial_{x_1}P(x_1) - \Sigma_{22}\int dx_1 f(x_1)\int dx_2 \partial_{x_2}P(x_1,x_2)$. Integrating by parts, the second term is zero while the first is equal to $\Sigma_{21}\langle f'(x_1)\rangle$.

Theorem 4. *Given time-reversible input, the output is time reversible if and only if any one of the following equalities holds:*

$$JC^{\text{out}}(s) = C^{\text{out}}(s)J^T \qquad \textit{for all } s \tag{S35}$$

$$JC^{\text{io}}(s) = C^{\text{io}}(s)J^T \qquad \textit{for all } s \tag{S36}$$

$$JC^{\text{in}}(s) = C^{\text{in}}(s)J^T \qquad \textit{for all } s \tag{S37}$$

Proof. Due to the stationarity assumption, symmetry in time implies symmetry in space, since as noted above we must have that $C^{\text{in}}(-s)^T = C^{\text{in}}(s)$, which is equivalent to $\hat{C}^{\text{in}}(-\omega)^T = \hat{C}^{\text{in}}(\omega)$ in Fourier space. Therefore, the input covariance satisfies the following equalities:

$$C^{\text{in}}(s) = C^{\text{in}}(s)^T \tag{S38}$$

$$\hat{C}^{\text{in}}(\omega) = \hat{C}^{\text{in}}(\omega)^T \tag{S39}$$

Similarly, the output is time reversible if and only if either of the following equalities holds:

$$C^{\text{out}}(s) = C^{\text{out}}(s)^T \tag{S40}$$

$$\hat{C}^{\text{out}}(\omega) = \hat{C}^{\text{out}}(\omega)^T \tag{S41}$$

We focus on proving $\hat{C}^{\text{out}}(\omega)^T = \hat{C}^{\text{out}}(\omega)$. For brevity, we omit the dependence on ω, and we denote by $\hat{C}^{\text{out}}_{\text{sym}} = \hat{C}^{\text{out}} + \hat{C}^{out^T}$ the symmetric part of the covariance, and by $\hat{C}^{\text{out}}_{\text{asy}} = \hat{C}^{\text{out}} - \hat{C}^{\text{out}^T}$ its anti-symmetric part. Using Equations S32 and S33, we have that

$$i\omega\tau\left(J\hat{C}^{\text{out}}_{\text{sym}} - \hat{C}^{\text{out}}_{\text{sym}}J^T\right) + \omega^2\tau^2\hat{C}^{\text{out}}_{\text{asy}} + J\hat{C}^{\text{out}}_{\text{asy}}J^T = 0 \tag{S42}$$

Since this equation is linear in the covariance, the anti-symmetric part is zero if, and only if, the term in round brackets is zero. Therefore, a necessary and sufficient condition for time reversibility is

$$J\hat{C}^{\text{out}}(\omega) = \hat{C}^{\text{out}}(\omega)J^T \quad \text{for all } \omega \tag{S43}$$

Furthermore, it is straightforward to show that if J satisfies this equation, then all its powers satisfy the same equation, and therefore so does any analytic function of J. The converse is also true, since the inverse of any analytic function is also analytic. In particular, $i\omega\tau - J$ is analytic in J, and so is its inverse. Thus, from Equations S20 and S21, we have that

$$J\hat{C}^{\text{io}}(\omega) = \hat{C}^{\text{io}}(\omega)J^T \quad \text{for all } \omega \tag{S44}$$

$$J\hat{C}^{\text{in}}(\omega) = \hat{C}^{\text{in}}(\omega)J^T \quad \text{for all } \omega \tag{S45}$$

Therefore, any one of the three Equations S43 to S45 implies, and is implied by, any one of the other two. The theorem follows by applying the inverse Fourier transform to Equations S43 to S45. □

Consequence of Theorem 4 for "Hebb and Dale" conditions. Each one of Equations S35 to S37, on its own, is necessary and sufficient for time-reversibility of the output. Since the input covariance is given, while the other covariances must be determined, it is easiest to test for reversibility by checking whether Equation S37 holds. Using the separability of the input covariance (Equation 6), Equation S37 is rewritten as

$$J\Sigma^{\text{in}} - \Sigma^{\text{in}}J^T = 0 \tag{S46}$$

Using $J = W\Phi - I$, and noting that Φ is diagonal, it is possible to show that

$$W = \Sigma^{\text{in}}D \tag{S47}$$

satisfies Equation S37 for any arbitrary diagonal matrix D. This corresponds to Equation S13 in Section 2.1. However, other forms are also possible, including Equation S14, and we show in the following theorem how to derive them. We also note that, in the special case of a linear transfer function ($\Phi = I$), Equation S46 can be rewritten as

$$W\Sigma^{\text{in}} - \Sigma^{\text{in}}W^T = 0 \tag{S48}$$

Since sequentiality is zero when this equation holds, we use the norm of the left hand side of this expression as an approximation for *asym* in Equation 4 of the main text for $N = 2$ and for symmetric W and Σ. We verified that this approximation was accurate over a wide range of parameters in Figure 2C (Star Methods, "Parameter values").

Theorem 5. *We assume that the matrix J has distinct eigenvalues and the matrix Σ is non-singular. If a matrix J exists satisfying the equation*

$$J\Sigma = \Sigma J^T \tag{S49}$$

then Σ must be equal to

$$\Sigma = VEV^T \tag{S50}$$

where the columns of V are the eigenvectors of J, and E is a diagonal matrix. (Note that V needs not be an orthogonal basis, i.e. $V^TV \neq I$ in general). If we further assume that Σ is positive definite, then J must be equal to

$$J = \Sigma L \tag{S51}$$

where L is a symmetric matrix.

Proof. To verify this statement, note that Equation S49 implies $J = \Sigma J^T \Sigma^{-1}$. By writing the spectral decomposition of $J = V\Delta V^{-1}$, where V and Δ are, respectively, the eigenvectors and eigenvalues of J, we have that $\Delta = \left(V^T\Sigma^{-1}V\right)^{-1} \Delta \left(V^T\Sigma^{-1}V\right)$. Since the eigenvalues of J are distinct, $V^T\Sigma^{-1}V$ must be diagonal. Call this diagonal matrix E^{-1}, this gives us Equation S50. Moreover, Sylvester's law of inertial states that VEV^T have the same number of positive and negative eigenvalues as E does. Therefore, if we further assume that Σ is positive definite, then E must have positive elements. Under this assumption, we consider the unique singular value decomposition of $VE^{1/2} = U\Lambda^{1/2}U'$, where Λ is the diagonal matrix of singular values and U, U' are the orthogonal matrices of left and right singular vectors, respectively. It is straightforward to show that U and Λ are, respectively, the eigenvectors and eigenvalues of $\Sigma = U\Lambda U^T$. Finally, we rewrite J by substituting the expression of its eigenvectors, $V = U\Lambda^{1/2}U'E^{-1/2}$, in its spectral decomposition, $J = V\Delta V^{-1}$, and find

$$J = U\Lambda^{1/2}U'E^{-1/2}\Delta E^{1/2}U'^T\Lambda^{-1/2}U^T = U\Lambda U^T\left[U\Lambda^{-1/2}U'\Delta U'^T\Lambda^{-1/2}U^T\right] = \Sigma L \tag{S52}$$

where the last equality defines L. If J is unknown, while Σ is given, then U and Λ are given, while U' and Δ are arbitrary. Therefore, $U'\Delta U'^T$ is an arbitrary symmetric matrix, and so is L. □

In particular, Equation S50 allows us to conclude that, if a matrix J exists for which the system is time reversible, and Equation S35 holds, then we must have that $C^{\text{out}}(s) = VE^{\text{out}}(s)V^T$, where V are the eigenvectors of J and $E^{\text{out}}(s)$ is some diagonal matrix. Furthermore, since Equation S35 holds for all values of s, then any linear combination of different values of s can be taken. By taking an integral over different s values, weighted by a kernel $k(s)$, we have that $J\int ds\ k(s)C^{\text{out}}(s) = \int ds\ k(s)C^{\text{out}}(s)\ J^T$. Provided that the integral is positive definite, Theorem 5 implies that the synaptic matrix J must be equal to

$$J = \int_{-\infty}^{+\infty} ds\ k(s)C^{\text{out}}(s)\ L \tag{S53}$$

for some arbitrary scalar kernel $k(s)$ and symmetric matrix L. Conversely, we can set J by choosing any arbitrary symmetric matrix L in this expression, and this would still guarantee time reversibility. It is straightforward to show that L can be chosen in a way that

$$W = \int_{-\infty}^{+\infty} ds\ k(s)C^{\text{out}}(s)\ D \tag{S54}$$

where D is an arbitrary diagonal matrix. This corresponds to Equation S14 in Section 2.1. We will show in Section 3.1 that this expression can be interpreted as a fixed point of spike timing-dependent plasticity (STDP). As a final remark, we stress that no J may exist for which time reversibility holds (for example, for more complex types of input), in which case Equation S54 does not guarantee time reversibility.

2.4 Linear examples

In this section we consider the simple case in which the neural dynamics are linear, thus $\Phi = I$ and $J = W - I$ (see Section 2.2). In this case, the cross-covariances can be calculated analytically when the input has simple statistics.

First, we consider the case of white noise input, i.e. an input covariance of the form $C^{\text{in}}(s) = \Sigma^{\text{in}}\delta(s)$. We denote by $C^{\text{out}}(s)$ the output cross-covariance and by $\Sigma^{\text{out}} = C^{\text{out}}(0)$ the output covariance at zero time lag. The output covariance can be calculated from Equation S32, and is equal to

$$C^{\text{out}}(s) = \begin{cases} e^{(W-I)s/\tau}\ \Sigma^{\text{out}} & \text{for } s \geq 0 \\ \Sigma^{\text{out}}\ e^{-(W^T-I)s/\tau} & \text{for } s < 0 \end{cases} \tag{S55}$$

The covariance at zero time lag Σ^{out} is equal to

$$\Sigma^{\text{out}} = \frac{1}{\tau^2}\int_0^{+\infty} ds\ e^{(W-I)s/\tau}\ \Sigma^{\text{in}}\ e^{(W^T-I)s/\tau} \tag{S56}$$

Computing this integral is complicated, but Σ^{out} can be calculated instead by solving the Lyapunov equation

$$(W-I)\,\Sigma^{\text{out}} + \Sigma^{\text{out}}\left(W^T - I\right) + \Sigma^{\text{in}}/\tau = 0 \tag{S57}$$

We calculate the sequentiality using the definition, Equation 10, that we rewrite here substituting sums with integrals, since time is continuous in this case

$$\text{seq}^2 = \frac{\text{Tr}\int_{-\infty}^{+\infty} ds\ C^{\text{out}}(s)C^{\text{out}}(s)^T - \text{Tr}\int_{-\infty}^{+\infty} ds\ C^{\text{out}}(s)^2}{\text{Tr}\int_{-\infty}^{+\infty} ds\ C^{\text{out}}(s)C^{\text{out}}(s)^T + \text{Tr}\int_{-\infty}^{+\infty} ds\ C^{\text{out}}(s)^2} \tag{S58}$$

We substitute the expression for C^{out}, given by Equation S55, in the integrals and find that sequentiality can be expressed as

$$\text{seq}^2 = \frac{\text{Tr}\left(\mathcal{I}_1\right) - \text{Tr}\left(\mathcal{I}_2\Sigma^{\text{out}}\right)}{\text{Tr}\left(\mathcal{I}_1\right) + \text{Tr}\left(\mathcal{I}_2\Sigma^{\text{out}}\right)} \tag{S59}$$

Where the integrals $\mathcal{I}_1$ and $\mathcal{I}_2$ are equal to

$$\mathcal{I}_1 = \int_{-\infty}^{+\infty} ds\ e^{(W-I)s/\tau}\ \Sigma^{\text{out}2} e^{(W^T-I)s/\tau} \tag{S60}$$

$$\mathcal{I}_2 = \int_{-\infty}^{+\infty} ds\ e^{(W-I)s/\tau}\ \Sigma^{\text{out}} e^{(W-I)s/\tau} \tag{S61}$$

These integrals can be calculated as the solution of the following equations

$$\left(W - I\right)\mathcal{I}_1 + \mathcal{I}_1\left(W^T - I\right) + 2\tau\Sigma^{\text{out}2} = 0 \tag{S62}$$

$$\left(W - I\right)\mathcal{I}_2 + \mathcal{I}_2\left(W - I\right) + 2\tau\Sigma^{\text{out}} = 0 \tag{S63}$$

These can be solved by standard methods for Lyapunov equations.

Another interesting case, which we use in all simulations, is that of colored noise input, which we model as a multivariate Ornstein-Uhlenbeck (OU) process with a covariance that is separable in space and time. The derivations performed above can be generalized to handle such coloured inputs by defining auxiliary variables that independently integrate their

own white noise inputs and feed their outputs (which now have colored OU structure) to the network. Thus, the first block of N variables correspond to the neural network, interacting through the synaptic matrix W, and integrating the input from the auxiliary variables with a time constant τ. The second block of N variables correspond to the auxiliary variables, which integrate white noise of covariance B, with a time constant τ_{in}, and they interact through a mixing matrix Z. The (colored) auxiliary variables are then fed into the neural variables. The mixing matrix Z is set to zero in all simulations implemented here. Under these block ordering conventions, the extended system is described by the following Jacobian:

$$J_e = \begin{pmatrix} \frac{W-I}{\tau} & \frac{I}{\tau} \\ 0 & \frac{Z-I}{\tau_{\text{in}}} \end{pmatrix} \tag{S64}$$

The extended input covariance is equal to $\Sigma_e^{\text{in}}\delta(s)$, with

$$\Sigma_e^{\text{in}} = \begin{pmatrix} 0 & 0 \\ 0 & B \end{pmatrix} \tag{S65}$$

showing that only the second block (auxiliary variables) receives the white noise input. Analogously, the output covariance of the extended system is composed of four blocks:

$$C_e(s) = \begin{pmatrix} C^{\text{out}}(s) & C^{\text{io}}(s) \\ C^{\text{io}}(-s)^T & C^{\text{in}}(s) \end{pmatrix} \tag{S66}$$

Note that since the output of the second block is interpreted as the input to the neural network, we have denoted it by $C^{\text{in}}(s)$. Similarly, the cross-covariance of the two blocks has the same interpretation as the input-output covariance as above, and is therefore denoted by $C^{\text{io}}(s)$. Finally, the top-left block is the covariance of the network output, naturally denoted by $C^{\text{out}}(s)$.

Since the extended system is driven by white noise, the extended output covariance can be calculated analytically, as in Equation S55, and is equal to

$$C_e(s) = \begin{cases} e^{J_e s} C_e(0) & \text{for } s \geq 0 \\ C_e(0) e^{-J_e^T s} & \text{for } s < 0 \end{cases} \tag{S67}$$

where the covariance at zero time lag $C_e(0)$ follows the Lyapunov equation

$$J_e C_e(0) + C_e(0) J_e^T + \Sigma_e^{\text{in}}/\tau_{\text{in}}^2 = 0 \tag{S68}$$

By summing over the corresponding blocks, the first term of the Lyapunov equation $J_e C_e(0)$ is rewritten as

$$\begin{pmatrix} (W-I)\Sigma^{\text{out}}/\tau + \Sigma^{\text{io}^T}/\tau & (W-I)\Sigma^{\text{io}}/\tau + \Sigma^{\text{in}}/\tau \\ (Z-I)\Sigma^{\text{io}^T}/\tau_{\text{in}} & (Z-I)\Sigma^{\text{in}}/\tau_{\text{in}} \end{pmatrix} \tag{S69}$$

where Σ^{out}, Σ^{in}, Σ^{io} are the respective cross-covariances at zero time lag. The following equations follow, one for each block of the Lyapunov equation:

$$(Z-I)\Sigma^{\text{in}} + \Sigma^{\text{in}}(Z^T - I) + B/\tau_{\text{in}} = 0 \tag{S70}$$

$$(W-I)\Sigma^{\text{io}}/\tau + \Sigma^{\text{io}}(Z^T - I)/\tau_{\text{in}} + \Sigma^{\text{in}}/\tau = 0 \tag{S71}$$

$$(W-I)\Sigma^{\text{out}} + \Sigma^{\text{out}}(W^T - I) + \Sigma^{\text{io}^T} + \Sigma^{\text{io}} = 0 \tag{S72}$$

These equations can be solved numerically in order to find Σ^{in}, Σ^{io}, Σ^{out}. The white noise limit corresponds to $Z = 0$ and $\tau_{\text{in}} \to 0$, for which we have $\Sigma^{\text{io}} = B/2\tau$. In this limit, we can recover the Lyapunov Equation S57.

Sequentiality in the colored noise case can be calculated following similar methods as in the white noise case. We start again from Eq.S58, and we note that we can express C^{out} as

$$C^{\text{out}} = P C_e(s) P^T \tag{S73}$$

where $C_e(s)$ is given by Equation S66 and P is a $N \times 2N$ matrix equal with two blocks, $(I0)$, the $N \times N$ identity matrix and the $N \times N$ matrix with all elements equal to zero. Then, substituting the expression for C^{out} in the integrals, we find that sequentiality can be expressed as

$$\text{seq}^2 = \frac{\text{Tr}\left(P\mathcal{I}_1' P^T\right) - \text{Tr}\left(P\mathcal{I}_2' C_e(0) P^T\right)}{\text{Tr}\left(P\mathcal{I}_1' P^T\right) + \text{Tr}\left(P\mathcal{I}_2' C_e(0) P^T\right)}, \tag{S74}$$

where the integrals $\mathcal{I}_1'$ and $\mathcal{I}_2'$ are equal to

$$\mathcal{I}_1' = \int_{-\infty}^{+\infty} ds\ e^{J_e s} C_e(0) P^T P C_e(0) e^{J_e^T s} \tag{S75}$$

$$\mathcal{I}_2' = \int_{-\infty}^{+\infty} ds\ e^{J_e s} C_e(0) P^T P e^{J_e s}. \tag{S76}$$

These integrals can be calculated as the solution of the following equations

$$J_e \mathcal{I}_1' + \mathcal{I}_1' J_e^T + 2C_e(0) P^T P C_e(0) = 0 \tag{S77}$$

$$J_e \mathcal{I}_2' + \mathcal{I}_2' J_e + 2C_e(0) P^T P = 0 \tag{S78}$$

These can be solved by standard methods for Lyapunov equations.

Supplementary Math Note 3 A model of spike timing-dependent plasticity

3.1 Mean field dynamics

We now consider the case in which the dynamics of the synaptic matrix follow spike-timing-dependent plasticity (STDP), and we show that a fixed point of this dynamics corresponds to a time reversible state (Equation S54). We use a variant of STDP that depends on presynaptic spikes and the subthreshold postsynaptic membrane potential (rather than postsynaptic spikes; Clopath et al., 2010, see also Figure 4A). According to this rule, (additive) changes to the synaptic weight between presynaptic neuron j and postsynaptic neuron i occur due to two kinds of contributions. First, at the time of its occurrence, each presynaptic spike causes LTD (a decrease in the synaptic weight) that is proportional to the low-pass (causally) filtered version of the postsynaptic membrane potential at that time. Second, each presynaptic spike also gives rise to an (exponentially decaying) eligibility trace, which continually causes LTP (an increase in the synaptic weight) that is proportional to the momentary product of the eligibility trace and the (unfiltered) postsynaptic membrane potential. Thus, the change in the synaptic weight between the two cells can be described by the following equation:

$$\tau_s \frac{dW_{ij}}{dt} = -W_{ij} + \int_0^{+\infty} dt'\ k(t')\ \delta v_i(t)\, Y_j(t-t') + \int_0^{+\infty} dt'\ k(-t')\ \delta v_i(t-t')\, Y_j(t) \tag{S79}$$

where τ_s is the time constant of synaptic changes, $\delta v_i(\cdot)$ is the (unfiltered) membrane potential of the postsynaptic cell (relative to its long-run time-average), $Y_j(\cdot)$ is the spike train of the presynaptic cell (represented as a sum of Dirac-delta functions), and $k(t)$ for $t \geq 0$ describes the eligibility trace corresponding to a single presynaptic spike, used for determining the magnitude of LTP (described by the first integral), while for $t < 0$ it is the (sign- and time-inverted) kernel with which the postsynaptic membrane potential is filtered, used for determining the magnitude of LTD (described by the second integral).

An example kernel often used in the literature is

$$k(t) = \begin{cases} a_+ \exp(-t/\tau_+) & \text{if } t \geq 0 \\ -a_- \exp(t/\tau_-) & \text{otherwise} \end{cases} \tag{S80}$$

Therefore, if the activity of postsynaptic neuron i is high within a time interval of about τ_+ *following* a presynaptic spike in neuron j, then the synaptic weight increases by an amount proportional to a_+. Conversely, if the activity of postsynaptic neuron i is high within a time interval of about τ_- *preceding* a presynaptic spike in neuron j, then the synaptic weight decreases by an amount proportional to a_-.

While STDP is naturally defined for spiking neurons, e.g. as given by Equation S79, following Kempter et al. (1999); Dayan and Abbott (2001), we use a mean-field approach and describe the average effects of STDP assuming that presynaptic spiking is described by an inhomogeneous Poisson process. Thus, we first average Equation S79 over the distribution of possible spike trains given the underlying presynaptic firing rate time course, and describe STDP dynamics as

$$\tau_s \frac{dW_{ij}}{dt} = -W_{ij} + \int_0^{+\infty} dt'\ k(t')\ \delta v_i(t) f_j\left[v_j(t-t')\right] + \int_0^{+\infty} dt'\ k(-t')\ \delta v_i(t-t') f_j\left[v_j(t)\right] \tag{S81}$$

Second, we also average Equation S81 over the realizations of firing rates and membrane potentials, given their stationary distributions (and in particular, cross-covariances; Equation S29). As a result, STDP dynamics reduces to, in matrix form,

$$\tau_s \frac{dW}{dt} = -W + \int_{-\infty}^{+\infty} ds\ k(s)\, C^{\text{out}}(s)\ \Phi \tag{S82}$$

where we used Bussgang theorem and Φ is the diagonal matrix of average gains (see Section 2.2 and Equation S23). Instead of Equation S82, we consider the following, more general form

$$\tau_s \frac{dW}{dt} = -W + \int_{-\infty}^{+\infty} ds\ k(s)\, C^{\text{out}}(s)\ D \tag{S83}$$

where D is an arbitrary diagonal matrix that effectively gives each presynaptic neuron its own learning rate. The fixed points of Equation S83 are given by

$$W = \int_{-\infty}^{+\infty} ds\ k(s)\, C^{\text{out}}(s)\ D \tag{S84}$$

Thus, the diagonal matrix D plays the role of the "Dale" factor in Equation S54, and indeed we choose it to have a mix of positive and negative elements. Note that a negative element in D for an inhibitory synapse means that the learning rate is $|D|$ but the effective STDP kernel has been sign-inverted relative to that of excitatory synapses. Also note that the covariance $C^{\text{out}}(s)$ depends on W through Equation S32. In order to search for fixed points, we can simulate Equation S83 numerically. Nevertheless, note that Equation S84 itself does not guarantee Hebb-and-Dale connectivity, which would require that weights only depend on the zero-time lag correlations, $C^{\text{out}}(0)$, while here $C^{\text{out}}(s)$ is integrated over time lags s.

Equation S84 corresponds to the condition of time reversibility, Equation S54, which holds for any kernel function $k(s)$, provided that the integral is positive definite. However, stability of the fixed point may depend on the specific choice of $k(s)$. In the following sections we show two cases in which fixed points are described by simple algebraic equations.

3.2 Example 1: linear dynamics and white input

Using the STDP kernel of Equation S80 and the covariance in Equation S55, the integral in Equation S83 can be calculated analytically. Then, synaptic dynamics is equal to

$$\tau_s \frac{dW}{dt} = -W + a_+\tau_+ \left[I + \frac{\tau_+}{\tau}(I-W)\right]^{-1} \Sigma^{\text{out}} D - a_-\tau_-\Sigma^{\text{out}} \left[I + \frac{\tau_-}{\tau}\left(I - W^T\right)\right]^{-1} D \tag{S85}$$

At the time reversible fixed point, Equation S49 holds, which also implies that $W\Sigma = \Sigma W^T$. Substituting this expression in Equation S57, we find that the output covariance has the following expression $\Sigma^{\text{out}} = (I-W)^{-1}\Sigma^{\text{in}}/2\tau$. We further assume that the timescale of potentiation and depression are equal, $\tau_+ = \tau_- = \tau_k$; Then, using Equation S49 in Equation S85 and setting the time derivative to zero, we find that at the time reversible fixed point the synaptic matrix satisfies

$$2W\left(I-W\right)\left[I+\frac{\tau_k}{\tau}\left(I-W\right)\right]=\frac{\tau_k}{\tau}\left(a_+-a_-\right)\Sigma^{\text{in}}D \tag{S86}$$

This equation can be solved numerically by noting that the eigenvectors of W are equal to the eigenvectors of $\Sigma^{\text{in}}D$, and by solving a third order equation for the eigenvalues of W.

3.3 Example 2: linear dynamics and colored input

Using the STDP kernel of Equation S80 and the covariance in Equation S67, the integral in Equation S83 can be calculated analytically and is equal to

$$\int_{-\infty}^{+\infty} ds\; k(s)C_e(s)=a_+\left(\frac{1}{\tau_+}-J_e\right)^{-1}C_e(0)-a_-C_e(0)\left(\frac{1}{\tau_-}-J_e^T\right)^{-1} \tag{S87}$$

Note that only the first block of this matrix needs to be computed in order to simulate synaptic dynamics. The matrix in round brackets can be expressed in block form as

$$\frac{1}{\tau_\pm}-J_e=\begin{pmatrix}\frac{1}{\tau_\pm}+\frac{I-W}{\tau} & -\frac{I}{\tau}\\ 0 & \frac{1}{\tau_\pm}+\frac{I-Z}{\tau_{\text{in}}}\end{pmatrix} \tag{S88}$$

The matrix block inverse is equal to

$$\left(\frac{1}{\tau_\pm}-J_e\right)^{-1}=\begin{pmatrix}\left(\frac{1}{\tau_\pm}+\frac{I-W}{\tau}\right)^{-1} & \frac{1}{\tau}\left(\frac{1}{\tau_\pm}+\frac{I-W}{\tau}\right)^{-1}\left(\frac{1}{\tau_\pm}+\frac{I-Z}{\tau_{\text{in}}}\right)^{-1}\\ 0 & \left(\frac{1}{\tau_\pm}+\frac{I-Z}{\tau_{\text{in}}}\right)^{-1}\end{pmatrix} \tag{S89}$$

Therefore, the first block of the integral in Equation S87 is equal to

$$\begin{aligned}\int_{-\infty}^{+\infty} ds\; k(s)C^{\text{out}}(s)=&\;a_+\left(\frac{1}{\tau_+}+\frac{I-W}{\tau}\right)^{-1}\Sigma^{\text{out}}-a_-\Sigma^{\text{out}}\left(\frac{1}{\tau_-}+\frac{I-W^T}{\tau}\right)^{-1}+\\ &+\frac{a_+}{\tau}\left(\frac{1}{\tau_+}+\frac{I-W}{\tau}\right)^{-1}\left(\frac{1}{\tau_+}+\frac{I-Z}{\tau_{\text{in}}}\right)^{-1}{\Sigma^{\text{io}}}^T-\\ &-\Sigma^{\text{io}}\frac{a_-}{\tau}\left(\frac{1}{\tau_-}+\frac{I-Z^T}{\tau_{\text{in}}}\right)^{-1}\left(\frac{1}{\tau_-}+\frac{I-W^T}{\tau}\right)^{-1}\end{aligned} \tag{S90}$$

This expression, along with Equations S70 to S72 and S83, can be used to simulate synaptic plasticity under colored noise input. Note that this expression reduces to the white noise case for $\tau_{\text{in}}\to 0$.

When time reversibility holds, separately for both the auxiliary variables and the neural network, the covariances can be calculated explicitly, and we have that

$$\Sigma^{\text{in}}=(I-Z)^{-1}\frac{B}{2\tau_{\text{in}}} \tag{S91}$$

$$\Sigma^{\text{io}}=\frac{1}{\tau}\left(\frac{I-W}{\tau}+\frac{I-Z}{\tau_{\text{in}}}\right)^{-1}\Sigma^{\text{in}}=\left(\frac{I-W}{\tau}+\frac{I-Z}{\tau_{\text{in}}}\right)^{-1}(I-Z)^{-1}\frac{B}{2\tau\tau_{\text{in}}} \tag{S92}$$

$$\Sigma^{\text{out}}=(I-W)^{-1}\Sigma^{\text{io}}=(I-W)^{-1}\left(\frac{I-W}{\tau}+\frac{I-Z}{\tau_{\text{in}}}\right)^{-1}(I-Z)^{-1}\frac{B}{2\tau\tau_{\text{in}}} \tag{S93}$$

Furthermore, I assume that $\tau_+=\tau_-=\tau_\pm$ and I define $k_0=a_+-a_-$. Under time reversibility, the integral is equal to

$$\int_{-\infty}^{+\infty} ds\; k(s) C^{\text{out}}(s) = k_0 \left(\frac{1}{\tau_\pm} + \frac{I-W}{\tau} \right)^{-1} \Sigma^{\text{out}} + \tag{S94}$$

$$+ \quad \frac{k_0}{\tau} \left(\frac{1}{\tau_\pm} + \frac{I-W}{\tau} \right)^{-1} \left(\frac{1}{\tau_\pm} + \frac{I-Z}{\tau_{\text{in}}} \right)^{-1} \Sigma^{\text{io}} = \tag{S95}$$

$$= \quad k_0 \left(\frac{1}{\tau_\pm} + \frac{I-W}{\tau} \right)^{-1} (I-W)^{-1} \left(\frac{I-W}{\tau} + \frac{I-Z}{\tau_{\text{in}}} \right)^{-1} (I-Z)^{-1} \frac{B}{2\tau\tau_{\text{in}}} + \tag{S96}$$

$$+ \quad \frac{k_0}{\tau} \left(\frac{1}{\tau_\pm} + \frac{I-W}{\tau} \right)^{-1} \left(\frac{1}{\tau_\pm} + \frac{I-Z}{\tau_{\text{in}}} \right)^{-1} \left(\frac{I-W}{\tau} + \frac{I-Z}{\tau_{\text{in}}} \right)^{-1} (I-Z)^{-1} \frac{B}{2\tau\tau_{\text{in}}} \tag{S97}$$

The time-reversible solution can be found by solving

$$W(I-W) \left(\frac{1}{\tau_\pm} + \frac{I-W}{\tau} \right) \left(\frac{I-W}{\tau} + \frac{I-Z}{\tau_{\text{in}}} \right) =$$
$$= k_0 \left\{ I + \frac{I-W}{\tau} \left(\frac{1}{\tau_\pm} + \frac{I-Z}{\tau_{\text{in}}} \right)^{-1} \right\} (I-Z)^{-1} \frac{BD}{2\tau\tau_{\text{in}}}$$

This is a fourth order equation for W and can be solved by noting that W, Z, BD have the same eigenvectors, and solving numerically the fourth order equation for the eigenvalues of W.

Supplementary Figures

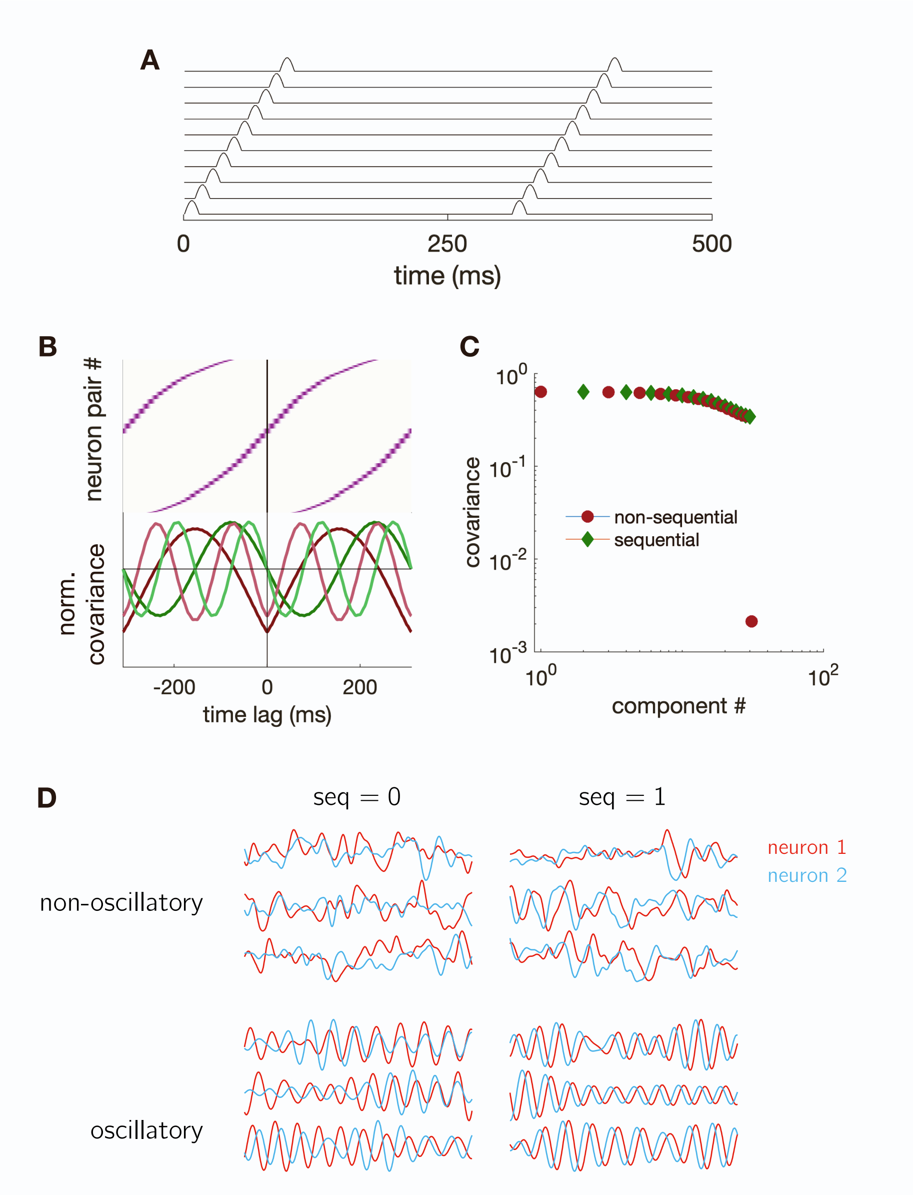

Figure S1: **Examples of sequential and non-sequential activity, and link to synfire chains and neuronal oscillations**, related to Figure 1. **(A)-(C)** A synfire chain (Equation 11; Abeles, 1991) has sequentiality equal to one (seq = 1 in Equations 3 and 10). **(A)** Time series of the activities of the first 10 neurons of a synfire chain (of 31 neurons), showing two activity sweeps across the population. **(B)** Top: time-lagged cross-covariance (CC) functions of the activities of all neuron pairs (as in Figure 1H). Neuron pairs are ordered according to the location of the peak of their cross-covariances. Bottom: first four temporal components (red: symmetric; green: anti-symmetric; as in Figure 1I). **(C)** Sequentiality spectrum (as in Figure 1G): covariance associated with each temporal component of the synfire chain, in decreasing rank order (colors as in the middle panel). **(D)** Our measure of sequentiality is not systematically related to oscillatoriness in neural activity. We consider two neurons whose marginal activity statistics are either smooth but non-oscillatory (top row) or oscillatory (bottom row). The degree of temporal coupling between the two neurons can be controlled independently of these marginals, leading either to zero (left column) or maximal (right column) sequentiality. Three examples are shown for each combination of oscillatoriness and sequentiality. The cases where seq = 1 were constructed following Rutten et al. (2020).

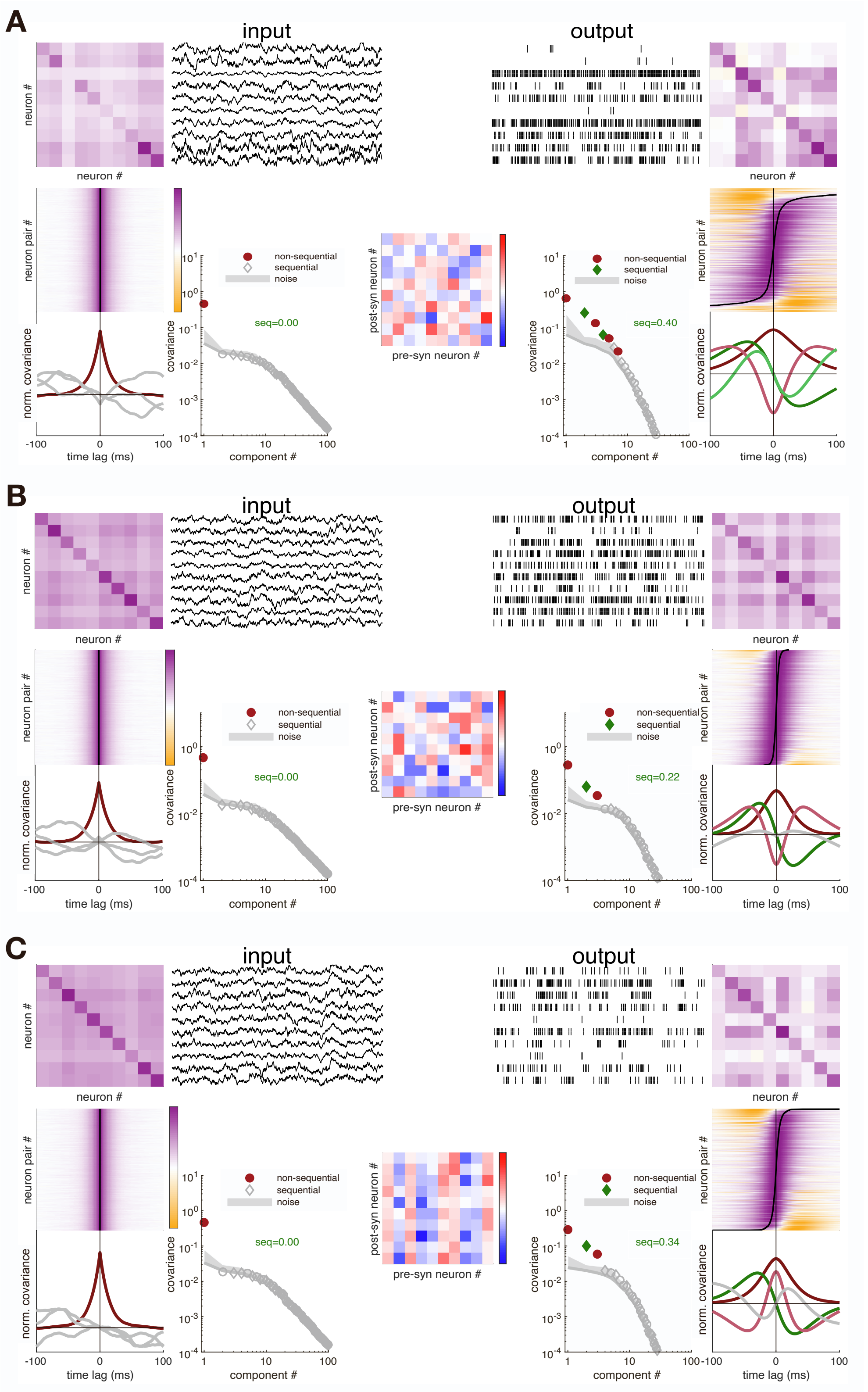

Figure S2: **Sequentiality in example neural circuits with various types of random connectivity**, related to Figure 1. Panels as in Figure 1. **(A)** Random connectivity. Note that, unlike in Figure 1, the connectivity matrix (middle) was not constrained to be symmetric. **(B)** Random antisymmetric connectivity. Note that the connectivity matrix (middle) is skew-symmmetric. **(C)** Random connectivity obeying Dale's law. Note that each column of the connectivity matrix (middle) has all positive or all negative elements.

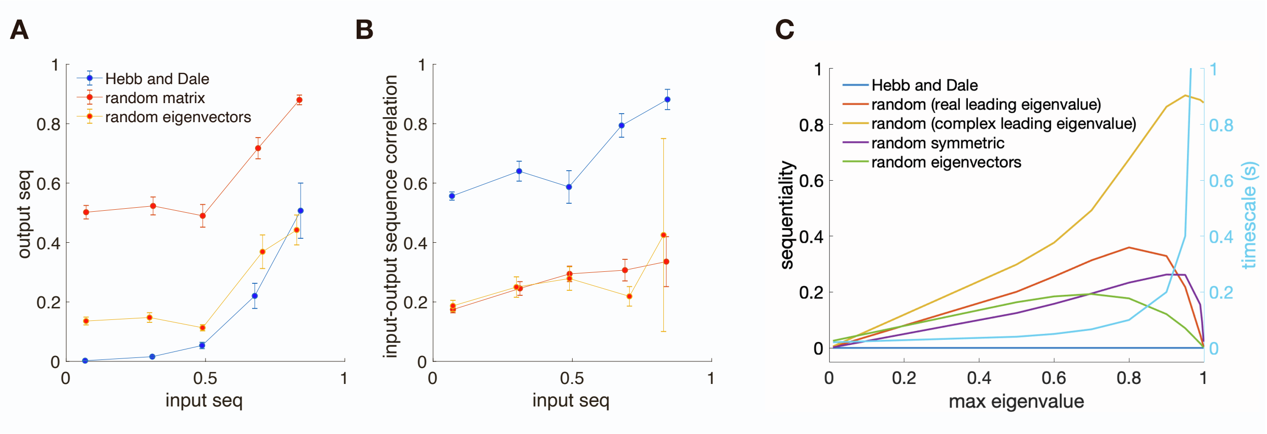

Figure S3: **Hebb-and-Dale connectivity is useful with sequential input or with strong dynamics**, related to Figure 2. **(A)-(B)** We compare networks with three different types of synaptic weight matrices: 1) Hebb and Dale (blue) – as explained in the main text (Figure 2E); random matrix (red) – each entry is drawn independently from a Gaussian distribution of zero mean (Figure S2A; matrix is scaled to have the same spectral abscissa as of the Hebb and Dale matrix); random eigenvectors (orange) – all eigenvalues are identical to those of the Hebb and Dale matrix, but each entry of every eigenvector is drawn independently from a Gaussian distribution of zero mean (scale does not matter). For each connectivity type, we simulated 100 networks at each level of input sequentiality, and show mean$\pm$1 s.e.m. across these networks. **(A)** Output vs. input sequentiality. Sequentiality is computed as explained in the main text. When the input sequentiality is zero, Hebb and Dale connectivity is the only one resulting in zero output sequentiality. When input sequentiality increases, output sequentiality increases for all three types of networks. **(B)** Input-output sequence correlation vs. input sequentiality. Input-output sequence correlation is computed by taking the leading skew-symmetric spatial mode of the input, interpreted as the main sequential input pattern, and finding the highest correlation among the leading 10 output patterns. The Hebb and Dale network achieves significantly higher correlation between the input and output sequential patterns than the other networks. **(C)**: Sequentiality (left y-axis) and network timescale (right y-axis) vs. spectral abscissa (i.e. maximum real eigenvalue) of the synaptic weight matrix, for different types of networks. In addition to those shown in panels (A) and (B), we show results for a *random symmetric* matrix (same as "random matrix" in A-B, but with reciprocal connection strengths set to be equal). In the "random matrix" case, we distinguish sampled weight matrices based on whether their leading eigenvalue (that with maximum real part) is real or complex. All matrices are rescaled by an appropriate factor to obtain the desired value of the spectral abscissa (x-axis). The light blue line shows the corresponding timescale of network integration (i.e. $\tau/\left[1 - \text{spectral abscissa}\right]$, identical for all networks). As we show (and as is well-known; Dayan and Abbott, 2001), across a variety of networks, timescales (and overall fluctuation magnitude, not shown) tend to explode when the connectivity is scaled up towards criticality (spectral abscissa = 1). Yet, we find that this does not affect sequentiality in Hebb and Dale networks (dark blue), as it is provably uniformly zero (Supplementary Math Note 2). However, for more general weight matrices, sequentiality tends to exhibit a maximum at some intermediate connectivity strength (other colors). This can be understood by noting that (i) for weak connectivity, the network does not substantially modify the spatiotemporal structure of the input, which we defined to have seq = 0 in this case, and (ii) as the network approaches criticality, activity becomes dominated by the dominant eigenmode and thus becomes effectively one-dimensional (Ganguli et al., 2008) (except for the case when the leading eigenvalue is complex, yellow) – therefore, by definition, it can only be non-sequential.

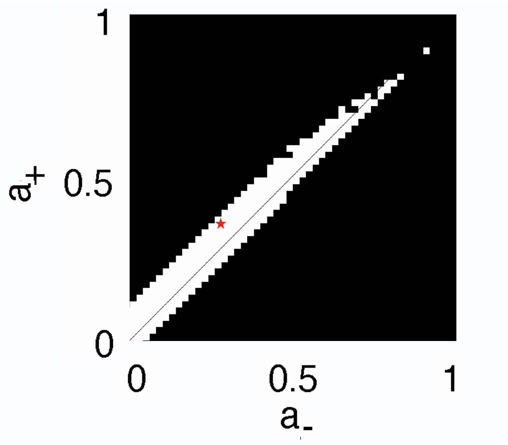

Figure S4: **Stability of STDP learning dynamics**, related to Figure 4. STDP dynamics is stable for a subset of (a_+, a_-) combinations (white region). Red star shows parameter combination used in the simulations of the main text, Figure 4.

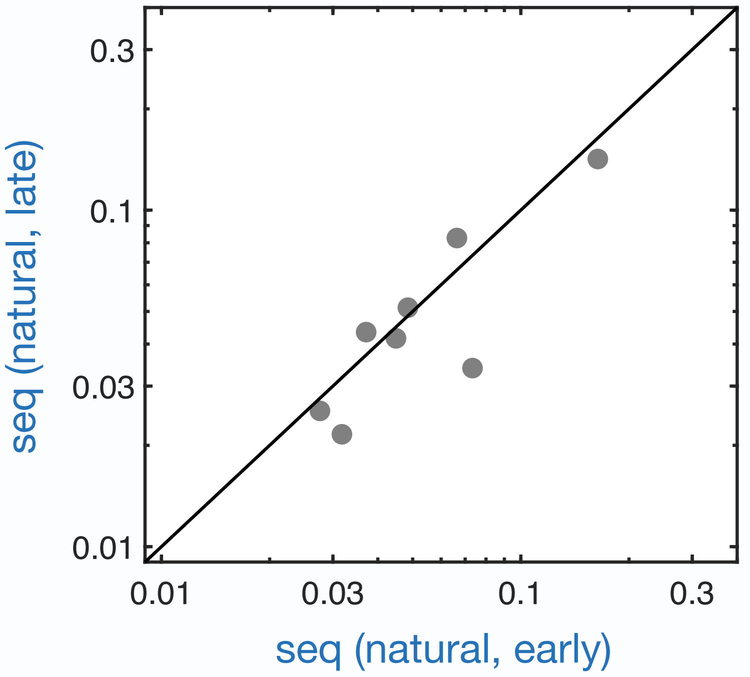

Figure S5: **Temporal changes in sequentiality for natural stimuli.** Same as Figure 5G, but for natural stimuli. Sequentiality of neural responses during the late (y-axis) vs. the early half of exposure (x-axis) to the natural stimuli across animals (dots). Sequentiality is not systematically lower later than earlier, and this does not seem to be due to a ceiling effect (in which case dots with a lower x- or y-coordinate would be closer to the diagonal).
